# Photoactivated Multivariate Metal–Organic Frameworks for On-Demand Drug Release: The Role of Host–Guest Interactions

**DOI:** 10.1021/jacs.4c15222

**Published:** 2025-02-24

**Authors:** Hannah D. Cornell, Abhishek T. Sose, Stefan Ilic, Sreenivasulu Chinnabattigalla, Naomei E. Lidman, Colleen M. Oldmixon, Xiaozhou Yang, Sanket A. Deshmukh, Amanda J. Morris

**Affiliations:** † Department of Chemistry, Virginia Tech, Blacksburg, Virginia 24061, United States; ‡ Department of Chemical Engineering, Virginia Tech, Blacksburg, Virginia 24061, United States

## Abstract

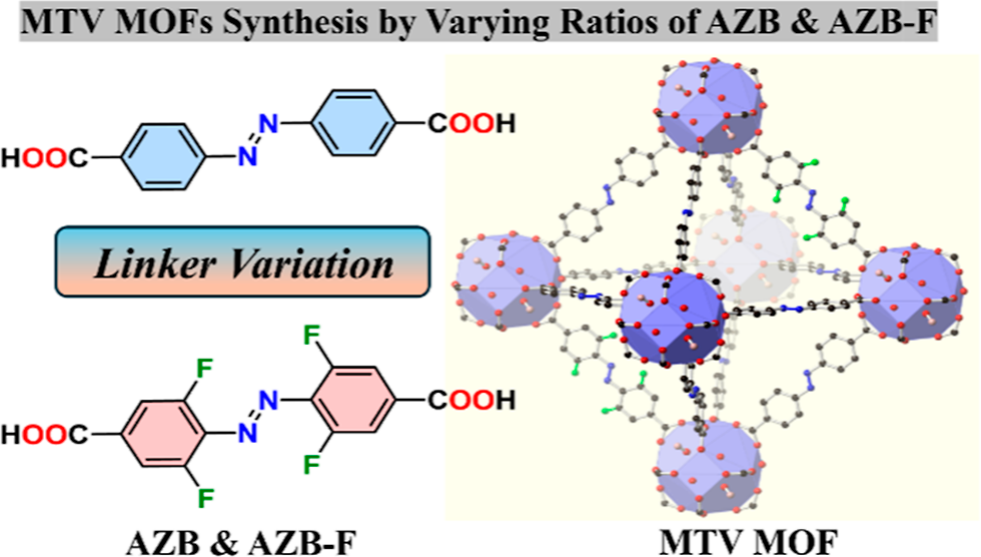

The development of smart drug delivery vehicles capable of controlled release upon application of an external stimulus is of paramount interest for the next generation of personalized medicine. Herein, we report a series of six multivariate (MTV) MOFs capable of visible light-activated drug delivery. The drug loading capacity and release rates were systematically tuned through variation of the linker ratio between 4,4′-azobenzene dicarboxylic acid (H_2_ABDA) and 4,4′-(diazene-1,2-diyl)bis(3,5-difluorobenzoic acid) (H_2_ABDA(3,5-F)). The drug loading capacity, dictated by host–guest interactions, was thoroughly explored via a combined experimental and computational approach using two model drug or drug-like molecules, 5-fluorouracil (5-FU) and Nile Red. Notably, the loading capacity for 5-FU follows a “Goldilocks” profile with a maximum loading at 33% H_2_ABDA(3,5-F) content. Computational results confirm the existence of a cooperative ligand environment that promotes strong, preferential binding at the tetrahedral/octahedral pore window formed between two H_2_ABDA and one H_2_ABDA(3,5-F). Thus, the MTV approach enhanced capacity over the native 100% H_2_ABDA(3,5-F) and 0% H_2_ABDA(3,5-F) MOFs. In addition to increased loading, the rate of cargo release upon green light excitation also increased as the percentage of H_2_ABDA(3,5-F) in the MOF was raised, reaching a maximum release rate of 0.9 ± 0.1% of total cargo per minute for the MOF containing 100% H_2_ABDA(3,5-F) MOF. The results highlight the promise of MTV MOF design for optimizing drug delivery vehicles with relevant payloads and patient-dictated dosing.

## Introduction

Smart drug delivery (SDD) provides a platform for the targeted and controlled delivery of therapeutics. The most advanced examples of SDD vehicles enable spatiotemporal control over drug release, ensuring maximum drug efficacy from limited off-target delivery and appropriate dosage profiles.^1−5^ SDD vehicles rely on an intricate balance of host–guest interactions. Cargo and carriers experience a complex range of physical, chemical, and electrostatic interactions that strongly depend on their structural identities and the surrounding environment.^6−8^ The success of their design hinges on establishing reversible binding mechanisms, which facilitate the successful incorporation and storage of drugs but are concurrently labile enough to allow the eventual release of cargo in response to a stimulus. Some common intermolecular forces exploited in SDD design are van der Waals interactions, π–π stacking interactions, and hydrogen bonding.^9^ Such interactions provide a strong driving force for the incorporation of cargo within a host structure but do not result in covalent attachments, which may alter the drug’s chemical structure and cause irreversible binding to the carrier itself.

Recently, metal–organic frameworks (MOFs) have emerged as promising drug carriers and adsorbents.^10−13^ MOFs have many superior properties, such as precise structural tunability, high accessible porosity, and large surface areas that make them desirable carriers. Moreover, MOF synthetic procedures can be precisely modulated for the production of nanoscale particles required for efficient in vivo transport.^14−16^ Many of the intermolecular interactions between carrier and cargo mentioned above have been designed into MOFs to create materials with enhanced drug adsorption.^17,18^ Favorable MOF-drug interactions can be designed to occur with either the metal node,^19−21^ organic linker, or both.

Relevant to the work presented herein is the use of distinct linker functionalities to promote strong intermolecular interactions with therapeutic cargo. Most commonly, heteroatoms, e.g., nitrogens, are incorporated into MOF linkers to increase hydrogen bonding. Since most pharmaceutical compounds are rich in hydrogen-bonding moieties, this strategy works well for diverse drug classes. Molavi et al. developed a series of UiO-66 type MOFs for selective adsorption of curcumin and methotrexate.^22^ For a series of amine-functionalized UiO-66 (UiO-66-NH_2_) frameworks, an ethylenediamine functionalized UiO-66-NH_2_ (UiO-66-EDA) exhibited the highest adsorption capacity attributed to hydrogen bonding of amino groups on the ethylene diamine with compatible functionalities on the drug compounds. As an alternative to heteroatom incorporation, the increased presence of carbocyclic and heterocyclic^23^ components in drug molecules renders π–π stacking interactions with aromatic linkers an attractive approach to increase cargo capacity within MOFs. Creating larger pore apertures through the introduction of pyrene linkers can increase π interactions and promote the incorporation of hydrophobic cargo.^24^ Perfluorinated MOFs have also shown a high affinity for polycyclic aromatic hydrocarbons through enhanced π–π stacking between the electron-deficient perfluorinated linker and electron-rich drug molecules.^25^ In many cases, multiple types of interactions can be combined in a single material. Ahmadijokani et al. have shown that π–π stacking, hydrogen bonding, and electrostatic interactions play a combined role in the high adsorption capacity of NH_2_-MIL-101 for methotrexate.^26^

With respect to functional group modification, there are methods for further diversifying MOF pore environments and structures. A subclass of MOFs, mixed-linker or multivariate (MTV) MOFs, has been developed to further control material properties without sacrificing the ease of synthetic design. In 2010, Deng et al. created a series of zinc-based MOFs with up to eight distinct linkers in a single framework.^27^ With the addition of various functionalities (–NO_2_, –NH_2_, –Cl, etc.), MTV MOFs showed enhanced selectivity for H_2_/CO_2_ uptake compared to their single component analogs. With up to 400% improvement in selectivity for H_2_/CO_2_, Deng’s MTV MOFs have demonstrated their utility toward performance enhancement. While MTV MOFs have been studied extensively for catalysis and gas storage/separation,^28−30^ relatively few studies have explored their utility in drug delivery applications. Dong et al. first demonstrated the effectiveness of the MTV strategy by designing MIL-101 derivatives that contain functionalized benzene dicarboxylate linkers. The MTV design varied the ratio of an aminated benzene dicarboxylic acid (NH_2_BDC) and naphthalene-1,4-dicarboxylic acid (C_4_H_4_BDC). With increased content of the strongly interacting C_4_H_4_BDC compared to NH_2_BDC, the release rate of doxorubicin and Rhodamine B could be decreased dramatically.^31^ While the release kinetics for the single component MOFs could be used to predict release from MTV–MOFs in Dong’s work, host–guest and guest–guest interactions are often more challenging to model in increasingly complex systems. The field could be advanced tremendously through the aid of computational design. A comprehensive computational model capable of accurately modeling host–guest interactions would provide an invaluable tool for researchers in developing materials with the most potential for success.

**Scheme 1:**
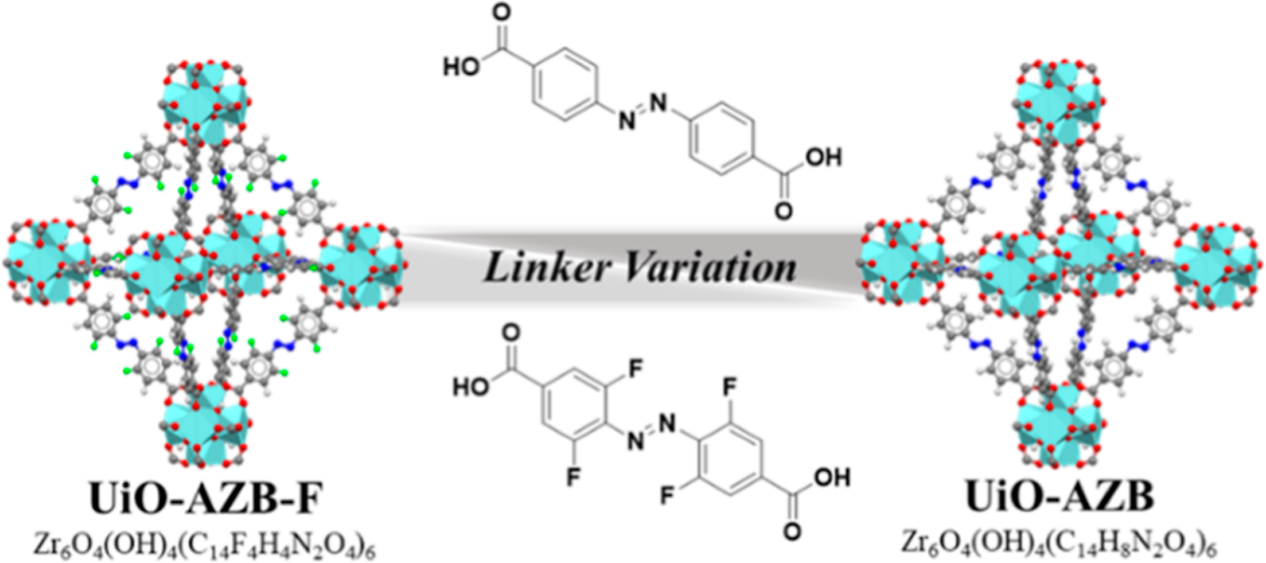
Design Strategy for Fabrication of Multivariate (MTV) MOFs Using Varying Ratios of Photoactivated Linkers 4,4′-Azobenzenedicarboxylic Acid (Top) and 4,4′-(Diazene-1,2-diyl)bis(3,5-difluorobenzoic Acid) (Bottom)

Previously, our lab developed a first-of-its-kind photoactivated MOF drug delivery vehicle (DDV).^32,33^ The DDV photoexfoliated releasing drug and degraded into small molecular components upon the UV light-triggered isomerization of incorporated azobenzene linkers from the thermodynamically stable trans to metastable cis form. Such isomerization imparts significant crystal strain, which is released by MOF breakdown. However, the UV light initially used to trigger release in our first-generation DDV achieves a poor depth of penetration through the skin and limited the overall clinical relevance of the approach. More recently, we addressed this problem through the development of a visible-light responsive MOF containing a fluorinated azobenzene linker (AZB-F) that undergoes photoisomerization with green light irradiation.^34^ To date, fluorous MOFs have been studied for gas adsorption/separation,^35,36^ but no studies have investigated their success as drug carriers. Increased fluorine content could provide a promising strategy for promoting drug adsorption due to the incorporation of strong hydrogen bond acceptors at distinct positions within MOF pores. This work investigates a series of novel photoactivated fluorine-containing MTV MOFs. In these materials, the ratio of H_2_ABDA to H_2_ABDA(3,5-F) in the framework is precisely modified to tune factors such as absolute drug loadings and release rates through the systematic manipulation of host–guest interactions (Scheme 1). To our knowledge, this is the first report of a photoactivated MTV drug delivery vehicle in the literature. The impact of linker composition on cargo loading is explored through a combined experimental and computational approach. Our findings suggest that changing the linker ratio alters the pore environment in a cooperative manner that results in drug loading not predicted by the combination of the adsorption behavior of the two native analogs (UiO-AZB and UiO-AZB-F). In addition to cargo loading, the release rates were tuned through the MTV approach with higher H_2_ABDA content, resulting in slower release profiles. Our work highlights the importance of precision pore environment design as a means to modulate host–guest interactions in MTV MOFs and provides support for the predictive power of computational modeling.

## Results and Discussion

### Synthesis and Characterization of MTV MOFs

A series of MTV MOFs were synthesized by varying the ratios of H_2_ABDA and H_2_ABDA(3,5-F) during a typical UiO-AZB synthesis. More specifically, zirconium chloride (1 mol equiv) was dissolved in DMF with formic acid (20 equiv). To this, a total of 1 mol equiv of the linker was added to the solution (*x* mol H_2_ABDA and *y* mol H_2_ABDA(3,5-F), where *x* + *y* = 1), along with 75 μL of water. The mixture was stirred at 120 °C for 15 min. The resultant powder was collected via centrifugation and washed with DMF and acetone to remove residual linker.

**Figure 1:**
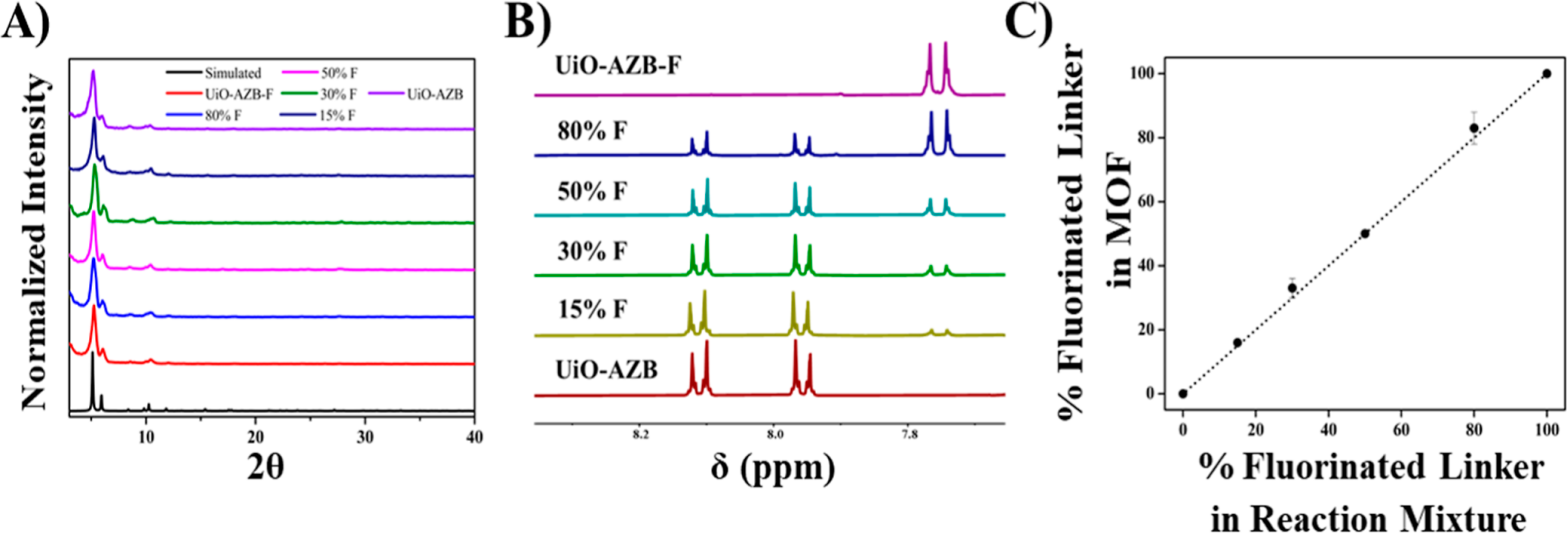
(A) PXRD patterns of MTV MOFs as compared to a simulated pattern for UiO-AZB (CCD 889532) (B) ^1^H NMR of digested MTV MOF samples showing ratios of the incorporated linker (C) Input/Output plot of experimental linker incorporation as determined by ^1^H NMR versus percentage added to reaction mixture reveals a linear relationship.

The crystallinity, size, and composition of the resulting particles were assessed by powder X-ray diffraction (PXRD), scanning electron microscopy (SEM), and ^1^H NMR, respectively (Figures 1 and S14–S18). The experimental PXRD patterns show a sharp peak at a 2θ value of 5.1° followed by a less intense peak at 5.9°, consistent with the simulated pattern for UiO-AZB. As shown in SEM images, the average particle size remains below 150 nm across all MTV MOFs in the series, ensuring particles are of adequate size for drug delivery applications. To quantify the relative amounts of linker incorporated into the framework, particles were digested and analyzed via ^1^H NMR (Figure 1B). Through the integration of the ^1^H signals of H_2_ABDA and H_2_ABDA(3,5-F), the degree of fluorinated linker incorporation was determined. A total of 6 MOFs were obtained, as reported based on H_2_ABDA(3,5-F) content: 100% F, 79(±5)% F, 51(±1)% F, 33(±3)% F, 17(±2)% F, and 0% F. Based on the general molecular formula of the framework (Zr_6_O_4_(OH)_4_(AZB)_6_), these percentages are consistent with incorporation of 6, 5, 3, 2, 1, and 0 fluorinated linker per zirconium node, respectively. A corresponding input/output composition plot was constructed (Figure 1C) and indicated no bias in linker incorporation, as the plot followed a linear trend with a slope of 1.00 ± 0.02 (*R*^2^ value = 0.998). The findings suggest that MOFs of any linker composition can be easily furnished by precisely tuning synthetic conditions.

N_2_ isotherms were collected to probe the impact of linker identity on available surface area and permanent porosity (Figure S22). UiO-AZB-F (100% F sample) exhibited a BET surface area of 1741 m^2^ g^–1^. The remaining derivatives showed significantly higher BET surface area values: 2311 m^2^ g^–1^ (80%), 2267 m^2^ g^–1^ (50%), 2052 m^2^ g^–1^ (30%), 2548 m^2^ g^–1^ (15%), and 2774 m^2^ g^–1^ (0%). After the initial increase in surface area from the 100%–80% samples, surface area values are within error for each of the remaining multivariate MOFs in the series. As expected, UiO-AZB shows the overall highest surface area, as it experiences no blocking of N_2_ adsorption sites due to the absence of larger fluorine atoms. The pore volumes are also similar for all derivatives, ranging from 0.63 to 0.71 cm^3^/g.

TEM–EDS mapping of zirconium and fluorine was conducted to assess the relative distribution of linkers within each MTV framework. In the mapping images, fluorine is evenly distributed across the particle for all MTV derivatives. In addition, EDS spot analysis was used to calculate the relative Zr/F ratio at random locations on different particles. The data is presented in Figures S24–S26. For each MTV derivative, the ratio is consistent with what would be expected based on the MOF molecular formula. Collectively, these data confirm that MTV MOFs contain a random distribution of fluorinated/ancillary ligands throughout rather than forming single-phase regions.

### Cargo Loading of MTV MOFs

**Figure 2:**
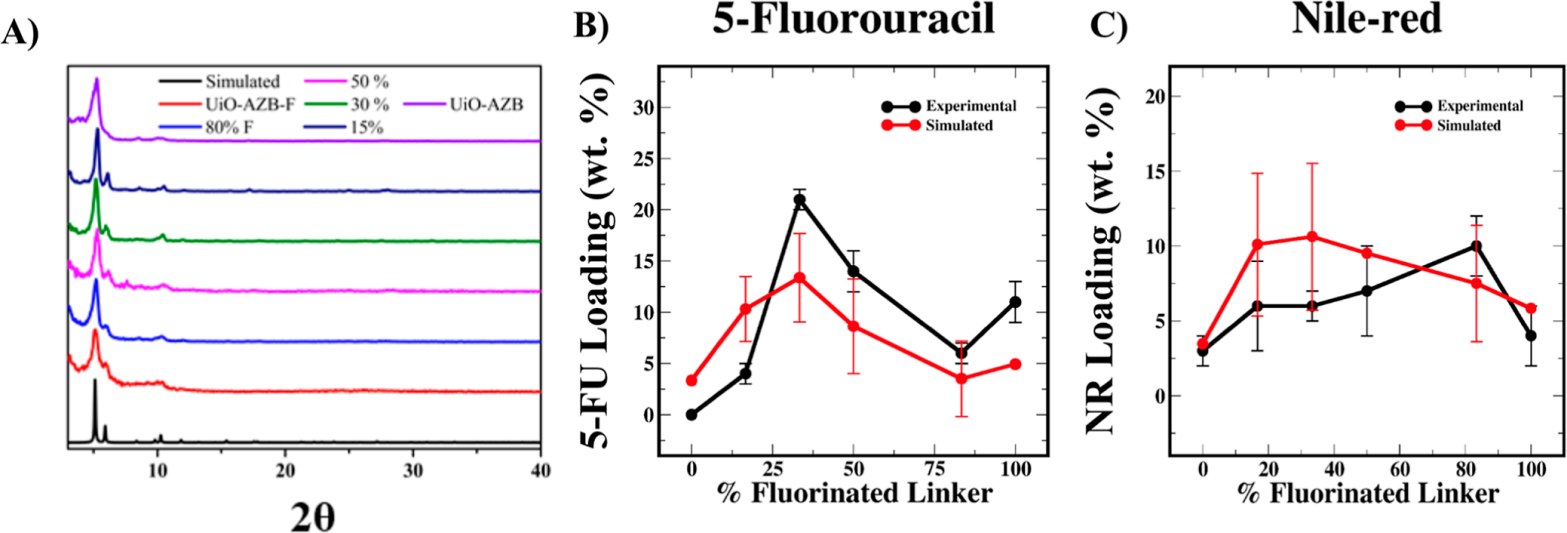
(A) PXRD patterns of MTV MOFs synthesized in the presence of 5-fluorouracil (B) 5-fluorouracil loading as a function of the fluorinated linker content in MTV MOFs. (C) Nile-red loading as a function of the fluorinated linker content in MTV MOFs.

To assess the impact of linker identity on cargo loading, the broad-spectrum chemotherapeutic 5-fluorouracil (5-FU), a hydrophilic chemotherapeutic agent, which provides insights into handling water-soluble drugs with significant clinical applications, was investigated. 5-FU contains multiple functional groups capable of hydrogen bonding with the linkers of the framework and can easily fit within the octahedral pores of the MOF due to its small size (5.4 Å). The compound was incorporated into the framework through an in situ procedure, where 500 mg of 5-FU was introduced into the reaction mixture during MOF assembly. In this work, in situ encapsulation methods were employed to maximize cargo loadings. Previous studies have shown that solvents play an important role in drug adsorption processes,^37^ which limits the encapsulation efficiency of standard impregnation methods used in the literature. Other factors, such as diffusion, drug solubility, and restricted pore windows, also limit cargo incorporation. By synthesizing MOFs in the presence of cargo, many of these issues can be avoided. PXRD patterns of MTV MOFs (Figure 2A) show that particle crystallinity is maintained when 5-FU is introduced within the reaction mixture. After workup, the 5-FU content in each derivative was quantified using ^19^F NMR. The results are shown in Figure 2B, where a maximum 5-FU loading of 21 ± 1 wt % is observed in the 33% fluorinated sample, demonstrating a “Goldilocks” phenomenon. Such a result is not unexpected, as MTV MOFs have been noted to exhibit property enhancements that cannot be easily predicted by looking at their single-component counterparts.

The importance of host–guest interactions was further examined through the loading of a second cargo, Nile Red. Nile Red, being a much more hydrophobic fluorescent dye than 5-FU, shows a different loading trend relative to linker content, which enables the study of encapsulation and the release of lipophilic molecules. The 80% F MTV sample exhibits the highest Nile Red doping (7 wt %) and much more similar loadings across all derivatives. The overall lower Nile Red loading values are attributed to lower initial concentrations of compounds used in the reaction mixture (500 mg 5-FU vs 35 mg NR, as limited by solubility), as well as the larger size of the probe molecule itself (5.4 Å for 5-FU and 12.1 Å for NR).

The nonlinear “Goldilocks” dependence of 5-FU loading with increased fluorine content points to the dominant role of the three-dimensional ligand arrangement in the MTV MOFs and the resulting pore environment on dictating adsorption. We turned to computational modeling to shed further light on the influence of distinct linker assemblies on drug adsorption. Specifically, Grand Canonical Monte Carlo (GCMC) simulations were conducted on selected structures at conditions similar to those in our experiments (Section S2) for all six multivariate MOFs using RASPA 2.0 software and united atom force field (UFF).^38^ Considering the large design space (c.a. Tens of thousands of structures) offered by different arrangements of linkers, selected structures that had unique arrangements of fluorinated linkers in its octahedral and tetrahedral pores were chosen for study (Figures S45–S49). A set of structures was designed and generated based on 16.67% F (4 structures), 33.33% F (4 structures), 50% F (2 structures), and 83.33% F (4 structures), as shown in Figures S46–S49. More details of the MOF structures and the simulation protocol can be found in Section S2.

The simulated adsorption results for all structures within a fluorine-content family were averaged (with equal weighting) and are presented with the experimental data in Figure 2B,C. The simulation results were in qualitative and quantitative agreement with the experimental observations. Deviations from the experimental values most likely resulted from the equal weighting of the potential unique structures to the overall calculated adsorption. Specific geometries may likely be favored either thermodynamically or kinetically during MOF synthesis. Indeed, specific select structures and multiple combinations of structures (as opposed to an equal-weighted average) matched the experimental results perfectly. Still, without an experimental justification for such manipulation of the data, we chose to report the average.

**Figure 3:**
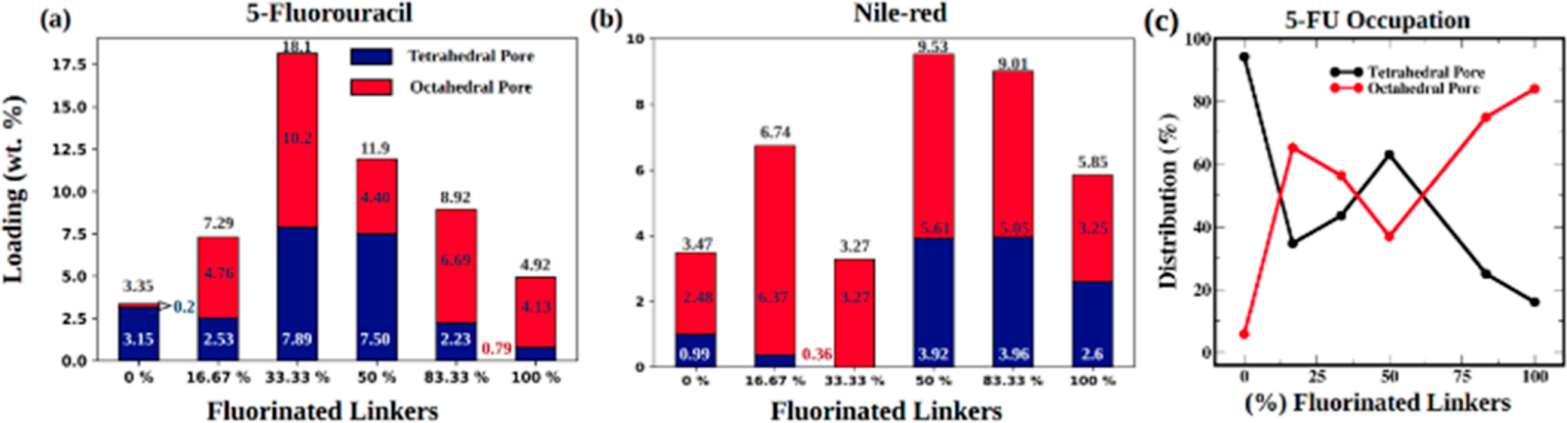
A distribution of (a) 5-FU molecules, (b) Nile-red molecules in tetrahedral pores and octahedral pores, and (c) distribution of 5-FU in tetrahedral and octahedral pores of MTV MOFs.

**Figure 4:**
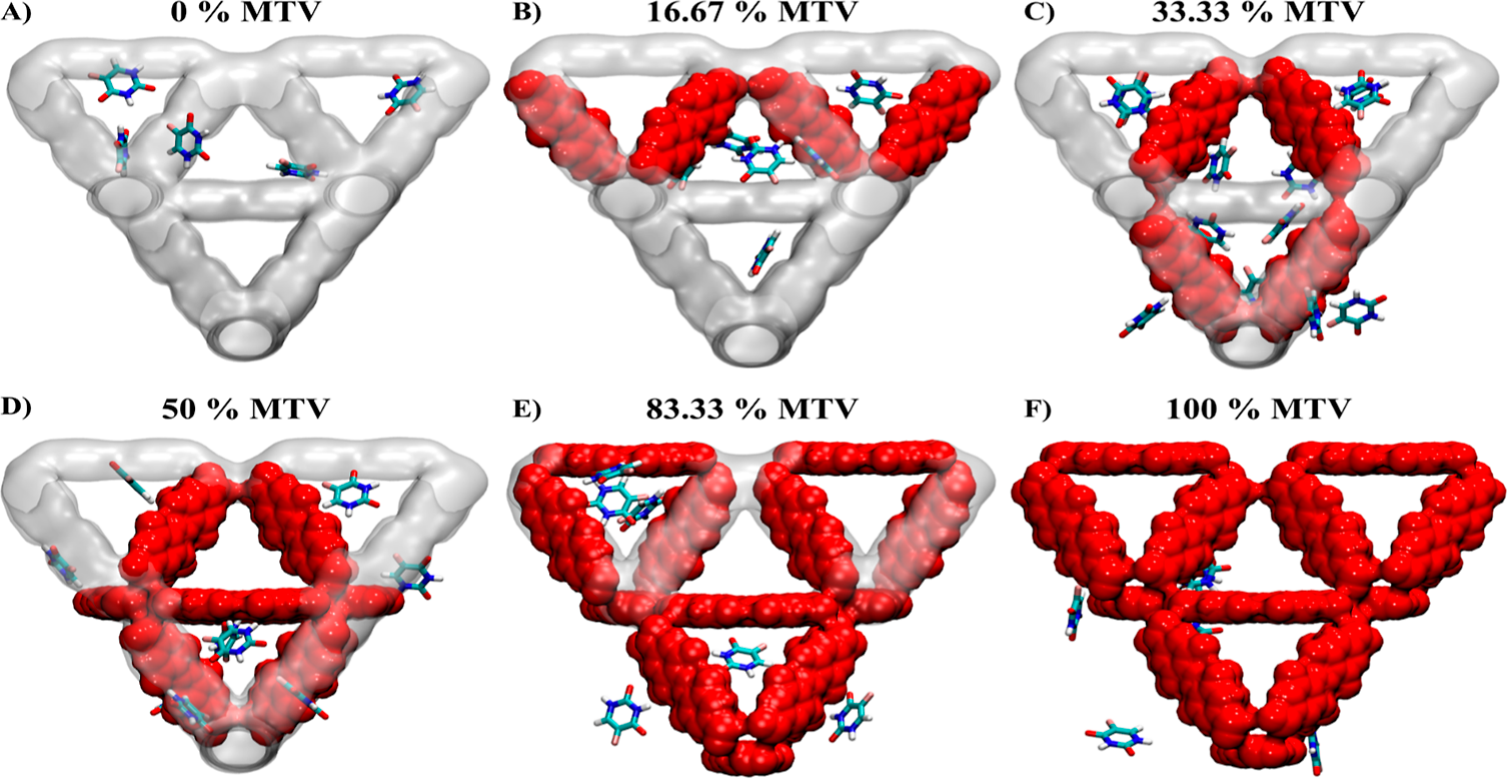
Snapshots of the selected (a-f) 0%–100% MTV MOFs, which exhibited consistency with experimental adsorption of 5-FU molecules.

To determine the precise location of drugs inside the MOFs, an in-depth trajectory analysis was performed by locating the distance of the center of mass of drug molecules from the tetrahedral and octahedral pore centers. We attribute the extensive adsorption in the tetrahedral pore at 0% F to stronger interactions between the linkers and the adsorbate promoted by the smaller pore size and, in turn, the distance between linkers. The additional steric constraints that fluorination imparts in the tetrahedral pore are hypothesized to cause the shift of pore occupation as fluorination is increased. That said, the percentage of tetrahedral pore occupation mirrors the overall adsorption results (save for the 0% F MOF where recall no adsorption was measured experimentally), which points to interactions beyond sterics determining adsorption and may indicate the dominance of tetrahedral pore interactions in determining overall adsorption, Figure 3a,c (snapshots of the 0%–100% F MTV MOFs are depicted in Figure 4). Unlike 5-FU, NR is predominantly adsorbed in octahedral pores, Figure 3b. Due to the larger size of NR compared to 5-FU, it is more likely to be adsorbed into the larger octahedral pore. The rise in the tetrahedral pore occupation with significant fluorination points to the additional attractive forces. While UFF does not explicitly account for electronic level interactions, the observed trends can be attributed to the presence of the electron-rich and electron-pore π systems of the MOF and adsorbate. Therefore, both 5-FU and NR results support the notion that tetrahedral pore interactions are influenced by the MTV approach and dominate adsorption properties.

Further detailed investigation of the localization of 5-FU in MTVs was performed, as shown in Figure S53. In this analysis, we built virtual shells from the center of tetrahedral and octahedral pores, to estimate the average number of drugs that reside in them and their approximate location (close to the linker or at the center of the pores). The distribution indicated that the majority of 5-FU adsorption occurred in the vicinity of linkers. Moreover, MOFs with the highest 5-FU uptake, 7.89% and 7.5% for 33.33% F and 50% F MTV MOFs, respectively, demonstrated a major portion of the adsorption at the linkers (Figure 3). Furthermore, the analysis indicated adsorption at sites not captured within virtual shells. These sites are primarily those at the windows between the tetrahedral and octahedral pores and the pocket formed near the metal nodes. Notably, the percentage of 5-FU adsorption at these sites was highest for 33.33% F configurations. At first glance, it is evident that 5-FU adsorption increased in metal node pockets and the peripheral part of tetrahedral and octahedral pores as the fluorination increased.

**Figure 5:**
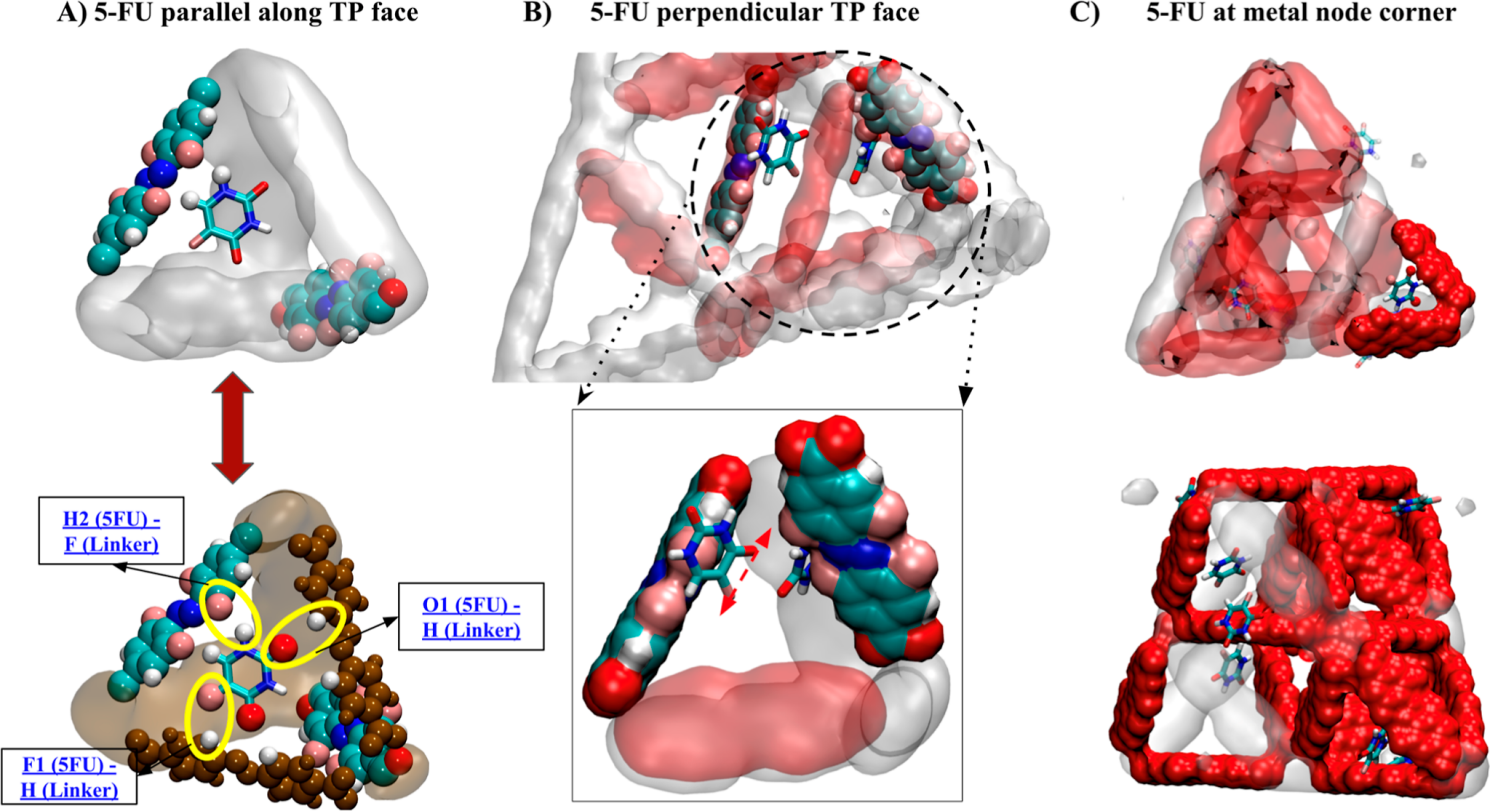
Snapshots of the sites where adsorption occurred and the orientation of 5-FU in (a) 16.67% MTV MOF, (b) 33.33% MTV MOF, and (c) 50% MTV MOF. The top and bottom rows show the same figure with different angles and ways of representation of atoms. The pink, red, white, and blue atoms denote fluorine, hydrogen, oxygen, and nitrogen atoms, respectively.

**Figure 6:**
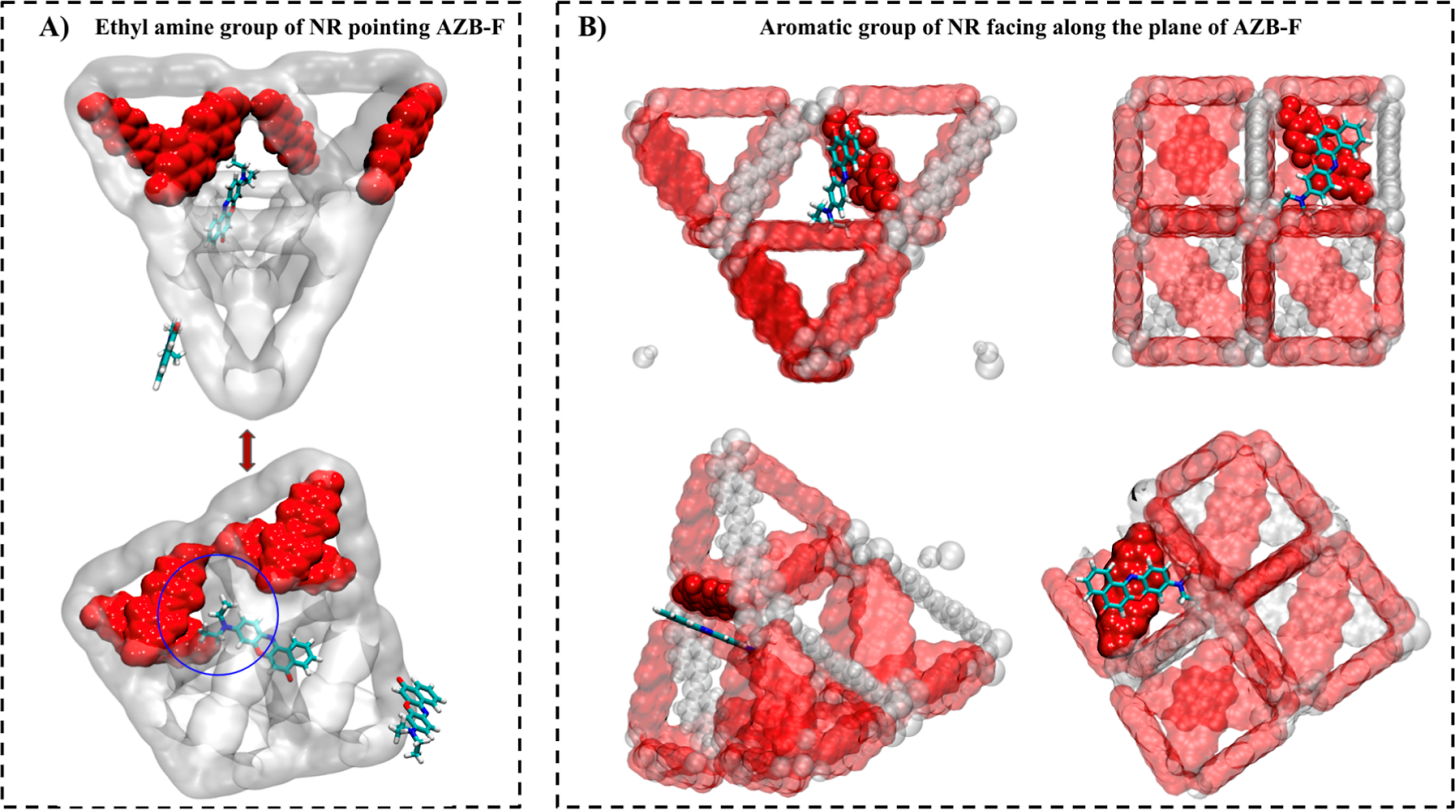
Snapshots of the sites where adsorption occurred and the orientation of NR in (a) 16.67% MTV MOF, (b) 83.33% MTV MOF. The top and bottom rows show the same figure with different angles and ways of representation of atoms. The pink, red, white, and blue atoms denote the fluorine, hydrogen, oxygen, and nitrogen atoms, respectively.

The orientation of and precise adsorption sites for 5-FU (as shown in Figure 5) and NR (as shown in Figure 6) provide additional insight into cooperative binding effects and ligand arrangements/populations that result in favorable interactions. Analysis of the trajectories of 5-FU in different unit cell arrangements shows three significant interactions that potentially drive adsorption. The observation of 5-FU localization on the window between the tetrahedral and octahedral pores was particularly relevant to the MTV approach. The window-adsorption phenomena occurred only when one of three linkers of the face was fluorinated, while the other two were nonfluorinated, as shown in Figure 5a, where red and white (brown) linkers denote H_2_ABDA(3,5-F) and H_2_ABDA, respectively. In particular, the hydrogens H1 and H2 (denoted in white) were aligned with the direction of F (denoted in pink) in H_2_ABDA(3,5-F), whereas O2 (denoted in red) and F1 pointed toward H in one H_2_ABDA and O1 toward H in another H_2_ABDA as shown in Figure 5a. The Supporting Information (Movie M1) illustrates the trajectory of one 5-FU molecule located on the face of tetrahedral pores (TP), also showing the distances between the above-selected atoms with H and F of MOF. The residence time of the 5-FU molecule at this site indicates a clear preference for this binding geometry over other potential configurations, suggesting a stronger interaction relevant to the other observed binding geometries. Since this pore configuration is favored at 33% incorporation, this preferred geometry likely dictates the observed “Goldilocks” phenomenon.

As the percentage of linkers within a pore or window shifted to increased H_2_ABDA(3,5-F) content, the cooperative window-bound species decreased. We attribute this to the steric hindrance imparted by the additional F atoms. Interestingly, the trajectories now reveal a noncooperative binding mode, where the 5-FU is oriented perpendicular to the plane of an H_2_ABDA(3,5-F) linker, as shown in Figure 5b. This stronger interaction can be explained by the hydrogen bond formation between N1–H2···F and N2–H3···F. The residence time at any one H_2_ABDA(3,5-F) was small (relative to the window geometry), and the 5-FU molecules sampled multiple equivalent sites within the computational time frame.

A similar analysis was conducted to determine Nile Red (NR) adsorption sites in MTV MOFs. As expected, the binding geometries and trends differed significantly from those observed for 5-FU. At low concentrations of H_2_ABDA(3,5-F), the NR molecule primarily resided in the octahedral pores, with its diethylamine group directed toward the fluorinated linker, while the opposite end pointed toward the nonfluorinated octahedral pores (OPs). However, with increased fluorinated linker (H_2_ABDA(3,5-F)), the NR molecule’s location shifted significantly, moving to the window between the octahedral and tetrahedral pores. Notably, the aromatic ring of NR was observed to align parallel to the plane of the H2ABDA(3,5-F) linker, as shown in Figure 6b.

As depicted in Figure 3b, NR adsorption increased significantly in the tetrahedral pores for MTV MOFs containing 50% or higher H_2_ABDA(3,5-F) content. Specifically, NR adsorption in the tetrahedral pores was driven by the presence of at least one H_2_ABDA(3,5-F) linker. At even higher H_2_ABDA(3,5-F) content (83.33% and 100% MTV MOFs), the fluorine atoms of the AZB linker showed a stronger structural correlation with the aromatic part of NR, with first peaks of O2–F occurring at 3.78 and 2.92 Å, respectively. These findings contrast the behavior observed at lower H_2_ABDA(3,5-F) content, where the diethylamine group was positioned closer to the MOF surface. This shift indicates a reversal in NR orientation at higher H_2_ABDA(3,5-F) content, with the aromatic rings now positioned closer to the MOF surface. Interestingly, higher adsorption was observed in both the OP and TP regions for the fully fluorinated 100% F MTV. In this case, while adsorption was primarily concentrated in the TP, the larger red portion of the graph (denoting adsorption in OP) in Figure 3b resulted from the NR molecule located at the boundary between the OP and TP. Since the NR molecules are relatively large, their center of mass (COM), which straddles the boundary between the TP and OP, may fluctuate, thus increasing the apparent adsorption in the OP region. To gain a more detailed understanding of NR adsorption, the atom-wise distribution of NR molecules was plotted in Figure S61a. The results showed a larger purple bar, representing higher adsorption (i.e., a greater number of NR atoms) near the MOF metal nodes and along the periphery of both OP and TP. These observations support the hypothesis that fluorination enhances NR adsorption in TPs relative to OPs. This conclusion is further reinforced by the stronger correlation between O1 and O2 atoms and the MOF atoms, as observed in Figure S58e,f. Overall, snapshots and movies illustrating NR adsorption in all MTV MOFs are presented in Figures S62–S67, and the corresponding trajectories can be seen in Supporting Information (Movies M2–M7).

**Figure 7:**
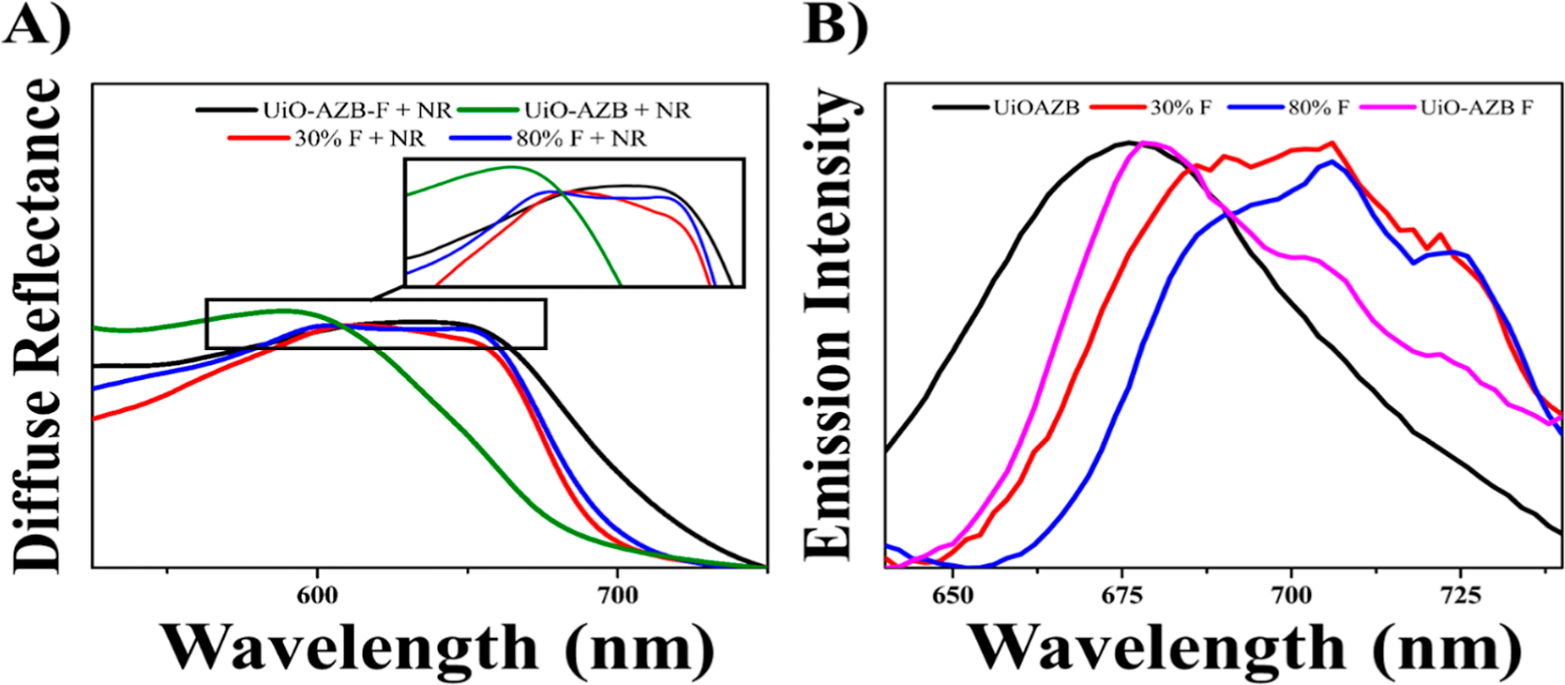
(A) Diffuse reflectance spectroscopy of Nile Red loaded MTV MOF samples (B) fluorescence emission profiles of select Nile Red loaded MTV MOFs (excitation wavelength: 600 nm).

We employed steady-state electronic absorption and fluorescence spectroscopy to provide further support for the environment about the NR molecules within the MOF. The spectroscopic properties of NR are highly dependent on the polarity of the environment and the concentration of NR within that environment.^39−41^ Specifically, NR absorption and emission are known to shift to longer wavelength (to the red) in both polar environments and with higher concentrations due to the formation of aggregates. The diffuse reflectance spectra for select NR-loaded MTV MOFs are shown in Figure 7. The absorption maxima for the MOFs were 600 nm for 0% F and 650 nm for 30% F, 80% F, and 100% F. For comparison, the absorption spectrum of a dilute NR ethanol solution exhibits a maximum at ∼550 nm, which shifts red to ∼595 nm and broadens at higher concentrations.^42^ Therefore, the maxima’s appearance at longer wavelengths indicates a highly polar pore environment and/or aggregation of the NR in the MOF pores. The broad nature of the adsorption profile certainly supports the occurrence of aggregation.

The emission spectra of the MOFs support a similar conclusion: a highly polar pore environment and potential aggregation. The emission maxima for NR were 676 nm for 0% F, 706 nm for 30% and 80% F, and 680 nm 100% F. These features are red-shifted in comparison to the emission of NR in ethanolic solution, which is typically around ∼646 nm, appearing as a sharp, featureless peak. In addition, the 30%, 80%, and 100% F samples exhibited vibrational features within the emission spectra, indicating a more confined environment and/or reduced degrees of freedom.^43,44^ Such phenomena are expected when molecules are trapped in nanoconfined environments and previously observed in photoactive MOFs.^45^ The nonlinear trends in both the emission maxima and the prevalence of vibrational signatures are of interest and suggest that it is not simple confinement that explains the behavior (as one would expect nanoconfinement effects to become more prevalent with increased H_2_ABDA(3,5-F) content). Therefore, the NR molecules in MTV MOF compositions are hypothesized to exhibit stronger binding interactions with the linkers, leading to more locked geometries and aggregation. Considering little-to-no NR–NR interactions/aggregation were observed in the computational NR trajectories, we postulate that the aggregation signatures result from strong linker-NR interactions.

### On-Demand Release from MTV Frameworks

**Figure 8:**
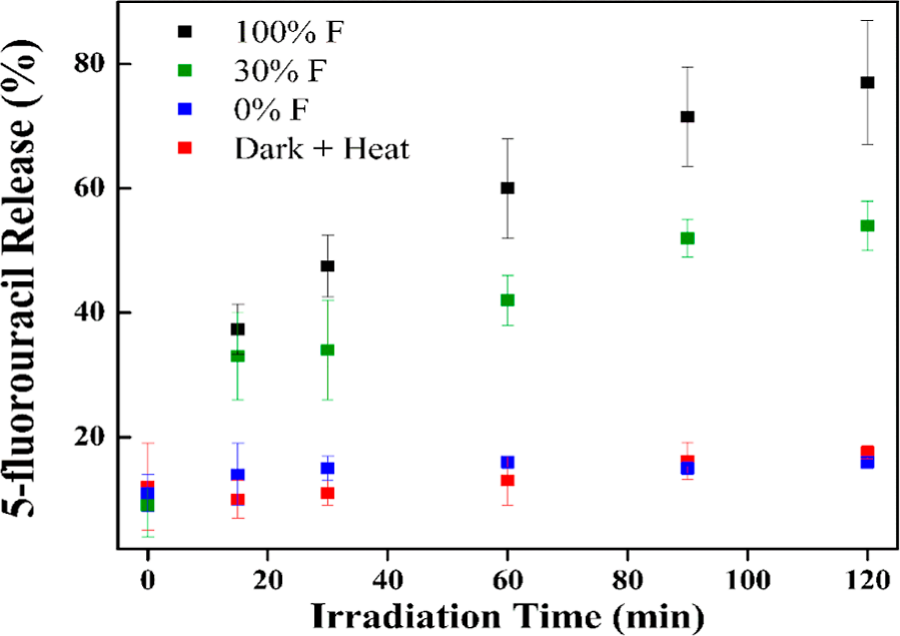
Release profiles of 5-FU from select MTV MOF derivatives.

The cargo was also tuned using the MTV approach. To load 5-FU into the MOFs for drug release, a postsynthetic loading was used (see Supporting Information for details). While postsynthetic loading results in overall lower loading, 5-FU content is incorporated into all the MOF variants (0% F to 100% F) and therefore, provided a better basis for comparison. For the release studies, all particles were also postsynthetically functionalized with amine-terminated poly(ethylene glycol) (PEGNH_2_). Polymer surface coatings, such as PEGNH_2_, are commonly utilized to attenuate burst release from MOF particles.^33,34,46,47^ After polymer functionalization, the MOF particles were immersed in D_2_O with internal standard (more details in ES) and irradiated with green light LED (515 nm) for a period of 2 h, resulting in the conversion of the trans isomer of the azobenzene linker to the cis isomer. This structural change led to the collapse of the MOF framework and the subsequent release of the drug cargo.The amount of 5-FU released into the supernatant was quantified using ^19^F NMR. The release data for the 100% F, 33% F, and 0% F samples are shown in Figure 8. All samples showed ∼10% release of 5-FU at time zero, which is attributed to burst release from uncoated particles in solution. Over the irradiation period of 2 h, 77 ± 10% of 5-FU was released from a 100% F sample, 54 ± 4% was released from a 30% F sample, while the 0% sample shows no additional release after the initial burst. As the amount of fluorinated linker incorporated into the MOF decreased, the total amount of 5-FU released under equivalent irradiation decreased. The initial release rates were estimated over the first 60 min of irradiation. The 100% F sample shows an average release of 0.9 ± 0.1% of total cargo per min^–1^, nearly 50% faster than the 30% F sample (0.7 ± 0.1% of total cargo per minute). The effect is due to the change in absorptivity of the sample at the excitation wavelength, which decreases as H_2_ABDA(3,5-F) content decreases. Our previous studies show that H_2_ABDA(3,5-F) exhibits an extinction coefficient of ∼772 M^–1^ cm^–1^ at 515 nm, whereas H_2_ABDA is nonabsorptive at that wavelength.^34^ Since light absorption and the resultant isomerization of the azo-unit of the linker are the triggers for cargo release it follows that more absorptive samples would release more rapidly. Accordingly, the release profile of the 0% sample overlays that of control experiments for the 33% F and 100% F samples when heated at 37 °C and kept in the dark.

## Conclusions

In conclusion, a series of MTV MOFs were designed as photoactivated MOF drug delivery platforms. By varying the proportion of fluorinated organic linkers, the cargo loadings and release rates could be precisely tuned. In particular, the 33% F derivative showed the highest affinity for 5-FU experimentally and computationally. GCMC simulations suggest that specific linker configurations create preferential binding pockets for 5-FU molecules, increasing the absolute loading. Namely, interactions facilitated at the tetrahedral/octahedral pore window between 5-FU and two H_2_ABDA and one H_2_ABDA(3,5-F) linkers were evident. Conversely, the loading of NR showed a near consistent loading across the MTV MOFs due to two competing interactions with the diethylamine group’s orientation toward the fluorinated linker and the π–π interactions between NR’s aromatic ring and the plane of the H_2_ABDA(3,5-F) linker. The results not only highlight the importance of host–guest interactions and binding locations identified via computational analysis, but also demonstrate the promise of the MTV approach in developing optimized SDDs. While our current work focuses on demonstrating the loading and release of a 5-FU, we see significant potential for extending this approach to a broader range of therapeutic agents. The molecular tunability of MOFs enables the design of specific pore structures, drug–linker interactions, and degradation mechanisms, which can be exploited to deliver a range of therapeutics for the treatment of many diseases. Exploring such applications, including drug combinations, will be the focus of future investigations.

## Supporting Information Available

Experimental procedures for MOF synthesis; spectroscopic characterization; powder diffraction crystallography; scanning electron microscopy; thermal gravimetry; drug release studies; GCMC simulations (PDF)
The Movie M1 illustrates the trajectory of one 5-FU molecule located on the face of TP also showing the distances between above selected atoms with H and F of MOF. The corresponding trajectories can be seen in Movies M2–M7 (ZIP)
