# Supplementary material for: Photoactivated Multivariate Metal–Organic Frameworks for On-Demand Drug Release: The Role of Host–Guest Interactions
Source: J Am Chem Soc. 2025 Feb 24;147(9):7423–32. doi: 10.1021/jacs.4c15222 (PMC11887053; doi:10.1021/jacs.4c15222)
Supplement: Supplementary file 1 — ja4c15222_si_001.pdf [file ja4c15222_si_001.pdf]

Photoactivated Multivariate Metal-Organic Frameworks for On-Demand Drug Release: The Role of Host-Guest Interactions

Hannah D. Cornell[1#], Abhishek T. Sose[2#], Stefan Ilic[1], Sreenivasulu Chinnabattigalla[1], Naomei E. Lidman[1], Colleen M. Oldmixon[1], Xiaozhou Yang[1], Dr. Sanket A. Deshmukh[2], and Dr. Amanda J. Morris[1]

[1] *Department of Chemistry, Virginia Tech, Blacksburg, VA, USA, 2461*

[2] *Department of Chemical Engineering, Virginia Tech, Blacksburg, VA, USA, 2461*

[#] *Both authors contributed equally*

*Corresponding author: ajmorris@vt.edu

Table of Contents

Materials…………………………………………………………………………………....2

Characterization Methods………………………………………………………….……2-4

Synthesis/Characterization of AZB-F…………………………………………..…..……4-6

Synthesis/Characterization of multivariate MOFs.………………...………………..……..7-19

Drug loading (5-FU & Nile-red) and Release studies…..……..……………..……..……..19-33

Simulation details..………..……………..……..……………………………..……..…….33-52

References…………………………………………………………………………………52-52

S1. Experimental

I. Materials

The following chemicals were purchased from Sigma-Aldrich (St. Louis, MO, USA): sodium iodide, ZrCl4 (≥99%), and Poly(ethylene glycol) bis(3-aminopropyl) terminated (Mn ~1,500). Ethyl 4-amino-3,5-difluorobenzoate (≥95%) was purchased from Synthonix (Wake Forest, NC). Formic acid, potassium hydroxide, and dimethyl sulfoxide were obtained from Oakwood Chemical (Estill, SC, USA). 5-fluorouracil (≥99%), ethanol, glacial acetic acid, hydrochloric acid, tetrahydrofuran (THF, ACS grade), ethyl acetate (ACS grade), dichloromethane (DCM, ACS grade) and *N*,*N*-dimethylformamide (DMF, ≥99.8%) were obtained from Fisher Scientific (Hampton, NH, USA).

II. Characterization Methods

Nuclear Magnetic Resonance (NMR) Spectroscopy

All ^{1}H and ^{19}F NMR experiments were performed using an Agilent U4-DD2 (400 MHz) instrument. For MOF digestion samples, 2 drops of H_2SO_4 were added to 2–5 mg of particles suspended in d_6-DMSO (650 µL) in order to fully degrade the particles for quantitative assessment. For quantifying polymer coating, the relaxation delay was increased to 8 s. For ^{19}F quantitative NMR (5-fluorouracil content/release), 64 scans and a relaxation delay of 10 s was used.

Powder X-Ray Diffraction (PXRD)

PXRD measurements were performed using a Rigaku Miniflex diffractometer (Cu Kα radiation λ= 1.5418 Å). Powder samples were loaded on a Rigaku Si510 sample holder disc and analyzed at a 0.05° resolution and a 5.0°/min continuous scanning mode over 2θ = 2−50°.

UV-Vis Spectroscopy

Absorbance measurements were taken using a Cary 5000 UV-Vis-NIR spectrometer controlled with Cary WinUV software. The scan application was used to collect spectra from 250-700 nm to

characterize 4,4’-(diazene-1,2-diyl)bis(3,5-difluorobenzoic acid) and measure degradation of UiO-AZB-F upon green light irradiation.

Thermogravimetric Analysis (TGA)

Thermogravimetric Analysis was performed with a TGA 550 thermal analyzer (TA Instruments, New Castle, DE, USA). In a typical experiment, samples were heated at a ramp rate of 10 °C/min under nitrogen from 25–800 °C. All data was analyzed in TRIOS software (TA Instruments, New Castle, DE, USA) using the weight change function. A mass loss for drug cargo and MOF were obtained and used to calculate experimental drug loadings. The temperatures used for these calculations are included beneath each TGA plot.

N_2 Adsorption Isotherms (BET Surface Area Analysis)

N_2 adsorption and desorption isotherms were collected using a Micrometrics 3-Flex surface analyzer (Micromeritics, Norcross, GA, USA). To access permanent porosity, samples were solvent exchanged in acetone for 3 days. The solvent was refreshed each day. Prior to analysis, samples were placed in sample cells and activated under vacuum (0.1 mbar) for 24 h at 120 °C. After analysis, PXRD was performed on each of the samples. PXRD data confirms that crystallinity was maintained throughout the activation/adsorption process.

Dynamic Light Scattering (DLS)

The size distribution of the UiO-AZB-F nanoparticles was measured using a Malvern ZetasizerNano-ZS, with three measurements taken per trial. Nanoparticles were suspended in ethanol via sonication and passed through a 0.45 µm filter prior to measurement. Particle size and zeta potential values represent the average of 3 distinct samples (2+ replicates per sample).

Scanning Electron Microscopy (SEM)

Silicon wafers were adhered to SEM stages *via* double-sided copper tape. MOF samples were suspended in ethanol solution and drop-cast onto the wafer and allowed to evaporate in air. SEM stages were coated with a 7 nm thick Pt and Pd layer prior to analysis. SEM images were collected with a LEO 1550 field-emission scanning electron microscope (Car Zeiss, Oberkochen, Germany) at 5.0 kV and a 7.0 mm working distance.

Transmission Electron Microscopy (TEM)

MOF samples were suspended in ethanol solution and drop-cast onto a lacey carbon TEM grid (300 mesh, 150 micron) and allowed to evaporate in air. TEM and EDS images were collected with a JEOL 2100 electron microscope at 200 kV.

III. Synthesis/Characterization of Linker

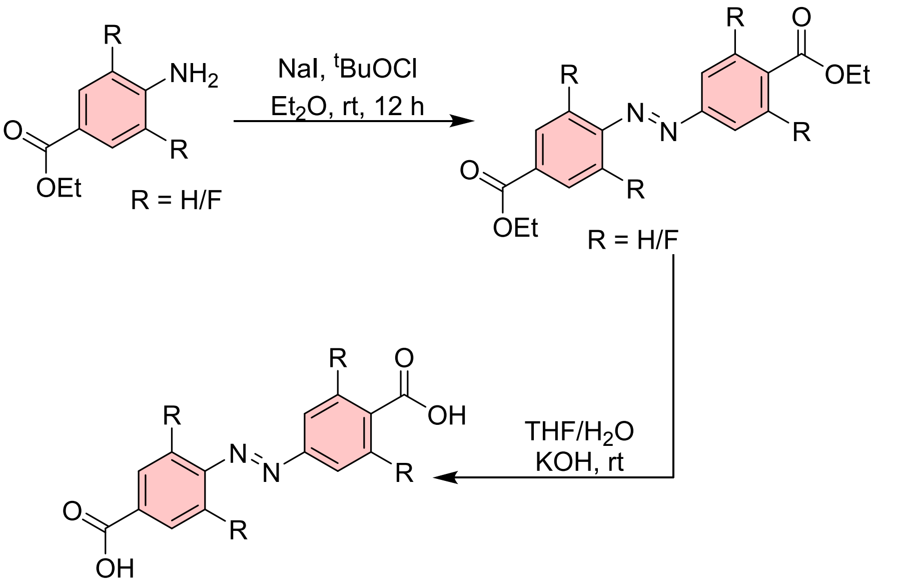

Scheme S1: Synthesis of 4,4’-(diazene-1,2-diyl)bis(3,5-difluorobenzoic acid)

Synthesis of diethyl 4,4’-(diazene-1,2-diyl)€-bis(3,5-difluorobenzoate)[1]

In a 100 mL round-bottom flask, ethyl 4-amino-3,5-difluorobenzoate (3.996 g, 19.9 mmol), NaI (5.97 g, 39.8 mmol), and Et_2O (60 mL) were combined. The flask was sealed and placed under argon atmosphere. Next, *tert*-butyl hypochlorite (4.5 mL, 39.7 mmol) was added dropwise over a period of 10 min. The reaction was stirred under argon flow for 30 min, then removed and stirred for an additional 12 h at room temperature. The reaction was quenched with a 1.0 M aqueous sodium thiosulfate solution (200 mL) and extracted with DCM (2 × 100 mL). The organic layers were collected, dried over calcium chloride and evaporated to obtain a reddish-brown solid (3.01 g crude yield). The solid was dissolved in EtOAc (70 mL) and, after being placed in a freezer overnight, a red-orange precipitate was collected. The product was further purified using a silica plug (solvent system: DCM/pentane 50/50), yielding the desired product as a dark red solid (1.89 g 48% yield).

R_f value (DCM/hexane 1:1): 0.11. **Isomeric ratio:** 91:9 **^{1}H NMR** ($CDCl_3$, 400 MHz): δ major isomer: 7.73 (dd, 4H), 4.42 (q, 4H), 1.42 (t, 6H). minor isomer: 7.55 (dd, 4H), 4.35 (q, 4H), 1.37 (t, 6H). **^{13}C NMR** ($CDCl_3$, 126 MHz): δ 163.7, 156.0, 154.0, 133.7, 114.0, 62.2, 14.2. **HRMS** (ESI +): 399.0926 $(M+H)^+$, 416.1229 $(M+NH_4)^+$, 421.0759 $(M+Na)^+$

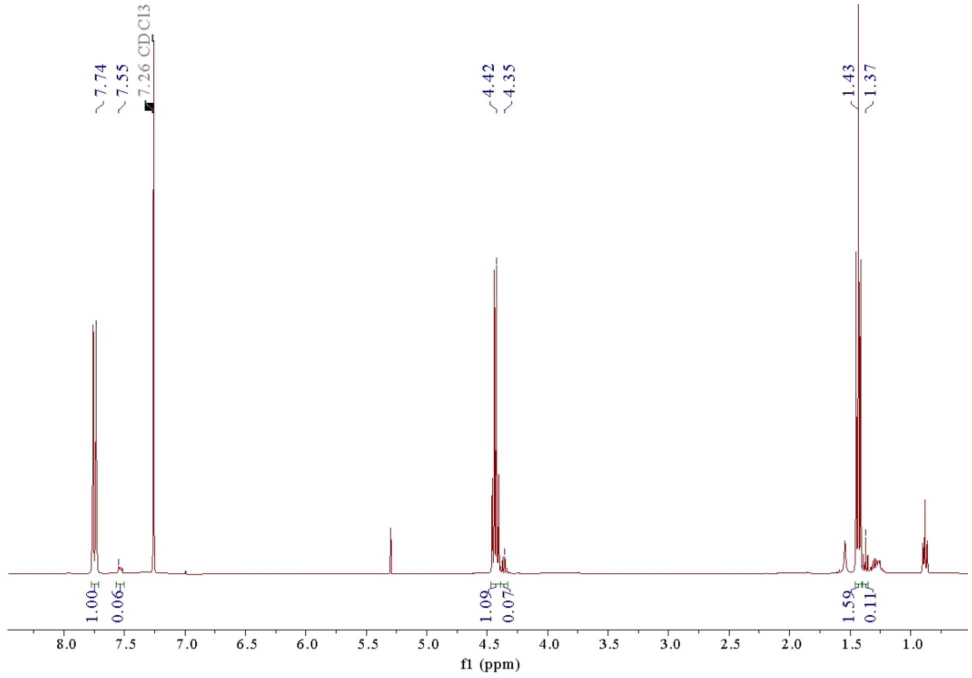

Figure S1. ^{1}H NMR of diethyl 4,4'-(diazene-1,2-diyl)€-bis(3,5-difluorobenzoate)

Synthesis of 4,4'-(diazene-1,2-diyl)bis(3,5-difluorobenzoic acid)[2]

Diethyl 4,4'-(diazene-1,2-diyl)bis(3,5-difluorobenzoate) (0.4994 g, 1.25 mmol) was dissolved in THF (25 mL). In a separate flask, KOH (0.32 g, 17.77 mmol) was dissolved in H_2O (13 mL). The solutions were combined and stirred for 1 h. After this time, the reaction was cooled to room temperature. Next, 1.0 M HCl (20 mL) was added to precipitate out a solid. The light orange solid collected via centrifugation and washed with methanol. (0.42 g, 98% yield)

Isomeric ratio: 91:9 **^{1}H NMR** (d_6-DMSO, 400 MHz): δ major isomer: 13.9 (br s, 2H), 7.78 (dd, 4H) **^{13}C NMR** ($CDCl_3$, 126 MHz): δ 163.7, 156.0, 154.0, 133.7, 114.0, 62.2, 14.2. **^{19}F NMR** (d_6-DMSO, 400 MHz): δ -120.08

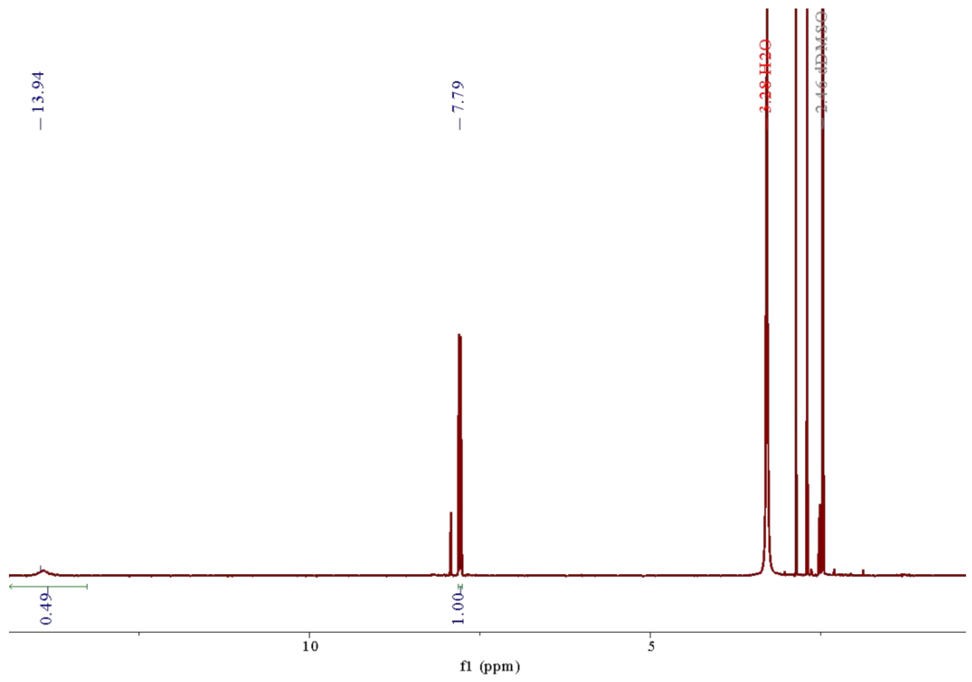

Figure S2. ^{1}H NMR of 4,4'-(diazene-1,2-diyl)bis(3,5-difluorobenzoic acid) (peaks @ 7.95, 2.89, and 2.73 ppm correspond to residual DMF solvent from recrystallization)

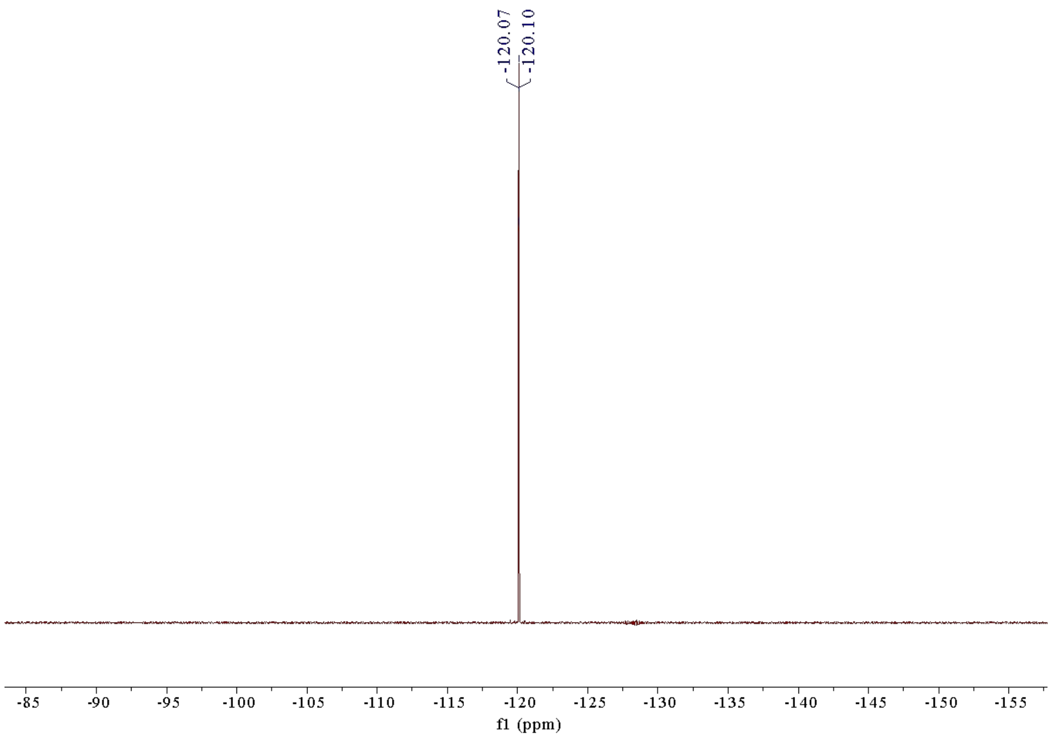

Figure S3. ^{19}F NMR of 4,4'-(diazene-1,2-diyl)bis(3,5-difluorobenzoic acid)

IV. MTV MOF Synthesis and Characterization

General Synthetic Procedure for the Preparation of MTV MOFs

In a typical synthesis, $ZrCl_4$ (0.1 mmol), DMF (1.5 mL), and formic acid (75 µL) were combined in a 6 dram vial and sonicated for 5 min. Varying ratios of 4,4'-(diazene-1,2-diyl)bis(3,5-difluorobenzoic acid) or 4,4′-azobenzenedicarboxylic acid linker (0.1 mmol total) were added, followed by H_2O (75 µL), and the mixture was sonicated again. The vial was stirred for 1-2 min to ensure the starting materials are fully mixed, then placed in a 120 °C oven for 15 min. Next, the vials were cooled to RT, collected via centrifugation (5 min), and washed with DMF (1x) and acetone (5x). To remove residual DMF in the pores, the particles were solvent exchanged in MeOH (12 h) and acetone (12 h) before further analysis. After solvent exchange, the particles were collected via centrifugation and dried under vacuum for 1 day at 60 °C.

UiO-AZB-F Synthesis

In a typical synthesis, $ZrCl_4$ (23.7 mg) and DMF (1.5 mL) are combined in a 6 dram vial. Next, formic acid (75 µL) is added, and the mixture is sonicated for 5 min. 4,4'-(diazene-1,2-diyl)bis(3,5-difluorobenzoic acid) (34.9 mg) is added, followed by H_2O (75 uL), and the mixture is sonicated again. The vial is placed on a stir plate for 1-2 min to ensure the starting materials are fully mixed, then placed in a 120 °C oven for 15 min. Next, the vials were cooled to RT and the contents were transferred to 1.5 mL centrifuge tubes. The particles were collected via centrifugation (5 min). The particles were subsequently washed with DMF (1x) and acetone (5x). To remove residual DMF in the pores, the particles were solvent exchanged in MeOH (12 h) and acetone (12 h) before further analysis. After solvent exchange, the particles were collected via centrifugation and dried under vacuum for 1 day at 60 °C.

MTV MOF (80% F) Synthesis

In a typical synthesis, $ZrCl_4$ (23.7 mg) and DMF (1.5 mL) are combined in a 6 dram vial. Next, formic acid (75.5 uL) is added, and the mixture is sonicated for 5 min. 4,4'-(diazene-1,2-

diyl)bis(3,5-difluorobenzoic acid) (27.3 mg) and 4,4′-azobenzenedicarboxylic acid (8.2 mg) are added, followed by H_2O (75 uL), and the mixture is sonicated again. The vial is placed on a stir plate for 1-2 min to ensure the starting materials are fully mixed, then placed in a 120 °C oven for 15 min. Next, the vials were cooled to RT and the contents were transferred to 1.5 mL centrifuge tubes. The particles were collected via centrifugation (5 min). The particles were subsequently washed with DMF (1x) and acetone (5x). The particles were collected via centrifugation and dried under vacuum for 1 day at 60 °C.

MTV MOF (50% F) Synthesis

In a typical synthesis, $ZrCl_4$ (23.7 mg) and DMF (1.5 mL) are combined in a 6 dram vial. Next, formic acid (75.5 uL) is added, and the mixture is sonicated for 5 min. 4,4'-(diazene-1,2-diyl)bis(3,5-difluorobenzoic acid) (17.5 mg) and 4,4′-azobenzenedicarboxylic acid (14.1 mg) are added, followed by H_2O (75 uL), and the mixture is sonicated again. The vial is placed on a stir plate for 1-2 min to ensure the starting materials are fully mixed, then placed in a 120 °C oven for 15 min. Next, the vials were cooled to RT and the contents were transferred to 1.5 mL centrifuge tubes. The particles were collected via centrifugation (5 min). The particles were subsequently washed with DMF (1x) and acetone (5x). The particles were collected via centrifugation and dried under vacuum for 1 day at 60 °C.

MTV MOF (30% F) Synthesis

In a typical synthesis, $ZrCl_4$ (23.7 mg) and DMF (1.5 mL) are combined in a 6 dram vial. Next, formic acid (75.5 uL) is added, and the mixture is sonicated for 5 min. 4,4'-(diazene-1,2-diyl)bis(3,5-difluorobenzoic acid) (10 mg) and 4,4′-azobenzenedicarboxylic acid (19.3 mg) are added, followed by H_2O (75 uL), and the mixture is sonicated again. The vial is placed on a stir plate for 1-2 min to ensure the starting materials are fully mixed, then placed in a 120 °C oven for 15 min. Next, the vials were cooled to RT and the contents were transferred to 1.5 mL centrifuge tubes. The particles were collected via centrifugation (5 min). The particles were subsequently washed with DMF (1x) and acetone (5x). The particles were collected via centrifugation and dried under vacuum for 1 day at 60 °C.

MTV MOF (15% F) Synthesis

In a typical synthesis, $ZrCl_4$ (23.7 mg) and DMF (1.5 mL) are combined in a 6 dram vial. Next, formic acid (75.5 uL) is added, and the mixture is sonicated for 5 min. 4,4'-(diazene-1,2-diyl)bis(3,5-difluorobenzoic acid) (5.1 mg) and 4,4′-azobenzenedicarboxylic acid (23 mg) are added, followed by H_2O (75 uL), and the mixture is sonicated again. The vial is placed on a stir plate for 1-2 min to ensure the starting materials are fully mixed, then placed in a 120 °C oven for 15 min. Next, the vials were cooled to RT and the contents were transferred to 1.5 mL centrifuge tubes. The particles were collected via centrifugation (5 min). The particles were subsequently washed with DMF (1x) and acetone (5x). The particles were collected via centrifugation and dried under vacuum for 1 day at 60 °C.

UiO-AZB Synthesis

In a typical synthesis, $ZrCl_4$ (23.7 mg) and DMF (1.5 mL) are combined in a 6 dram vial. Next, formic acid (75.5 uL) is added, and the mixture is sonicated for 5 min. 4,4′-azobenzenedicarboxylic acid (27 mg) was added, followed by H_2O (75 uL), and the mixture is sonicated again. The vial is placed on a stir plate for 1-2 min to ensure the starting materials are fully mixed, then placed in a 120 °C oven for 120 min. Next, the vials were cooled to RT and the contents were transferred to 1.5 mL centrifuge tubes. The particles were collected via centrifugation (5 min). The particles were subsequently washed with DMF (1x) and acetone (5x). The particles were collected via centrifugation and dried under vacuum for 1 day at 60 °C.

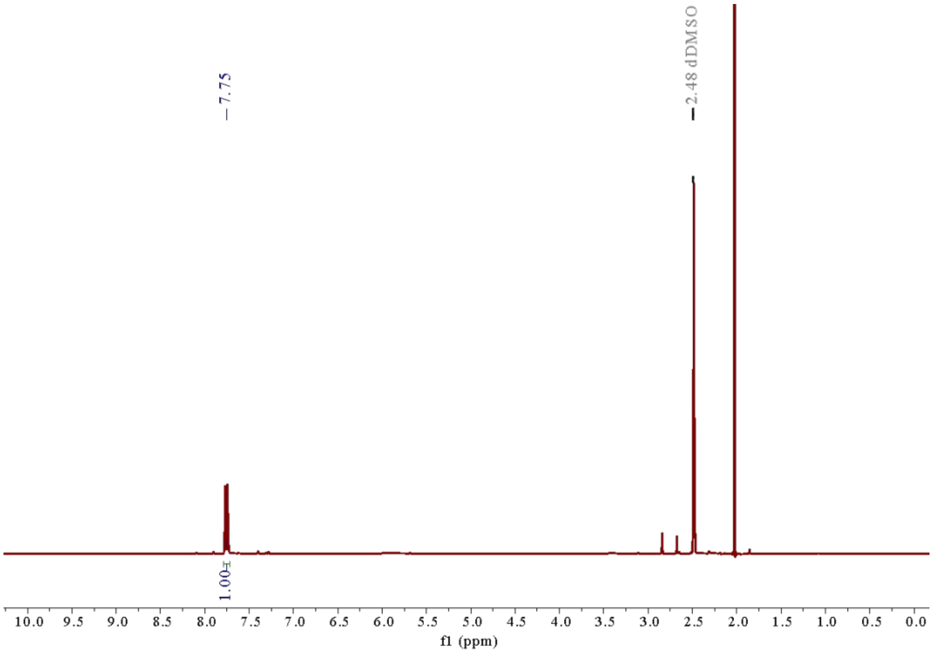

Figure S4 Sample ^{1}H NMR spectrum for digested UiO-AZB-F MOF sample

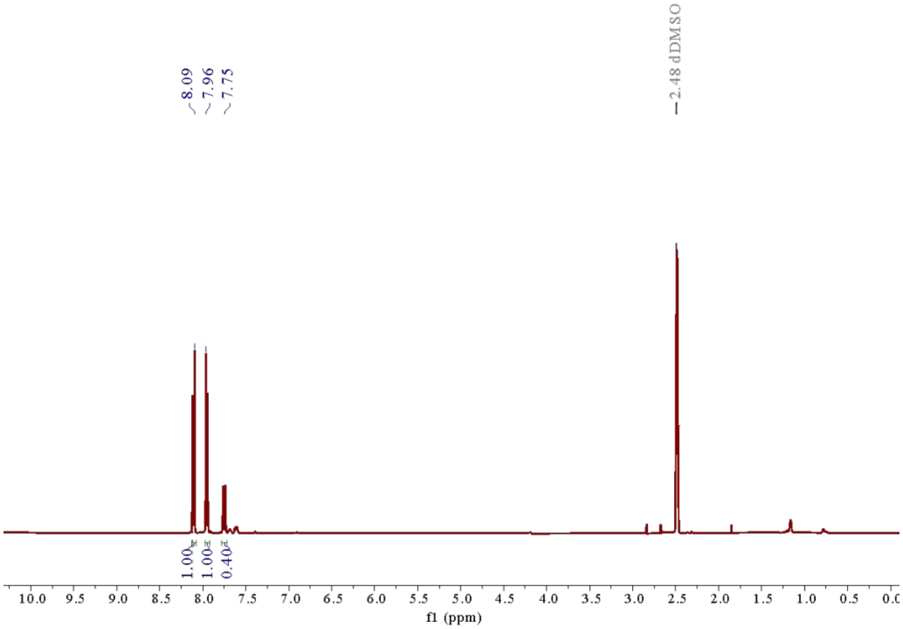

Figure S5 Sample ^{1}H NMR spectrum for digested 30% F MOF sample

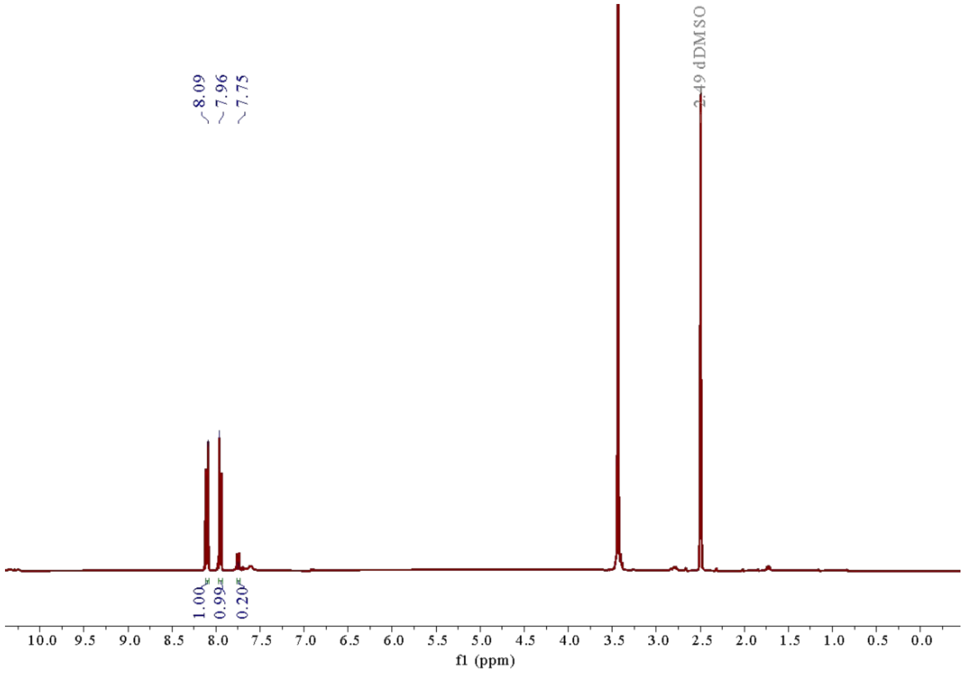

Figure S6 Sample ^{1}H NMR spectrum for digested 15% F MOF sample

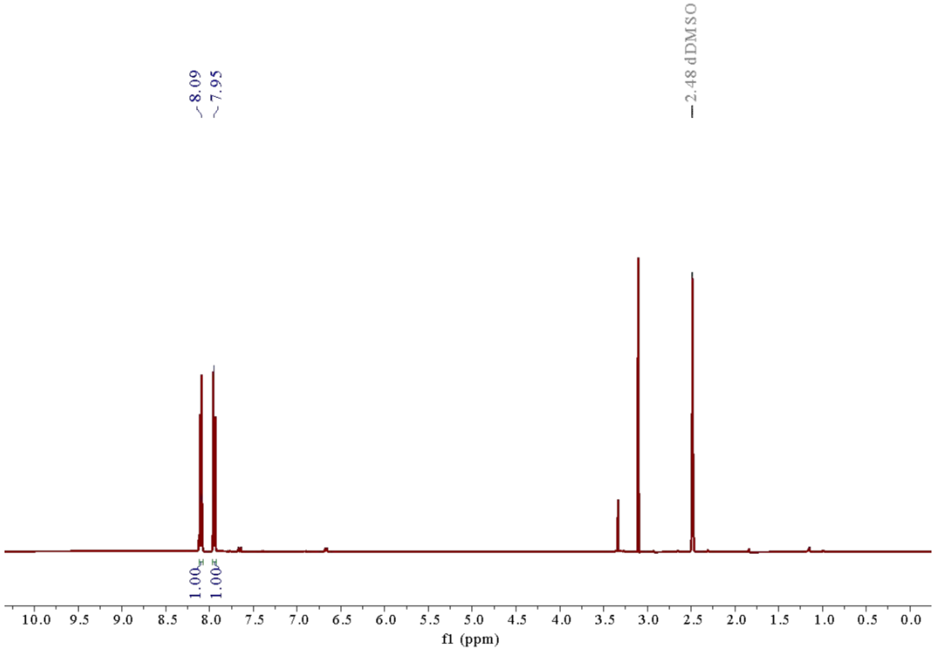

Figure S7 Sample ^{1}H NMR spectrum for digested UiO-AZB MOF sample

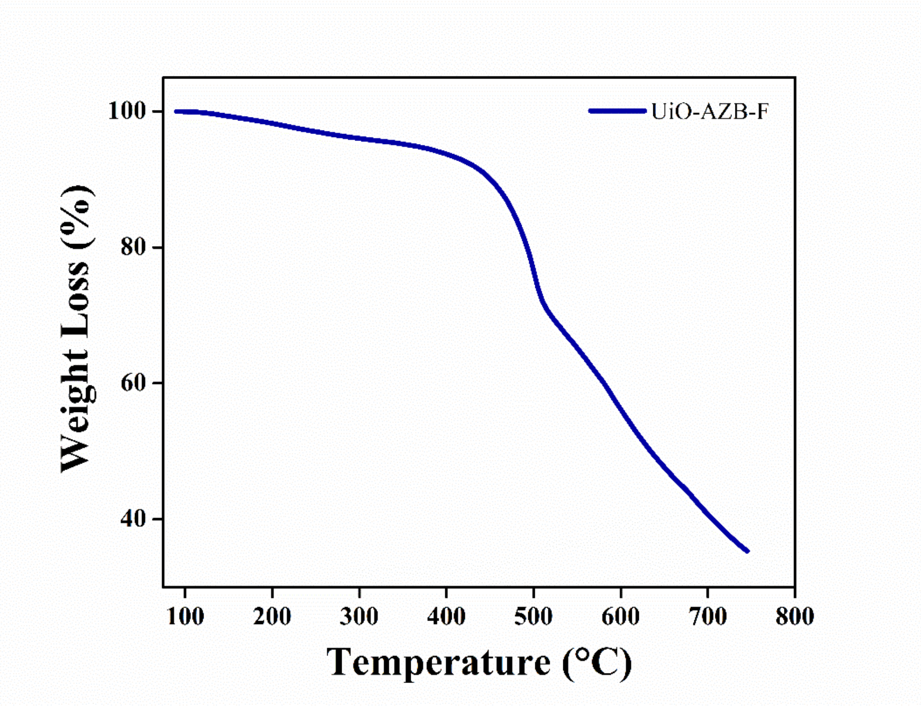

Figure S8 Thermogravimetric analysis (TGA) of UiO-AZB-F MOF under N_2. The initial weight loss between 100–200 °C is attributed to loss of residual modulator (formic acid) and the second weight loss occurring from 450 °C–700 °C corresponds to degradation of the linker/framework.

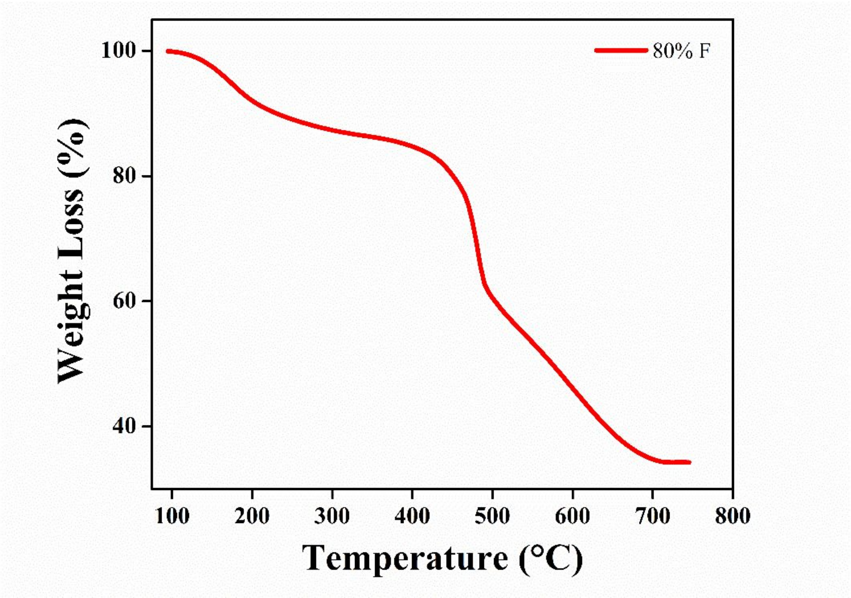

Figure S9 Thermogravimetric analysis (TGA) of 80% F MOF under N_2. The initial weight loss between 100–200 °C is attributed to loss of residual modulator (formic acid) and the second weight loss occurring from 450 °C–700 °C corresponds to degradation of the linker/framework.

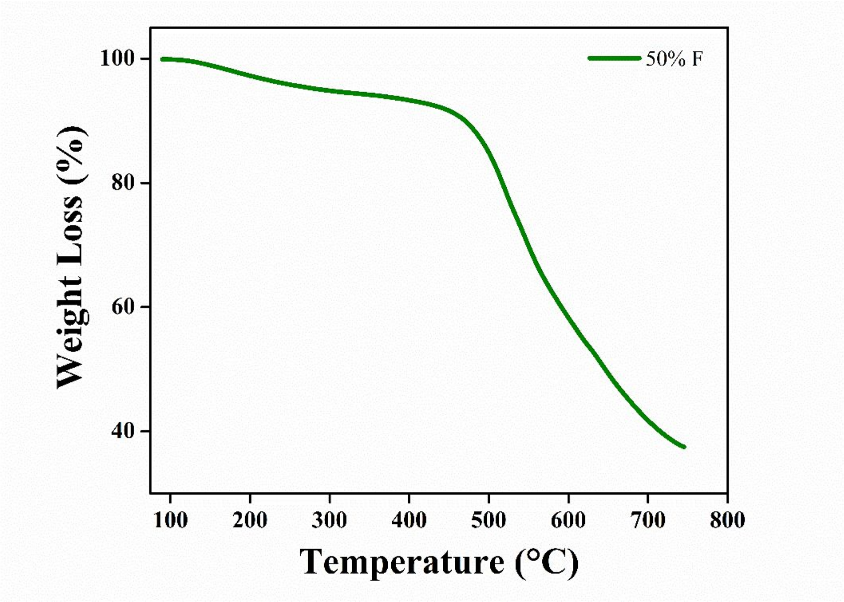

Figure S10 Thermogravimetric analysis (TGA) of 50% F MOF under N_2. The initial weight loss between 100–200 °C is attributed to loss of residual modulator (formic acid) and the second weight loss occurring from 450 °C–700 °C corresponds to degradation of the linker/framework.

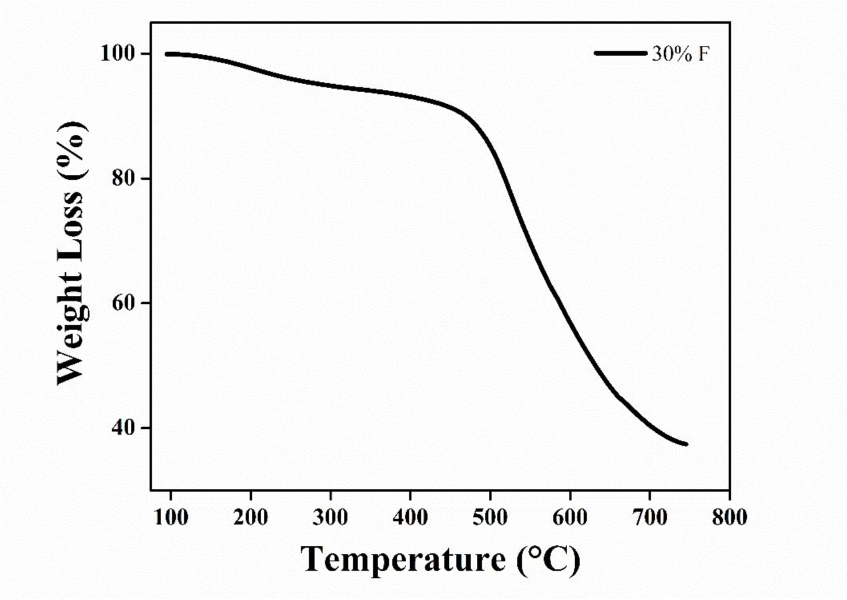

Figure S11 Thermogravimetric analysis (TGA) of 30% F MOF under N_2. The initial weight loss between 100–200 °C is attributed to loss of residual modulator (formic acid) and the second weight loss occurring from 450 °C–700 °C corresponds to degradation of the linker/framework.

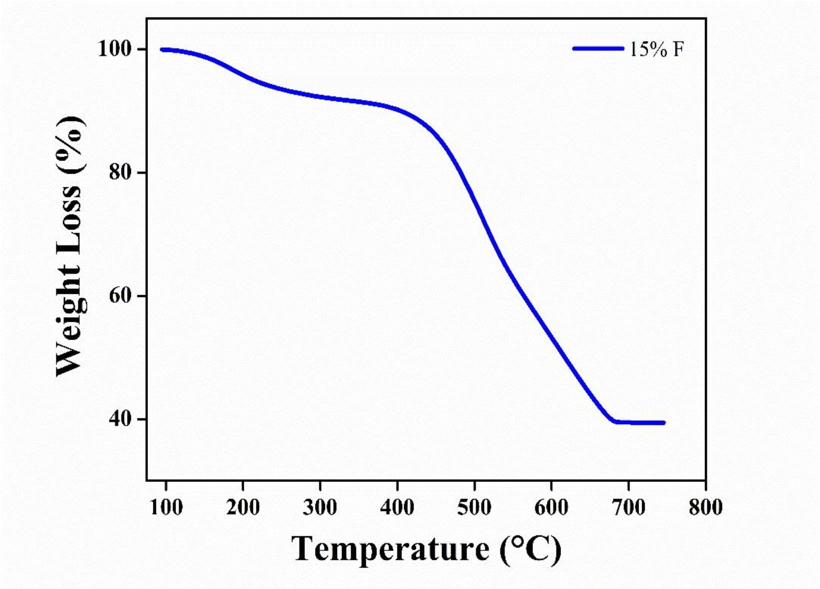

Figure S12 Thermogravimetric analysis (TGA) of 15% F MOF under N_2. The initial weight loss between 100–200 °C is attributed to loss of residual modulator (formic acid) and the second weight loss occurring from 450 °C–700 °C corresponds to degradation of the linker/framework.

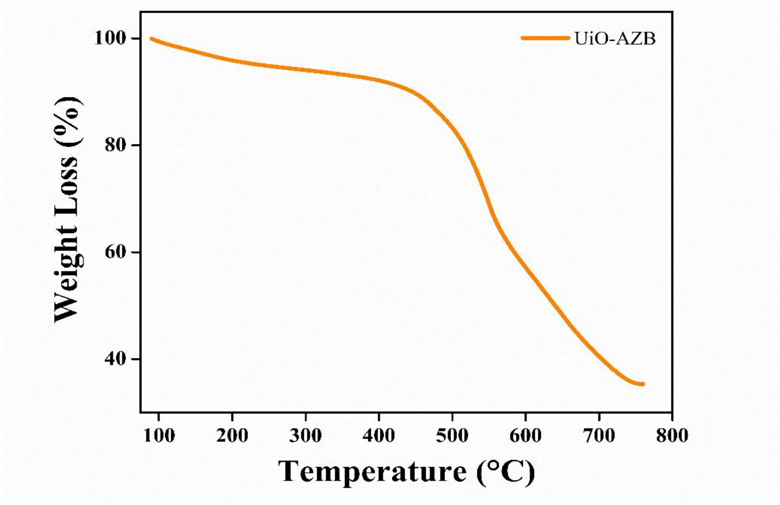

Figure S13 Thermogravimetric analysis (TGA) of UiO-AZB MOF under N_2. The initial weight loss between 100–200 °C is attributed to loss of residual modulator (formic acid) and the second weight loss occurring from 450 °C–700 °C corresponds to degradation of the linker/framework.

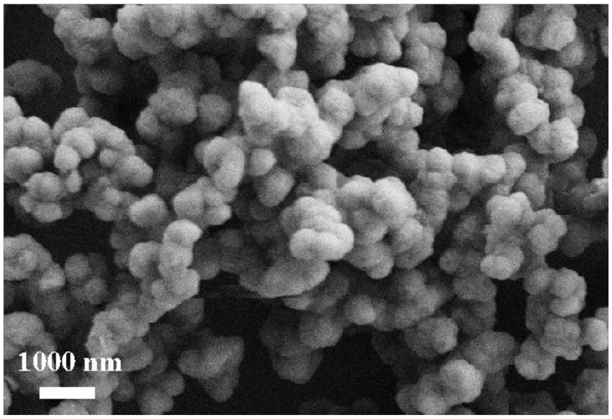

Figure S14 SEM Image of UiO-AZB-F particles

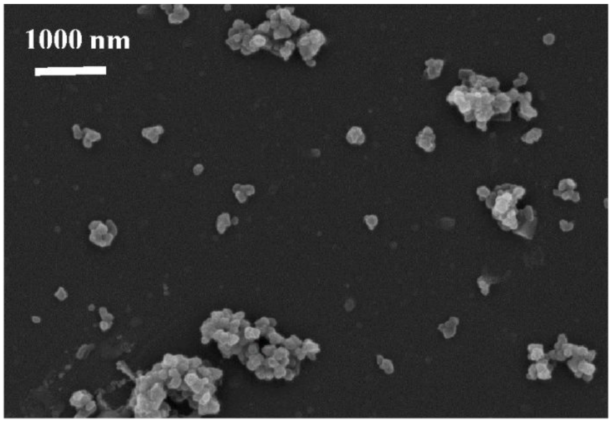

Figure S15 SEM Image of 80% F particles

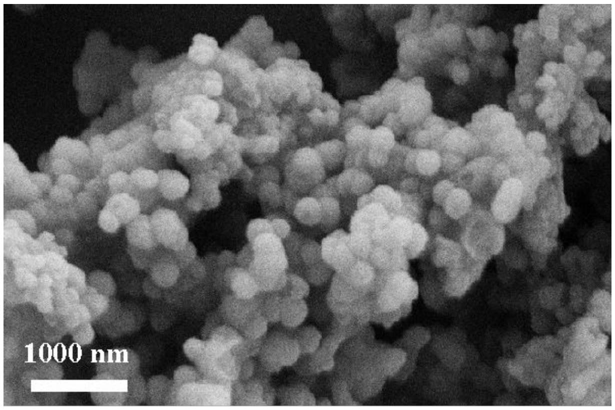

Figure S16 SEM Image of 50% F particles

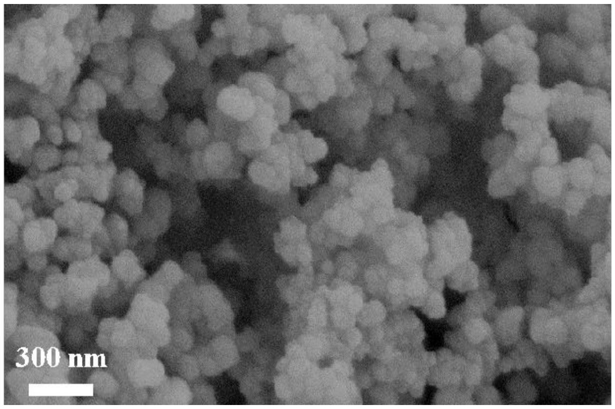

Figure S17 SEM Image of 30% F particles

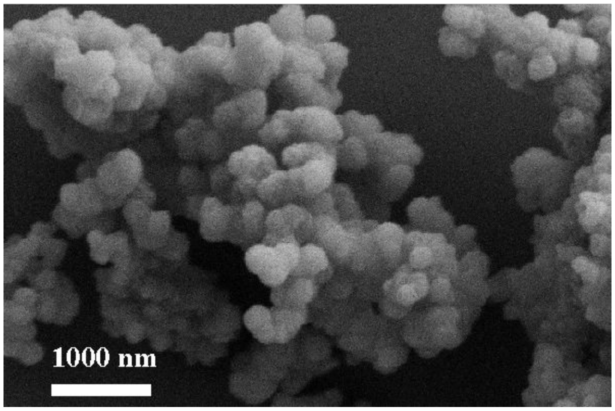

Figure S18 SEM Image of UiO-AZB particles

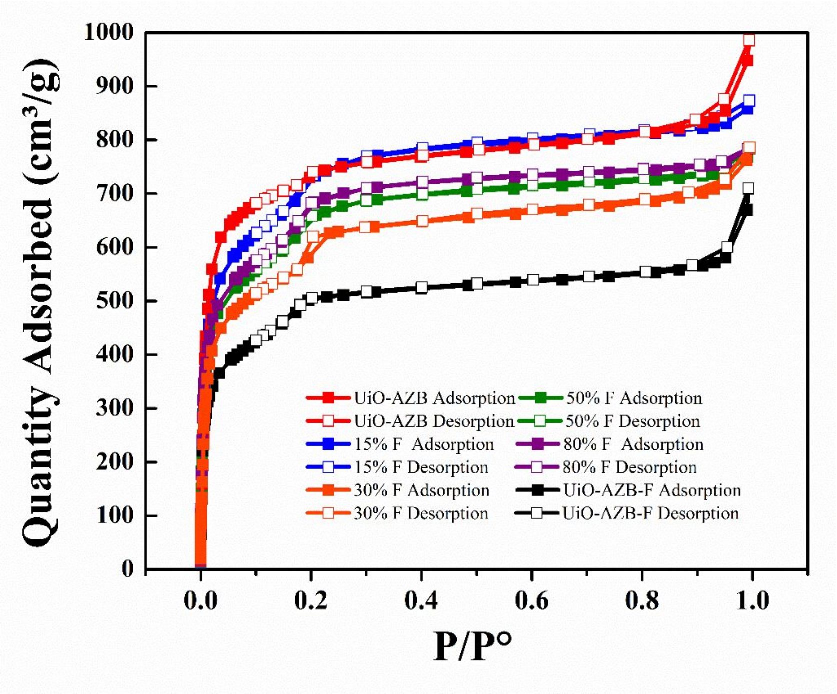

Figure S19 BET Data of MTV Derivatives

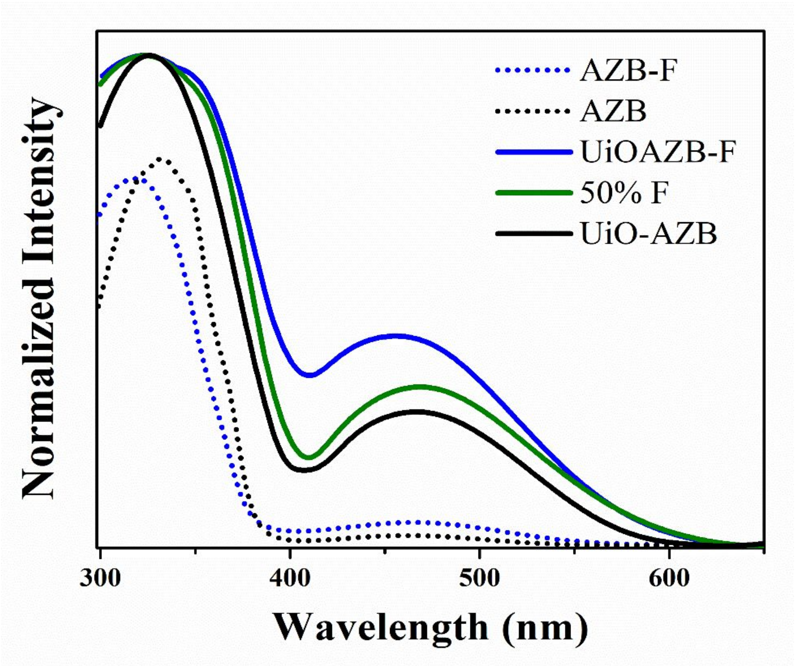

Figure S20 Diffuse reflectance of UiOAZB-F (blue), 50% F (green), and UiO-AZB (black). The solution state UVVis profiles of respective linkers (AZB and AZB-F) are also shown (dotted).

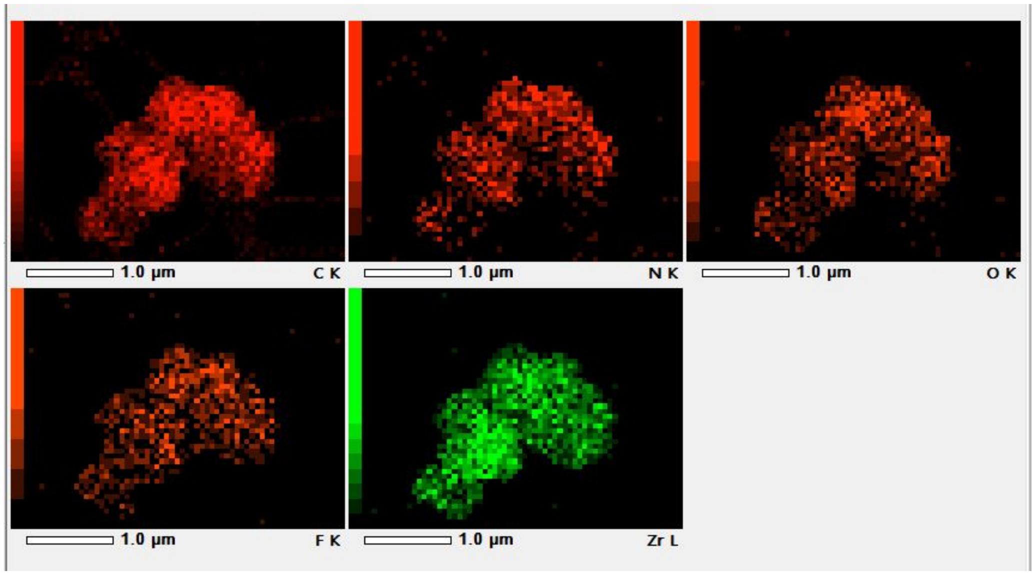

Figure S21 EDS mapping of 100% F Sample

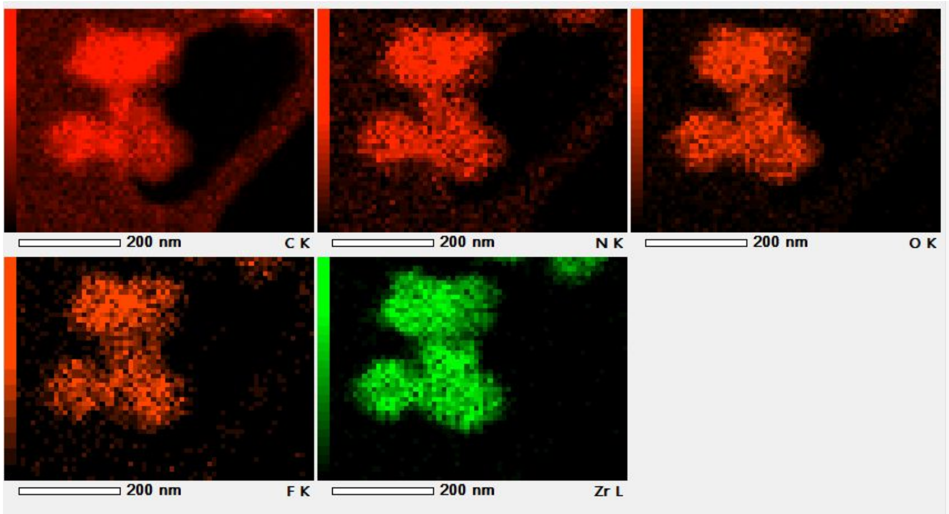

Figure S22 EDS mapping of 15% F Sample

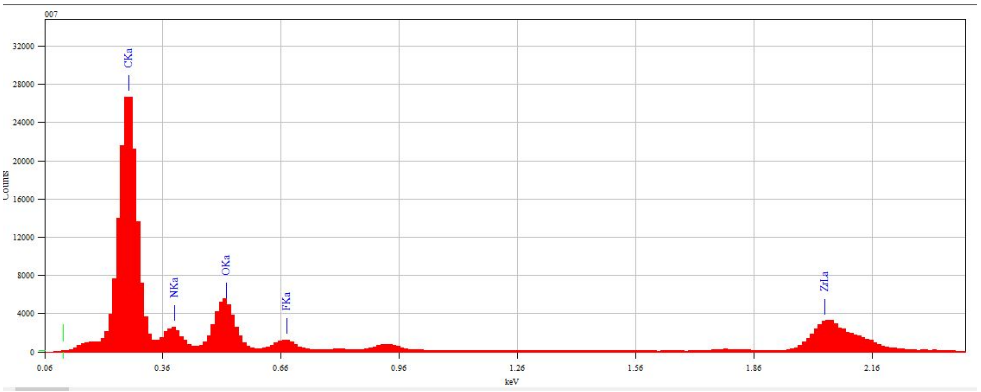

Figure S23 EDS Spot analysis of 15% F Sample

V. Drug Loading (5-FU & Nile-red) and Release studies

5-fluorouracil Adsorption in MTV MOFs

5-fluorouracil was loaded into the frameworks using an *in situ* loading procedure. In a typical synthesis, $ZrCl_4$ (0.1 mmol), DMF (1.5 mL), and formic acid (75 µL) were combined in a 6 dram vial and sonicated for 5 min. Varying ratios of 4,4'-(diazene-1,2-diyl)bis(3,5-difluorobenzoic acid) or 4,4′-azobenzenedicarboxylic acid linker (0.1 mmol total) were added, followed by H_2O (75 µL) and 5-fluorouracil (500 mg). The vial was heated at 120 °C while stirring on a hotplate for 15 min.

Next, the vials were cooled to RT, collected via centrifugation (5 min), and washed with DMF (1x) and acetone (5x).

Nile Red Adsorption in MTV MOFs

Nile Red was loaded into the frameworks using a *in situ* loading procedure. In a typical synthesis, $ZrCl_4$ (0.1 mmol), DMF (1.5 mL), and formic acid (75 μL) were combined in a 6 dram vial and sonicated for 5 min. Varying ratios of 4,4'-(diazene-1,2-diyl)bis(3,5-difluorobenzoic acid) or 4,4′-azobenzenedicarboxylic acid linker (0.1 mmol total) were added, followed by H_2O (75 μL) and Nile Red (35 mg). The vial was heated at 120 °C while stirring on a hotplate for 15 min. Next, the vials were cooled to RT, collected via centrifugation (5 min), and washed with DMF (1x) and acetone (5x). Detailed procedures and experimental conditions are available in the Supporting Information.

UiO-AZB Drug Loading (Post-synthetic)

In a flask, 400 mg of 5-fluorouracil was added to 120 mL EtOH. The mixture was stirred vigorously at 70 °C until the drug was fully dissolved (45 min). The solution was removed from heat. Next, UiO-AZB (100 mg) was added, and the flask was sonicated for 15 min to suspend the MOF particles. After sonication, the suspension was stirred at 60 °C for 5 days. The particles were collected via centrifugation, washed with EtOH to remove the surface-bound drug, and dried under vacuum for 1 day at 60 °C.

PEG-NH$_2$ Polymer Coating

Particles are resuspended in EtOH (5 mL), transferred to a vial, and sonicated. PEG-NH_2 (100 mg) is added to the solution, and the vial is sonicated until contents are fully suspended (~15 min). The solution was stirred at room temperature for 24 h in the dark. After, the particles were collected via centrifugation and washed with EtOH (x1). After washing, the particles were collected via centrifugation and dried under vacuum for 1 day at 60 °C.

5-Fluorouracil Release Studies

The experimental setup involves a small custom-made LED irradiation chamber. The box enables a standard well plate to be secured directly above an LED array containing four 20 mm diameter green LED chips/starboards (530 nm). The specifications of LEDS are as follows: 44 lumens/watt, 4.5V, max current of 14 A. The LEDs are seated symmetrically across the bottom of the irradiation chamber, ensuring homogeneity of illumination through the samples in the well plates above. To quantify 5-fluorouracil release via ^{19}F NMR, an internal standard was used. A stock solution of internal standard (2,6-difluorobenzoic acid, IS) and D_2O was prepared, and the pH was adjusted to 7.2. In a 24 well plate, between 2-4 mg of drug loaded material was added to each well. Next, 700 µL of IS solution was added to each well. The well plate was placed in an LED irradiation chamber and irradiated for 15, 30, 60, 90, or 120 min. After the designated time, the sample was filtered through a 0.45 µm syringe filter to remove MOF particles and transferred to an NMR tube for analysis. For the dark control experiments, sample preparation and workup were the same, but the well plate was wrapped in aluminum foil. (*Note*: the linker itself does not show up in these spectra because it is only soluble in aqueous solution at very acidic or basic pH).

The molar ratio of 5FU: IS was calculated according to the equation below:

$$\frac{\boldsymbol{mol\ 5FU}}{\boldsymbol{mol\ IS}} = \frac{Intergration\ of\ 5FU\ peak}{\#\ of\ F\ in\ 5FU} \times \frac{\#\ of\ F\ in\ IS}{Integration\ of\ IS\ peak}$$

To account for slight differences in initial sample mass/loading efficiency across trials, the percentage of release from the framework is calculated. The total amount of 5FU in the MOF is calculated by multiplying the mass of MOF added by the wt% loading (calculated previously). The absolute mass of 5FU released is estimated from ^{19}F NMR.

$$\%\ \boldsymbol{5FU\ Release} = \frac{Mass\ of\ 5FU\ (from\ 19F\ NMR)}{Total\ Mass\ of\ 5FU\ in\ MOF} \times 100$$

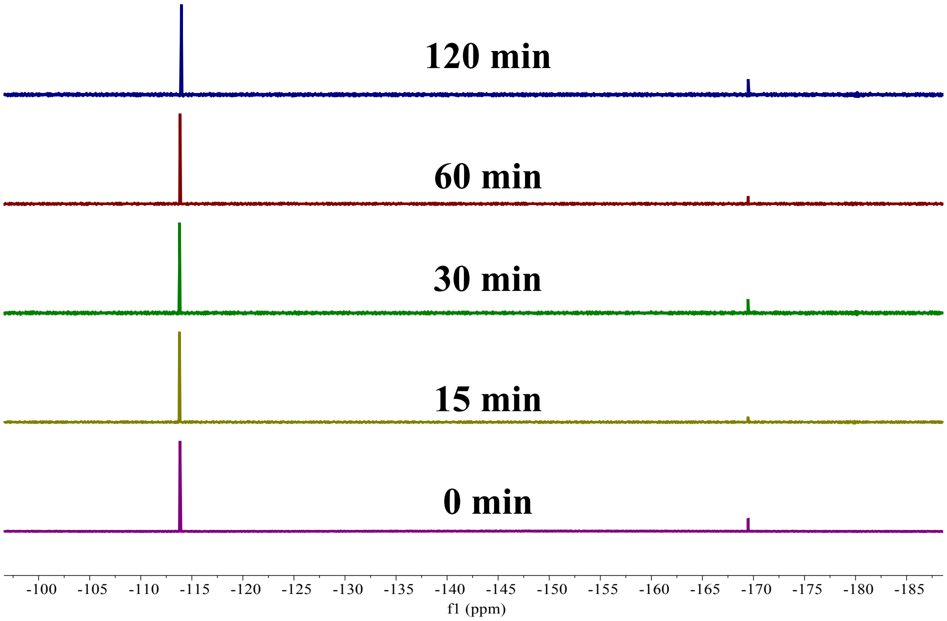

Figure S24. Quantitative ^{19}F NMR of 5-FU release from PEG-NH_2-UiO-AZB-F in the dark + heat. The signals for the internal standard 2,6-difluorobenzoic acid and 5-fluorouracil are visible at -114 and -169.5 ppm respectively. The intensity of the 5-FU peak remains the same throughout the time trial, indicating that the carrier shows no release after the original "burst".

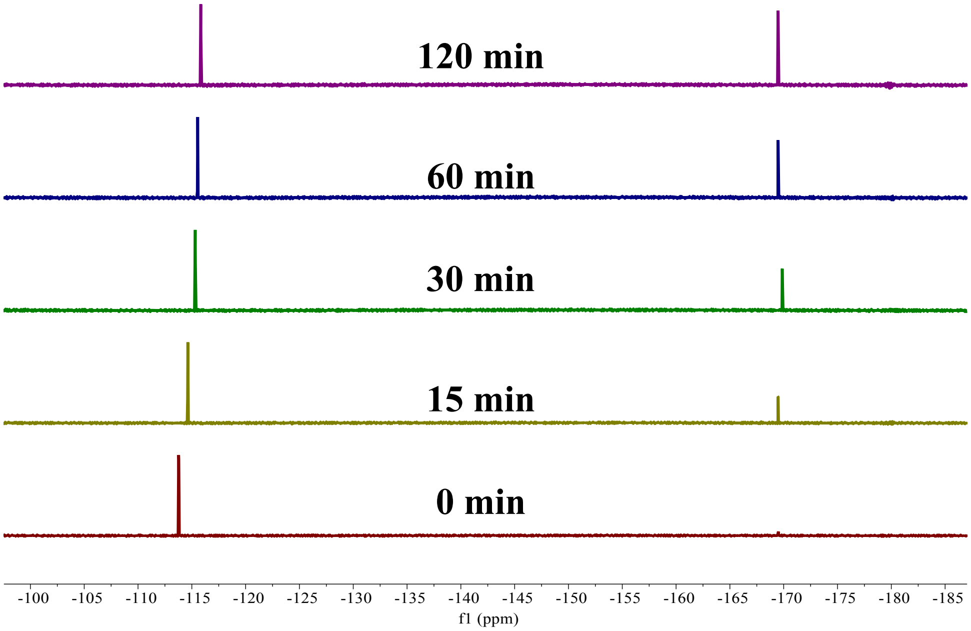

Figure S25. Quantitative ^{19}F NMR of 5-FU release from PEG-NH_2-UiO-AZB-F under green light irradiation. The signals for the internal standard 2,6-difluorobenzoic acid and 5-fluorouracil are visible at -

114 and -169.5 ppm, respectively. The position of the internal standard peak shifts slightly, likely due to the increasing amount of 5-fluorouracil present in solution.

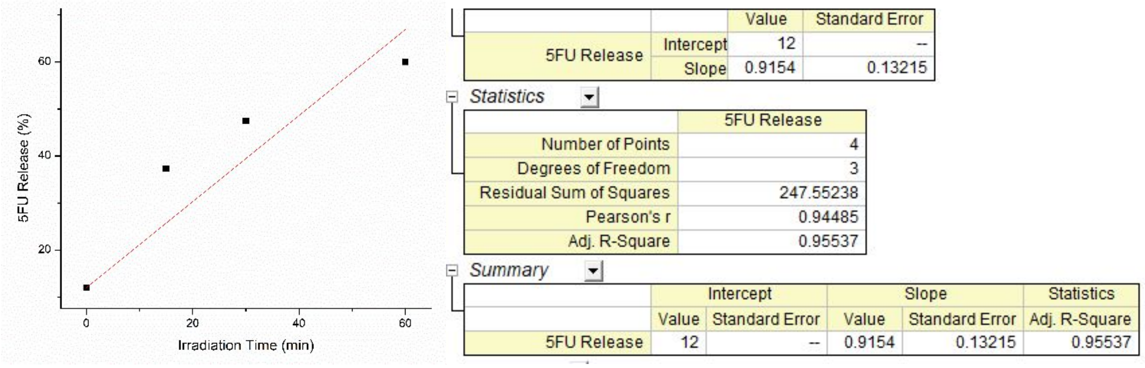

Figure S26. Rate analysis of 5FU release from PEGNH$_2$-UiO-AZB-F. A regression was fit to the linear portion of the release profile (time points 0–60 min).

Table S1 Characteristics of MTV Derivatives

**Sample**	**Average MW of MOF (g/mol)**	**Average # fluorinated linkers per node**
UiO-AZB	2289	0
15% F	2354	0.9
30% F	2418	1.8
50% F	2505	3
80% F	2634	4.8
UiO-AZB-F	2721	6

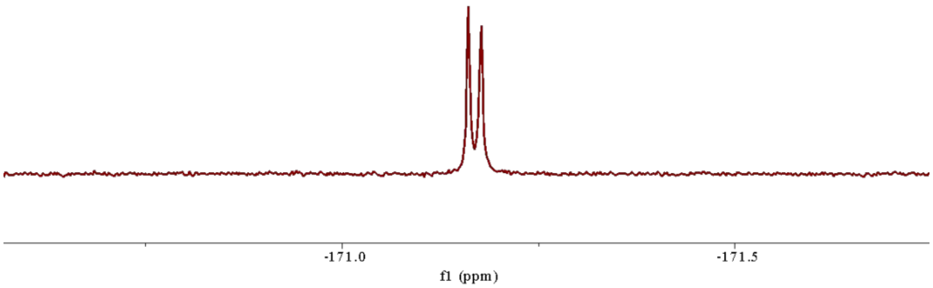

Figure S27 ^{19}F NMR spectrum of 5FU sample

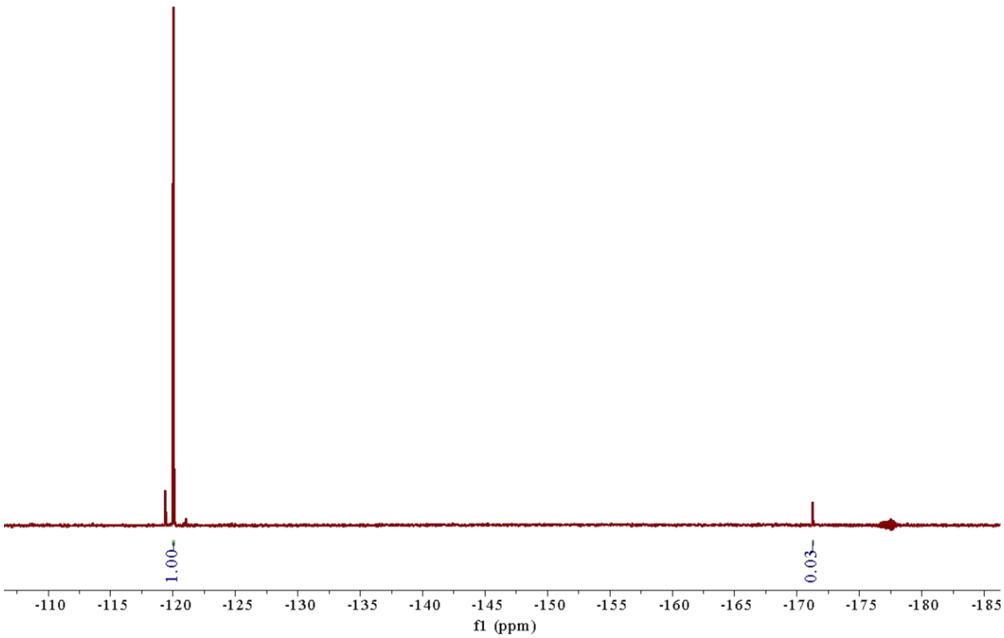

Figure S28 ^{19}F NMR spectrum of digested UiO-AZB- F + 5FU MOF sample

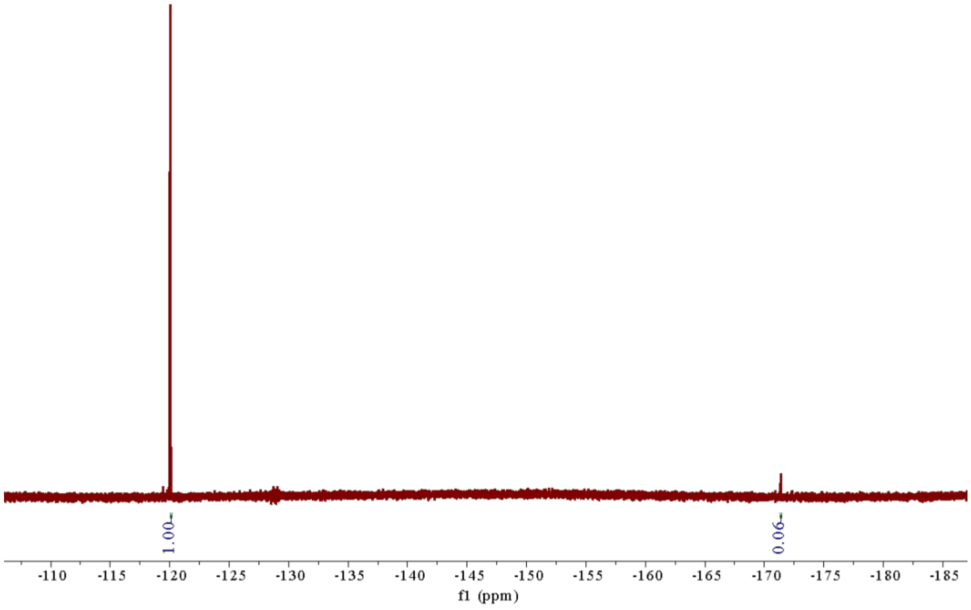

Figure S29 ^{19}F NMR spectrum of digested 80% F + 5FU MOF sample

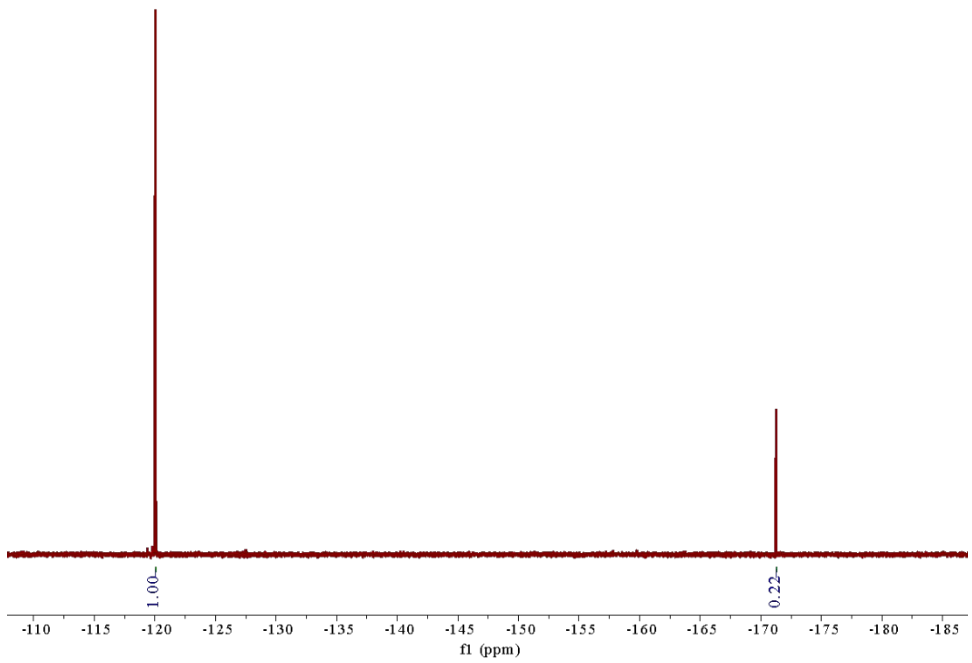

Figure S30 ^{19}F NMR spectrum of digested 50% F + 5FU MOF sample

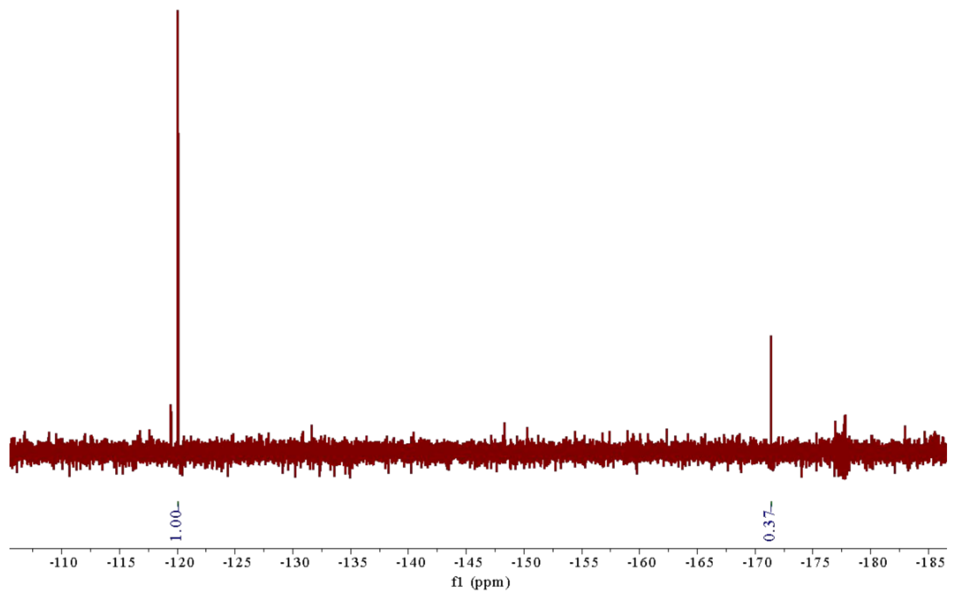

Figure S31 ^{19}F NMR spectrum of digested 30% F + 5FU MOF sample

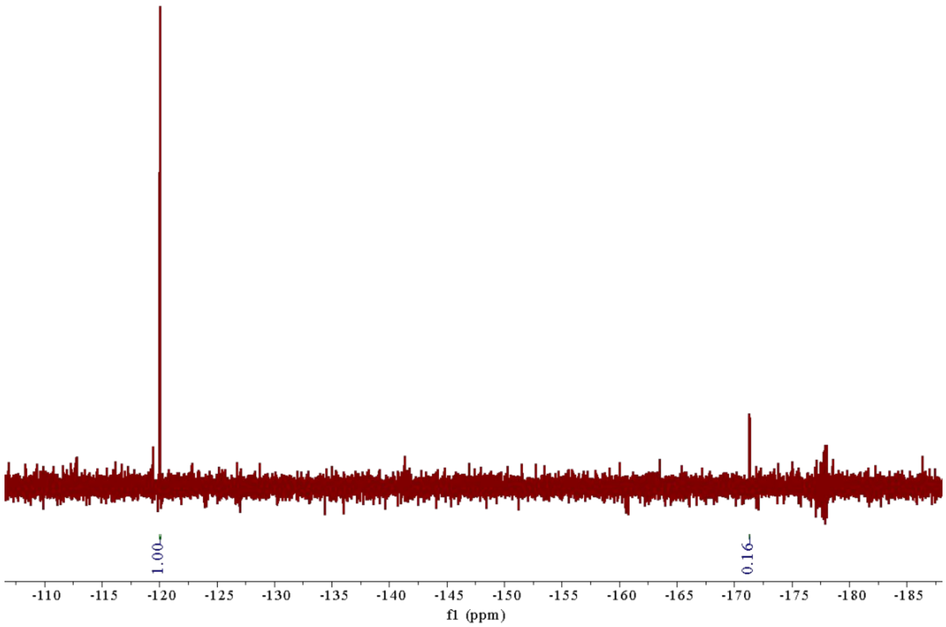

Figure S32 ^{19}F NMR spectrum of digested 15% F + 5FU MOF sample

Table S2 Summary of ^{19}F Data for 5-FU Quantification

**Sample**	**Average 5-FU Integration**	**Average Molar Ratio (5-FU: Linker)**	**Average Weight Percent (%)**
UiO-AZB	-	-	0
15% F	0.14	0.56	4
30% F	0.31	1.25	21
50% F	0.21	0.88	14
80% F	0.07	0.27	6
UiO-AZB-F	0.1	0.4	11

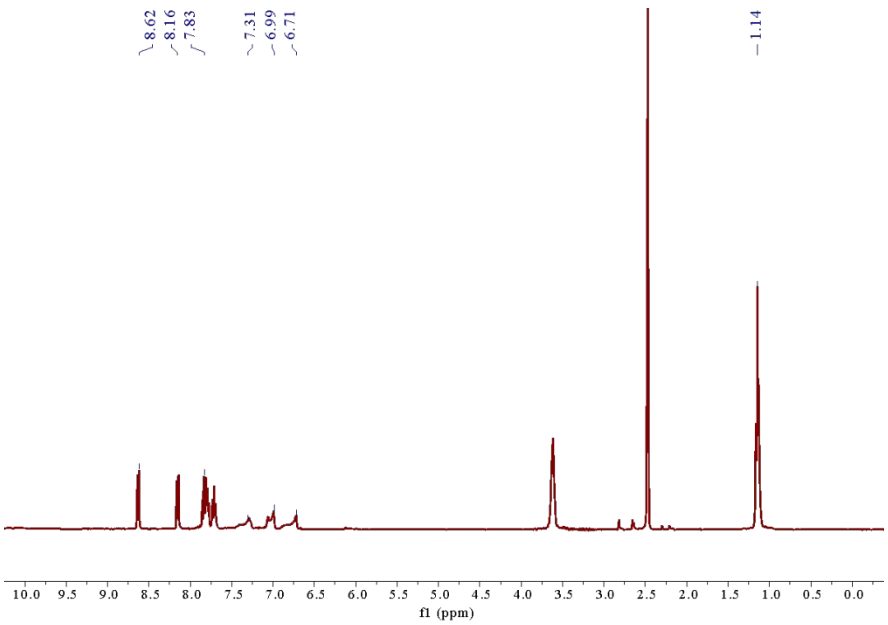

Figure S33 ^{1}H NMR spectrum of Nile Red in d_6DMSO + H_2SO_4.

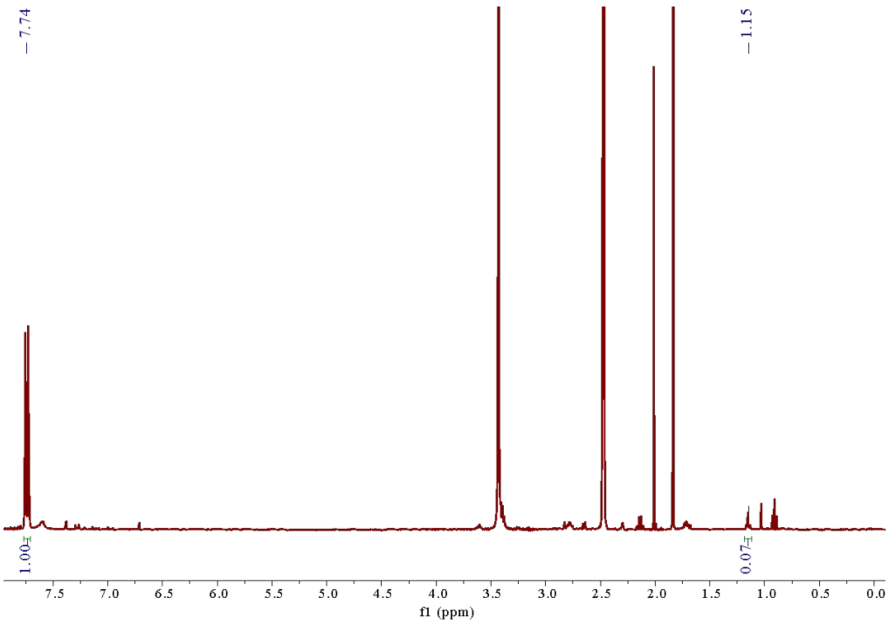

Figure S34 ^{1}H NMR spectrum of digested UiO-AZB-F + Nile Red MOF sample

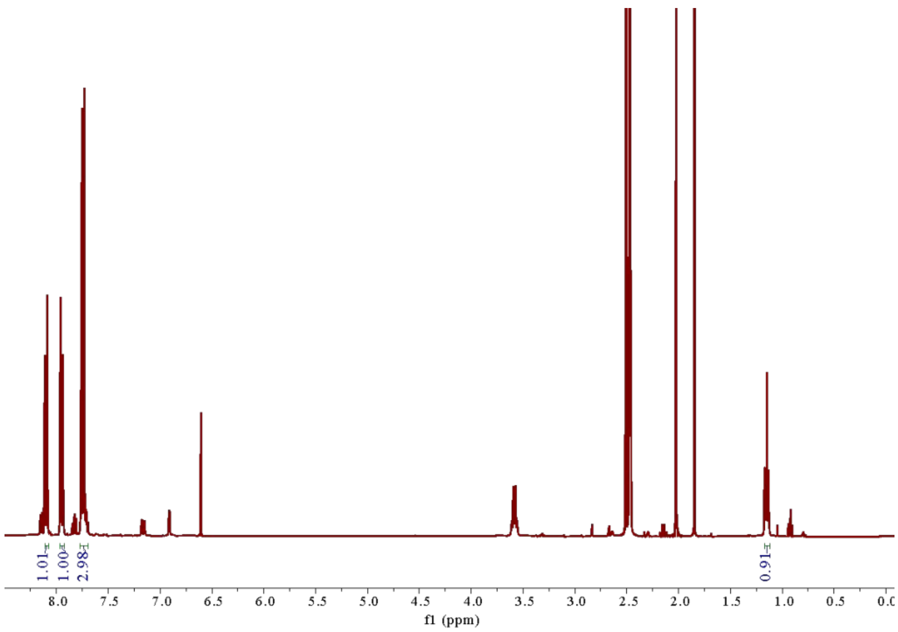

Figure S35 ^{1}H NMR spectrum of digested 80% F + Nile Red MOF sample

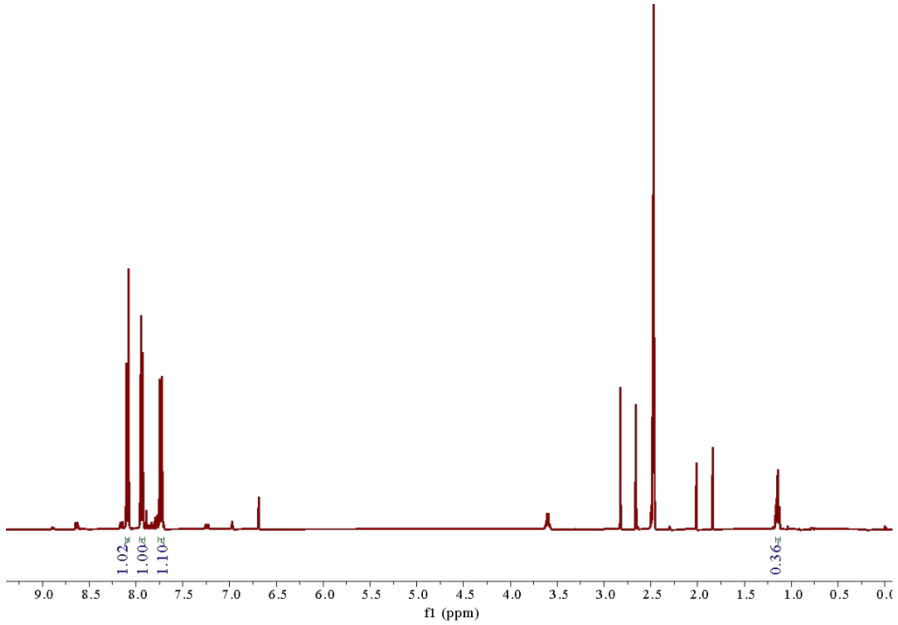

Figure S36 ^{1}H NMR spectrum of digested 50% F + Nile Red MOF sample

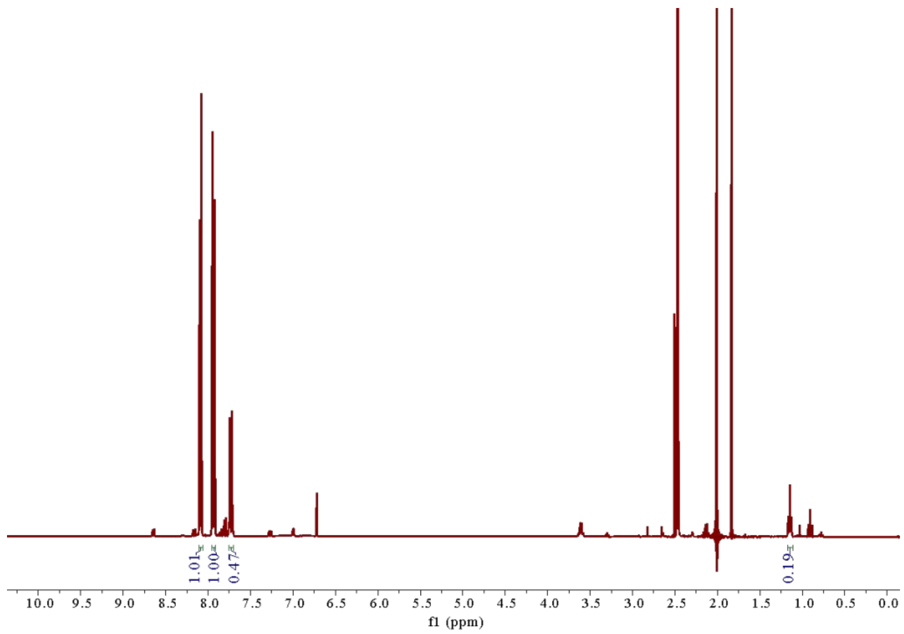

Figure S37 ^{1}H NMR spectrum of digested 30% F + Nile Red MOF sample

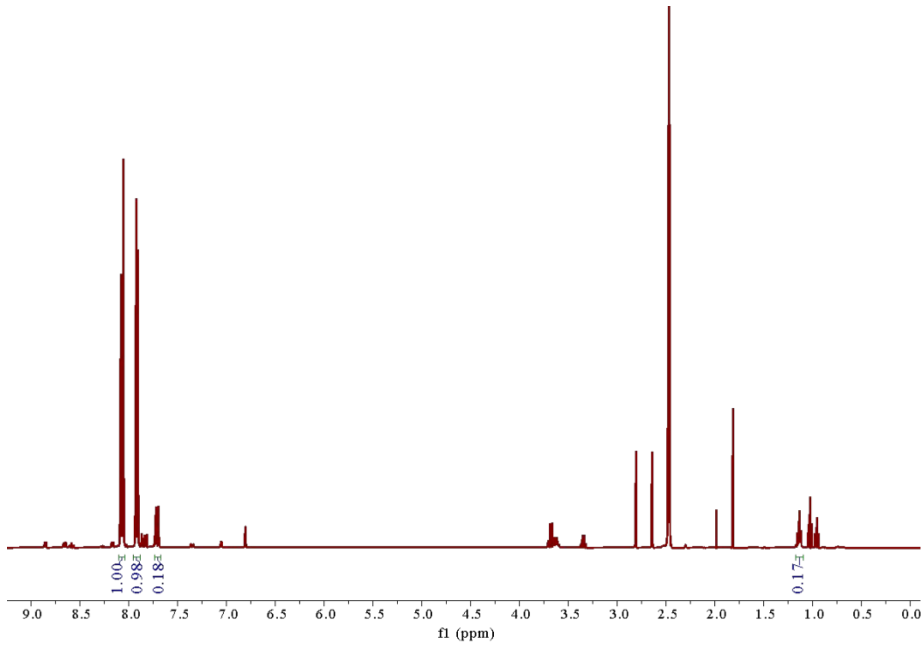

Figure S38 ^{1}H NMR spectrum of digested 15% F + Nile Red MOF sample

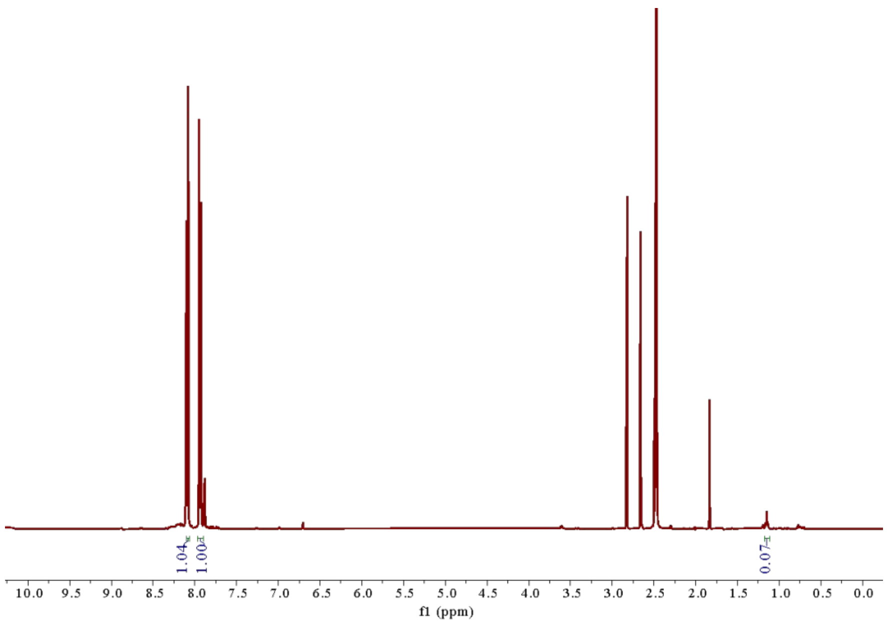

Figure S39 ^{1}H NMR spectrum of digested UiO-AZB + Nile Red MOF sample

Table S3 Summary of ^{1}H NMR Data for Nile Red Quantification

**Sample**	**Average Nile Red Integration**	**Average Molar Ratio (NR: Linker)**	**Average Weight Percent (%)**
UiO-AZB	0.08	0.05	4
15% F	0.13	0.08	6
30% F	0.16	0.11	6
50% F	0.28	0.19	7
80% F	0.7	0.47	10
UiO-AZB-F	0.07	0.05	3

Synthesis of PEGNH$_2$-coated MOFs

The amine group in PEG-NH$_2$ was chosen because it coordinates effectively with the unsaturated Zr^{4+} sites present in the UiO framework. This interaction is facilitated by the Lewis acid-base affinity between the Zr^{4+} sites and the lone pair of electrons on the nitrogen atom in the amine group. The process involves the diffusion of PEG-NH$_2$ into the MOF, where it anchors to the Zr^{4+} nodes, forming a stable coordination bond. This modification not only improves the stability of the MOF in aqueous environments but also provides a platform for further surface functionalization. For instance, the amine group on PEG allows for the conjugation of targeting ligands such as folic acid through well-established EDC/NHS coupling chemistry. Such

modifications can enhance the selective uptake of the material by cancer cells, broadening its potential in targeted drug delivery applications. For release studies, it was critical to functionalize the surface of MOF with polymer to enable suspension of particles in aqueous solutions. In a vial, 30 mg of 5-FU loaded MOF was suspended in 5 mL EtOH. The mixture was sonicated with a tip sonicator for 2 h, at which point 200 mg of bis(3-aminopropyl) terminated poly(ethylene glycol) was added to the solution. The mixture was sonicated for an additional hour and then stirred at room temperature overnight. The particles were collected via centrifugation, washed with EtOH (x2), and dried under vacuum for 1 day at 60 °C.

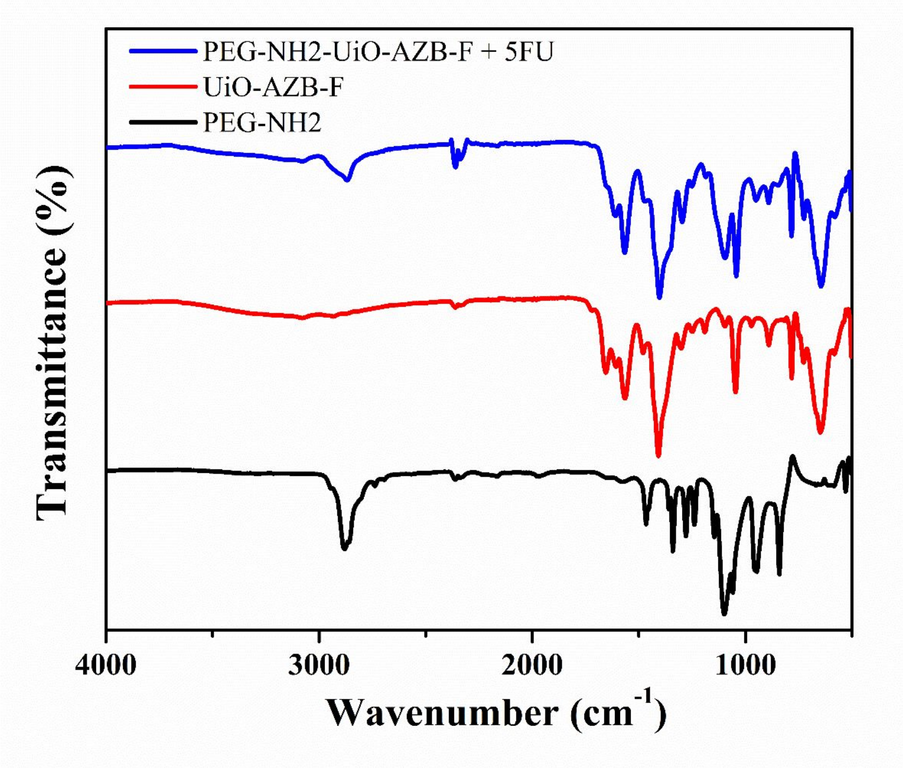

Figure S40 Infrared spectroscopy of $PEG-NH_2$ (black), UiO-AZB-F (red), and $PEGNH_2$-UiO-AZB-F+5FU (blue). The appearance of a broad peak at 2800 cm^{-1} in the blue spectrum represents the NH stretch of the polymer end groups. No amide stretches are observed, which indicates the polymer likely interacts with Zr^{4+} in the MOF framework rather than covalently binding to the framework.

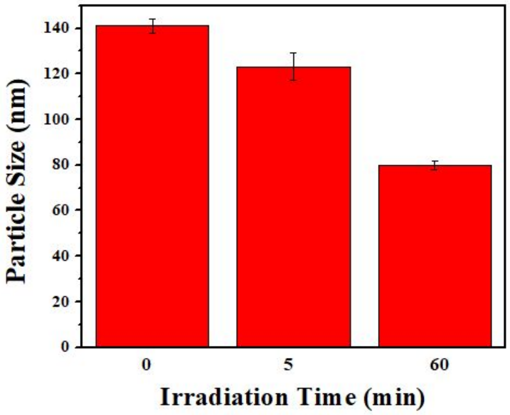

Figure S41 An irradiation time of 2 hours was chosen to ensure time for sufficient MOF degradation. Based on DLS, UiO-AZB-F photo exfoliates to about 50% of the original particle size in 1 h. For mixed linkers, the degradation is even slower due to a lower amount of green-light responsive linkers within the framework.

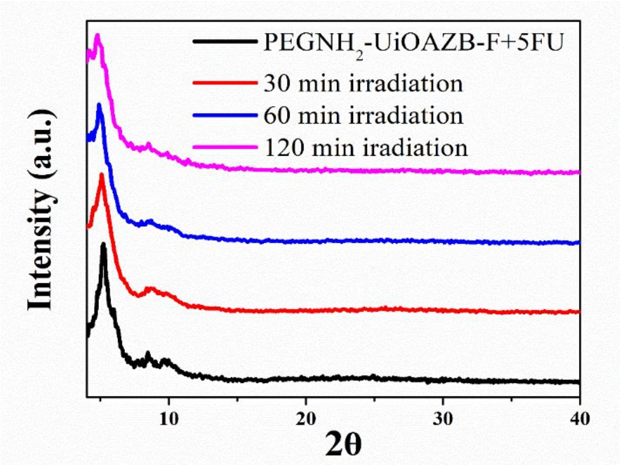

Figure S42: PXRD of UiO-AZB-F after photo exfoliates to about 50% of the original particle size in 1 h.

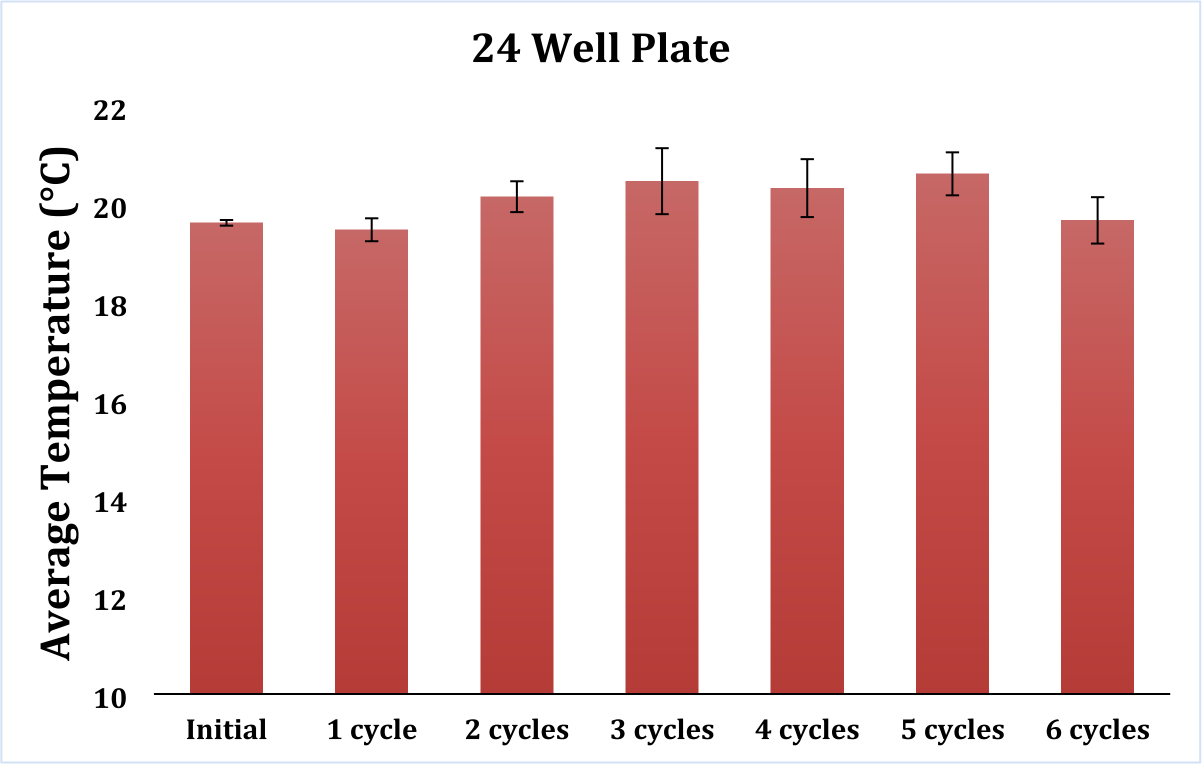

Figure S43 The temperature of a buffer media monitored in 15-minute intervals (1 cycle = 15 minutes) showing no change in temperature over time.

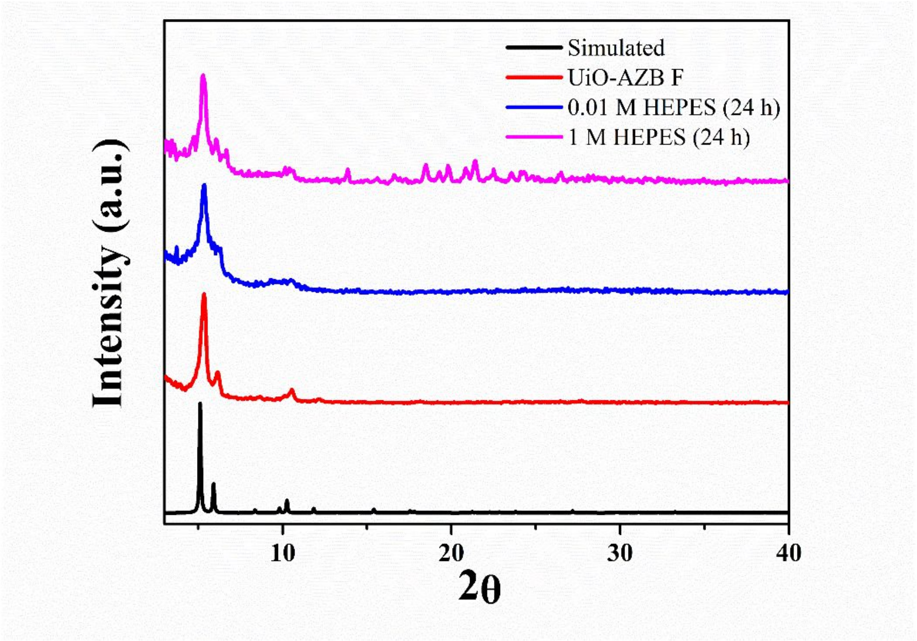

Figure S44 PXRD of UiO-AZB-F MOF in HEPES and DMEM buffer media.

Table S4 BET experimental results Comparison of all UiO-AZB and UiO-AZB-F MTV MOFs.

	BET SA (m^2/g)	BJH Average Pore Width (A)	Pore Volume (cm^3/g)
UiO-AZB-F	1320	21.56	0.33
UiO-AZB-F:H 2:1	2311	23.60	0.63
UiO-AZB-F:H 1:1	2267	25.36	0.64
UiO-AZB-F:H 1:2	2548	25.32	0.71
UiO-AZB	2774	42.80	0.65

S2. Simulations

I. Simulation Details

In order to investigate simulated drug adsorption in MOF, the multipurpose code RASPA was used to perform Grand Canonical Monte Carlo (GCMC) simulations. Similarly to experimental conditions, these simulations were conducted at 300K and 1 bar. Several derivatives of UiOAZB, a previously studied MOF in the group,[3–5] were employed in this study. **Fig. S45 (a)** represents the original UiOAZB structure, while **Fig. S45 (b)** illustrates the structure of UiOAZB with all fluorinated linkers (AZB-F linkers). In our simulation study, we represent the fluorinated linkers (AZB-F) in red, and non-fluorinated linkers (AZB) in white. A series of 4, 4, 2, and 4 structures were generated for 16.67 %, 33.33 %, 50 %, and 83.33 % MTV structures, respectively, by changing the orientation of AZB-F (in red) and AZB linkers (in white), as shown in **Fig. S46-S49**. An atomistic model structure of two drugs studied here, *viz.* 5-Fluorouracil (5-FU) and Nile-red (NR) is shown in **Fig. S50 (a)** and **(b)** respectively. Due to the fact that inclusion of solvent in drug adsorption simulations remains crucial, these simulations were conducted in the presence of dimethyl formamide (DMF) solvent.

The non-bonded interactions of MOF-drug, drug-drug, drug-DMF, DMF-DMF, and DMF-MOF were represented by Lennard-Jones (LJ) potential. Universal Force Field (UFF) parameters were used to describe the potentials for the MOF atoms, drugs, and DMF. Lorentz–Berthelot (LB)

mixing rules were used and interactions beyond 12 Å were neglected. However, since the simulation adsorption in the presence of DMF was significantly higher than experimental results, we tuned these UFF parameters for both drugs. Specifically the interactions of 5-FU with DMF and MOF was reduced to 35 % of the original UFF values, while NR with DMF and MOF were reduced to 50 %. These modified-UFF interactions for 5-FU and NR are shown in **Table S5** and **S6** respectively. In addition, the interactions of these 5-FU and NR with fluorine of MOF were overridden by using adjusting epsilon and sigma of F (but only with 5-FU and NR). These overridden cross interactions of 5-FU and NR are mentioned in **Tables S7** and **S8.** Moreover, the electrostatic interactions were also included and were described by the coloumb potential. Coulomb interactions were computed by determining interactions through partial charges of MOF determined by charge equilibration method and partial charges of MOF and drug *via.* DDEC partial charges. The calculated charges for drugs and solvent are mentioned in **Table S9** For the calculation of electrostatic interactions, the Ewald sum method was used. To compute the cross interactions, Since the molecule is modeled as rigid, van der Waals interactions within the molecule are ignored. In order to equilibrate the system and calculate the ensemble averages, $1.5x10^6$ Monte Carlo equilibration cycles and $0.5x10^6$ production cycles were used for 5-FU molecules, and $0.5x10^6$ equilibration cycles and $0.5x10^6$ production cycles were used for NR molecules. We allowed Monte Carlo moves on DMF (component 1) and drug (component 2) with equal probabilities of translation, rotation, insertion, deletion, random reinsertion, and regrowth of existing molecules. To mimic the experimental adsorption study, DMF and drug both were allowed to perform these moves simultaneously.

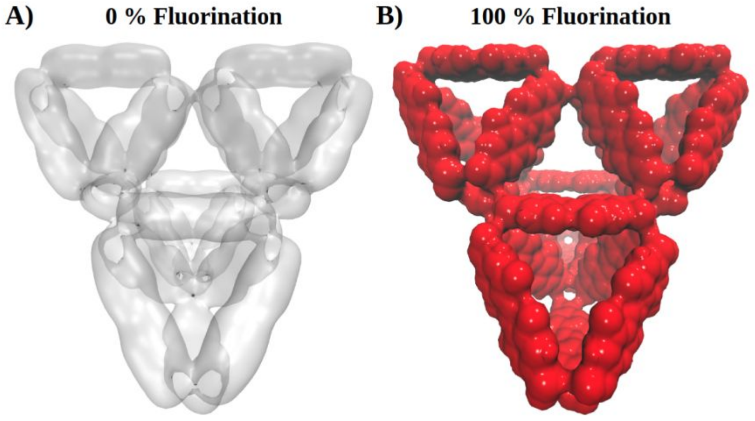

Figure S45 A representation of an unit cell of UiOAZB with (A) 0 linkers fluorinated out of total of 24, which accounts for 0 % fluorination (only AZB linkers), and (B) 24 linkers fluorinated out of total of 24, which accounts for 100 % fluorination (only AZB-F linkers)

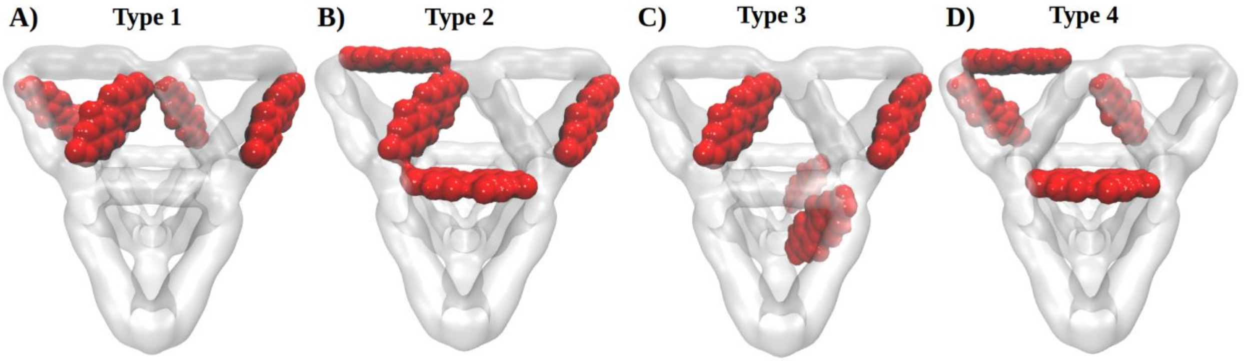

Figure S46 A representation of a unit cell of UiOAZB with 4 linkers fluorinated out of total of 24, which accounts for 16.67 % fluorination. A-D illustrates the orientation of fluorinated linkers (AZB-F) marked in red compared to other non-fluorinated linkers (AZB).

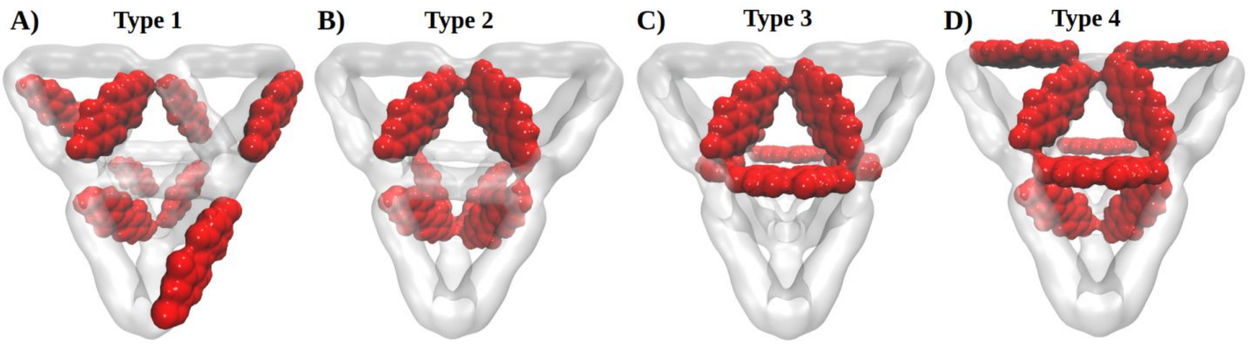

Figure S47 A representation of an unit cell of UiOAZB with 8 linkers fluorinated out of total of 24, which accounts for 33.33 % fluorination. A-D illustrates the orientation of fluorinated linkers (AZB-F) marked in red compared to other non-fluorinated linkers (AZB).

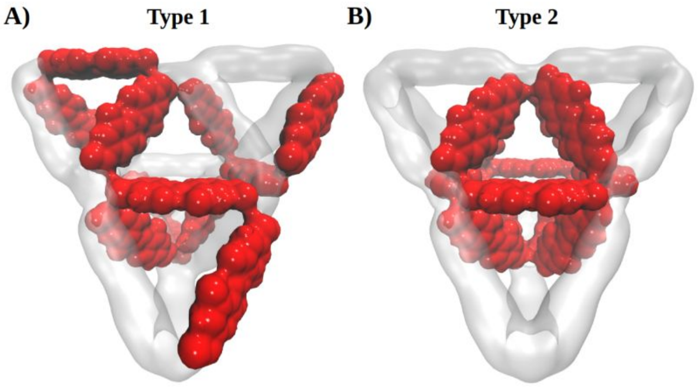

Figure S48 A representation of an unit cell of UiOAZB with 12 linkers fluorinated out of total of 24, which accounts for 50 % fluorination. A-B illustrates the orientation of fluorinated linkers (AZB-F) marked in red compared to other non-fluorinated linkers (AZB).

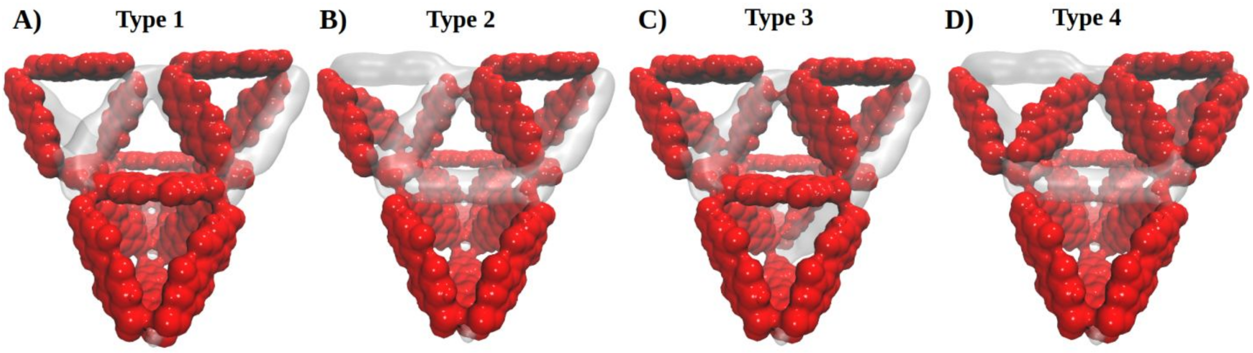

Figure S49 A representation of a unit cell of UiOAZB with 20 linkers fluorinated out of total of 24, which accounts for 83.33 % fluorination. A-D illustrates the orientation of fluorinated linkers (AZB-F) marked in red compared to other non-fluorinated linkers (AZB).

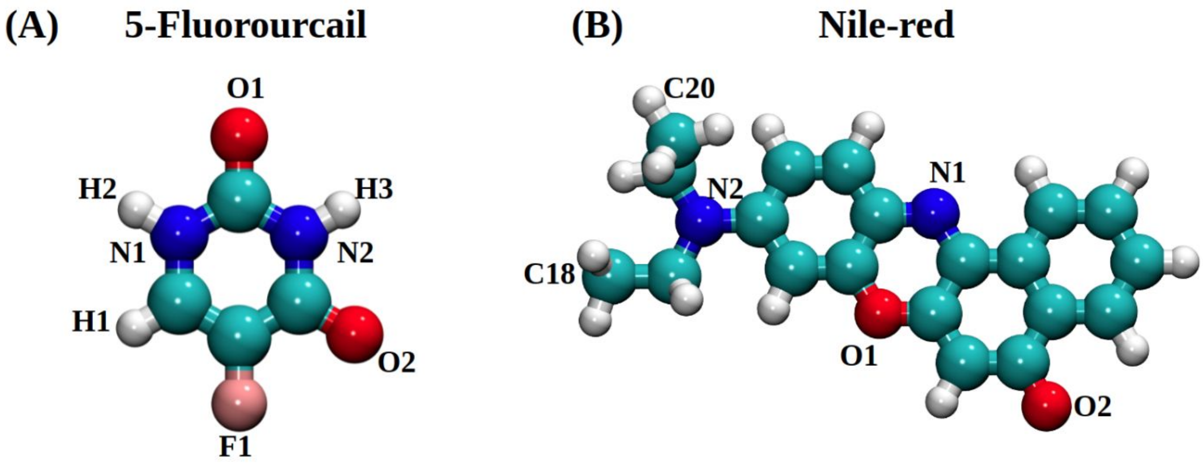

Figure S50 A visual representation of (a) 5-fluorouracil and (b) Nile-red molecules highlighting their important atoms studied in the system.

Table S5. The interactions (epsilon) of 5-FU atoms reduced to 35 % of original UFF values are shown.

**5-Fluorouracil**		
**Name**	**Epsilon (K)**	**Sigma (Å)**
C_1fluor	18.4926	3.431

C_2fluor	18.4926	3.431
N_1fluor	12.15284	3.26256
H_1fluor	7.74935	2.572
C_3fluor	18.4926	3.431
H_2fluor	7.74935	2.572
O_1fluor	10.5672	3.119
N_2fluor	12.15284	3.26256
C_4fluor	18.4926	3.431
H_3fluor	7.74935	2.572
O_2fluor	10.5672	3.119
F_1fluor	8.80635	2.996988757

Table S6. The interactions (epsilon) of NR atoms reduced to 50 % of original UFF values are shown.

Nile-red		
**Name**	**Epsilon (K)**	**Sigma (Å)**
C_1nil	26.418	3.431
C_2nil	26.418	3.431
C_3nil	26.418	3.431
C_4nil	26.418	3.431
C_5nil	26.418	3.431
C_6nil	26.418	3.431
H_1nil	11.0705	2.572
H_2nil	11.0705	2.572
H_3nil	11.0705	2.572
H_4nil	11.0705	2.572
C_7nil	26.418	3.431
C_8nil	26.418	3.431
C_9nil	26.418	3.431
H_5nil	11.0705	2.572
C_10nil	26.418	3.431
N_1nil	17.3612	3.26256

O_1nil	15.096	3.119
C_11nil	26.418	3.431
C_12nil	26.418	3.431
C_13nil	26.418	3.431
C_14nil	26.418	3.431
C_15nil	26.418	3.431
H_6nil	11.0705	2.572
C_16nil	26.418	3.431
H_7nil	11.0705	2.572
H_8nil	11.0705	2.572
N_2nil	17.3612	3.26256
C_17nil	26.418	3.431
C_18nil	26.418	3.431
H_9nil	11.0705	2.572
H_10nil	11.0705	2.572
H_11nil	11.0705	2.572
H_12nil	11.0705	2.572
H_13nil	11.0705	2.572
C_19nil	26.418	3.431
C_20nil	26.418	3.431
H_14nil	11.0705	2.572
H_15nil	11.0705	2.572
H_16nil	11.0705	2.572
H_17nil	11.0705	2.572
H_18nil	11.0705	2.572
O_2nil	15.096	3.119

Table S7. The overridden interactions of 5-FU with F of MOF are shown.

Overridden cross-interactions of 5-FU and F of MOF			
**MOF atoms**	**NR atoms**	**Epsilon (K)**	**Sigma (Å)**
F_	C_1fluor	21.57063533	3.0655

F_	C_2fluor	21.57063533	3.0655
F_	N_1fluor	17.48649786	2.98128
F_	H_1fluor	13.96357387	2.636
F_	C_3fluor	21.57063533	3.0655
F_	H_2fluor	13.96357387	2.636
F_	O_1fluor	16.30586763	2.9095
F_	N_2fluor	17.48649786	2.98128
F_	C_4fluor	21.57063533	3.0655
F_	H_3fluor	13.96357387	2.636
F_	O_2fluor	16.30586763	2.9095
F_	F_1fluor	14.88544834	2.848494379

Table S8. The overridden interactions of NR with F of MOF are shown.

**Overridden cross-interactions of NR and F of MOF**			
**MOF atoms**	**NR atoms**	**Epsilon (K)**	**Sigma (Å)**
F_	C_1nil	27.19749989	3.213994379
F_	C_2nil	27.19749989	3.213994379
F_	C_3nil	27.19749989	3.213994379
F_	C_4nil	27.19749989	3.213994379
F_	C_5nil	27.19749989	3.213994379
F_	C_6nil	27.19749989	3.213994379
F_	H_1nil	17.6060785	2.784494379
F_	H_2nil	17.6060785	2.784494379
F_	H_3nil	17.6060785	2.784494379
F_	H_4nil	17.6060785	2.784494379
F_	C_7nil	27.19749989	3.213994379
F_	C_8nil	27.19749989	3.213994379
F_	C_9nil	27.19749989	3.213994379
F_	H_5nil	17.6060785	2.784494379
F_	C_10nil	27.19749989	3.213994379
F_	N_1nil	22.04798403	3.129774379

F_	O_1nil	20.55937742	3.057994379
F_	C_11nil	27.19749989	3.213994379
F_	C_12nil	27.19749989	3.213994379
F_	C_13nil	27.19749989	3.213994379
F_	C_14nil	27.19749989	3.213994379
F_	C_15nil	27.19749989	3.213994379
F_	H_6nil	17.6060785	2.784494379
F_	C_16nil	27.19749989	3.213994379
F_	H_7nil	17.6060785	2.784494379
F_	H_8nil	17.6060785	2.784494379
F_	N_2nil	22.04798403	3.129774379
F_	C_17nil	27.19749989	3.213994379
F_	C_18nil	27.19749989	3.213994379
F_	H_9nil	17.6060785	2.784494379
F_	H_10nil	17.6060785	2.784494379
F_	H_11nil	17.6060785	2.784494379
F_	H_12nil	17.6060785	2.784494379
F_	H_13nil	17.6060785	2.784494379
F_	C_19nil	27.19749989	3.213994379
F_	C_20nil	27.19749989	3.213994379
F_	H_14nil	17.6060785	2.784494379
F_	H_15nil	17.6060785	2.784494379
F_	H_16nil	17.6060785	2.784494379
F_	H_17nil	17.6060785	2.784494379
F_	H_18nil	17.6060785	2.784494379
F_	O_2nil	20.55937742	3.057994379

II. Simulation Results

The adsorption results for both 5-FU and NR are tabulated in Tables S5 and S6 respectively. The yellow marked cells represent the experimental adsorption values. Moreover, the simulation results are tabulated for all structures of MTV MOFs. The dark green colored

highlighted structures denote the structures with simulation adsorption best matching with the experimental adsorption. This structure was used in further analyses reported here and in main manuscript.

Table S9. 5-FU loading in the simulations in the presence of DMF solvent for all systems studied.

**5-Fluorouracil**							
		100	79 ± 5	53 ± 1	32 ± 3	18 ± 1	0
**MOF % Fluorination**		100	83.33	50	33.33	16.67	0
**Experimental 5FU adsorption**	**(wt. %)**	**11 ± 2**	**6 ± 1**	**14 ± 2**	**21 ± 1**	**4 ± 1**	0
**Simulations**	MOF						
**5-FU GCMC results (wt. %)**	Type 1	4.93	8.93	5.36	14.4	7.29	3.34
	Type 2		1.79	11.9	18.12	10.13	
	Type 3		2.52		13.27	9.11	
	Type 4		0.78		7.7	14.75	
**Average**		4.93	3.505	8.63	13.37	10.32	3.34
**Standard deviation**			3.69	4.62	4.31	3.18	

Table S10. NR loading in the simulations in the presence of DMF solvent for all systems studied.

**Nile-red**							
		100	79 ± 5	53 ± 1	32 ± 3	18 ± 1	0
**% Fluorination**		100	83.33	50	33.33	16.67	0
**Experimental NR adsorption**	**(wt. %)**	**4 ± 2**	**10 ± 2**	**7 ± 3**	**6 ± 1**	**6 ± 3**	**3 ± 1**
**Simulations**	MOF						
**NR GCMC results (wt. %)**	Type 1	5.84	12.01	9.52	13.08	6.74	3.47
	Type 2		3	9.52	3.27	16.85	
	Type 3		9.01		13.08	10.1	
	Type 4		6.01		13.08	6.74	
		5.84	7.5075	9.52	10.6275	10.1075	3.47
			3.88	0	4.90	4.76	

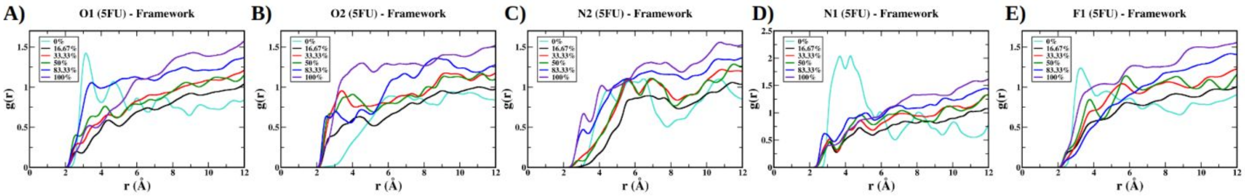

Figure S51. RDF of selected atoms of 5-Fluorouracil with the whole Framework (for all MTV MOFs).

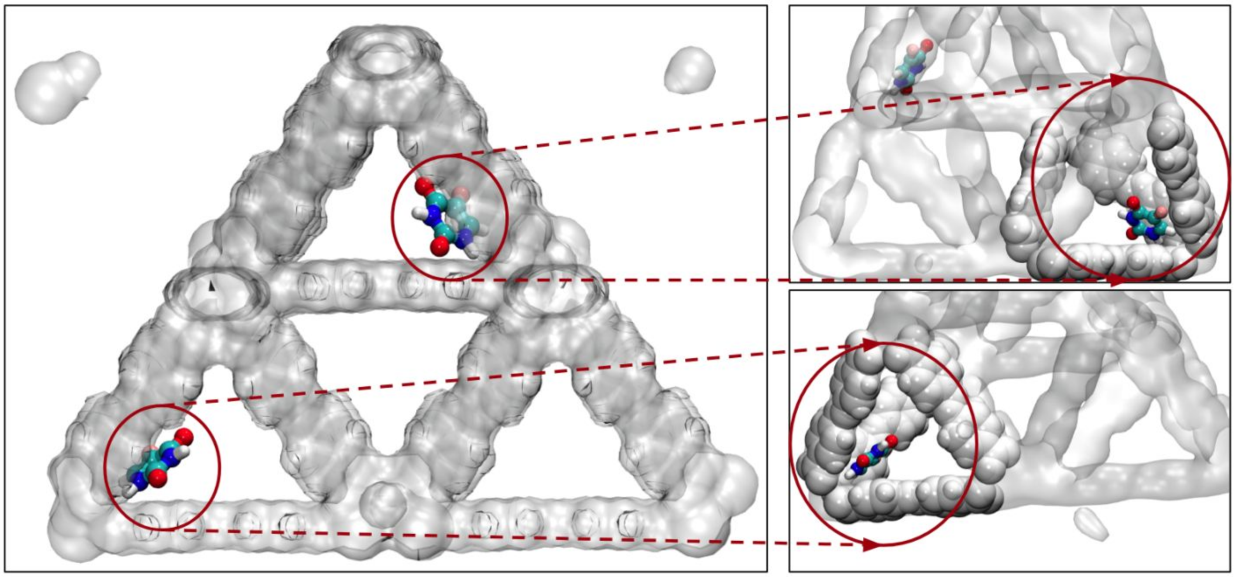

Figure S52. Snapshots of 5-FU crowded into metal node corner sites of 0 % MTV MOF.

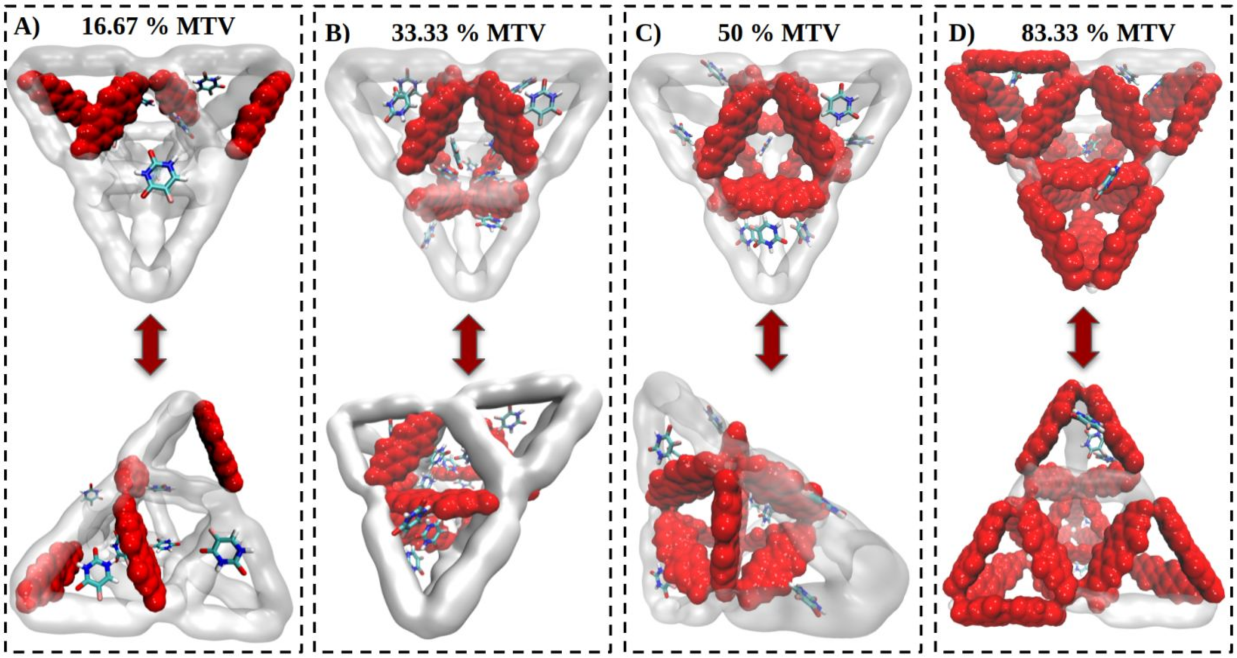

Figure S53. Snapshots of the sites where adsorption occurred and the orientation of 5-FU in (a) 16.67 % MTV MOF, (a) 33.33 % MTV MOF, (c) 50 % MTV MOF, and (d) 83.33 % MTV MOF. The top and bottom rows show the same figure with different angles and ways of representation of atoms. The pink, red, white, and blue atoms denote the fluorine, hydrogen, oxygen, and nitrogen atoms respectively.

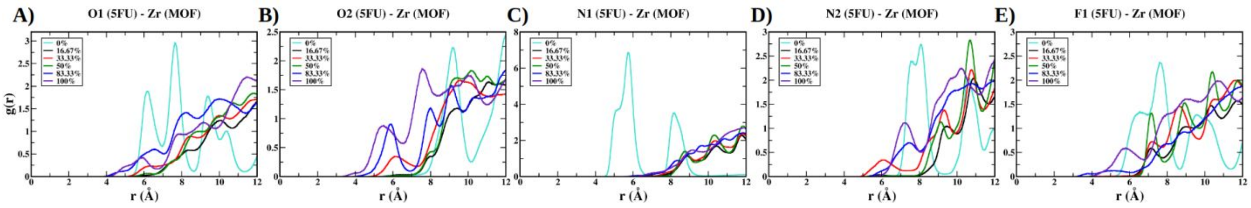

Figure S54. RDF of selected atoms of 5-Fluorouracil with the metal sites of all MTV MOFs.

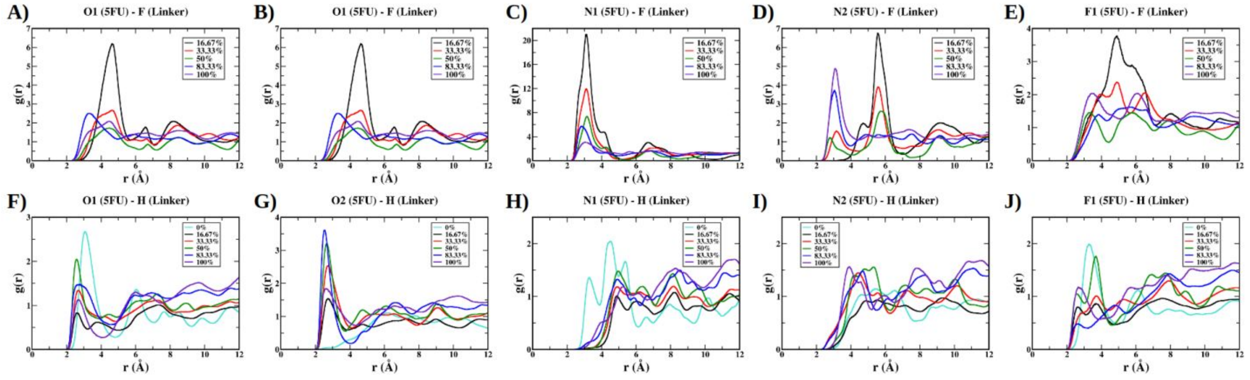

Figure S55. RDF of selected atoms of 5-Fluorouracil with fluorine and hydrogen of all MTV MOFs.

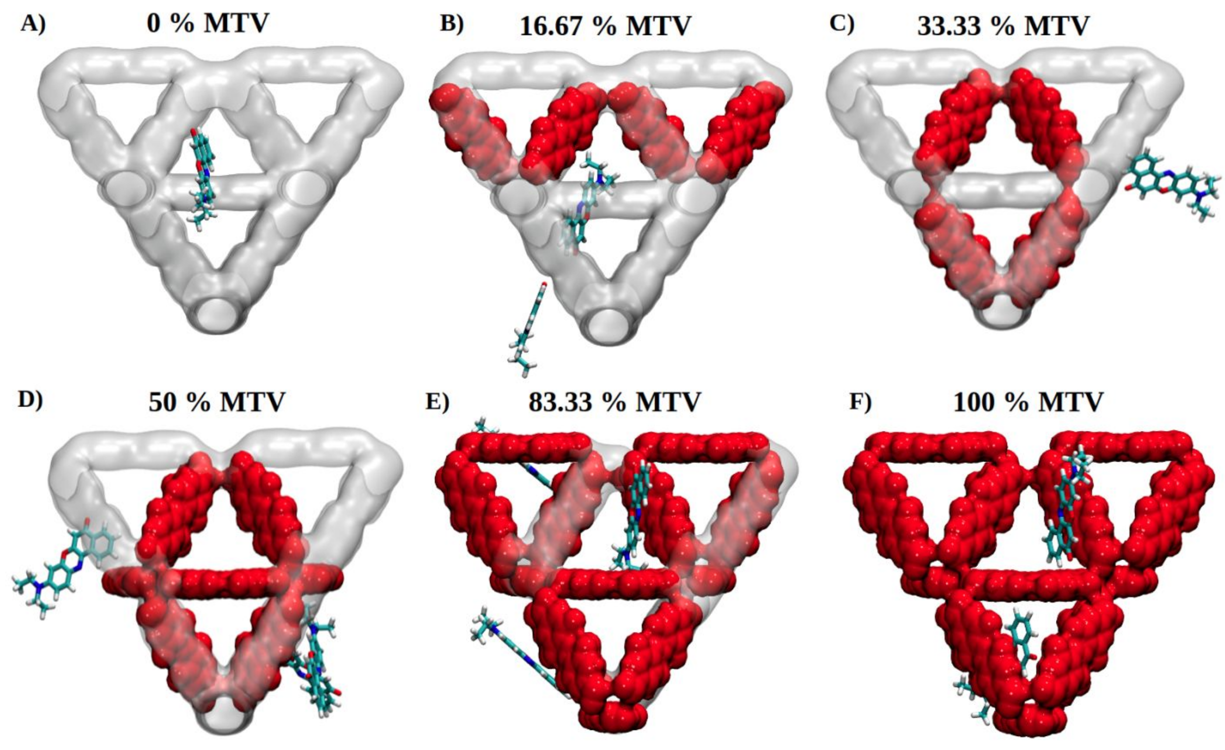

Figure S56. Snapshots of the selected **(a-f)** 0 % - 100 % MTV MOFs which exhibited consistency with experimental adsorption of NR molecules.

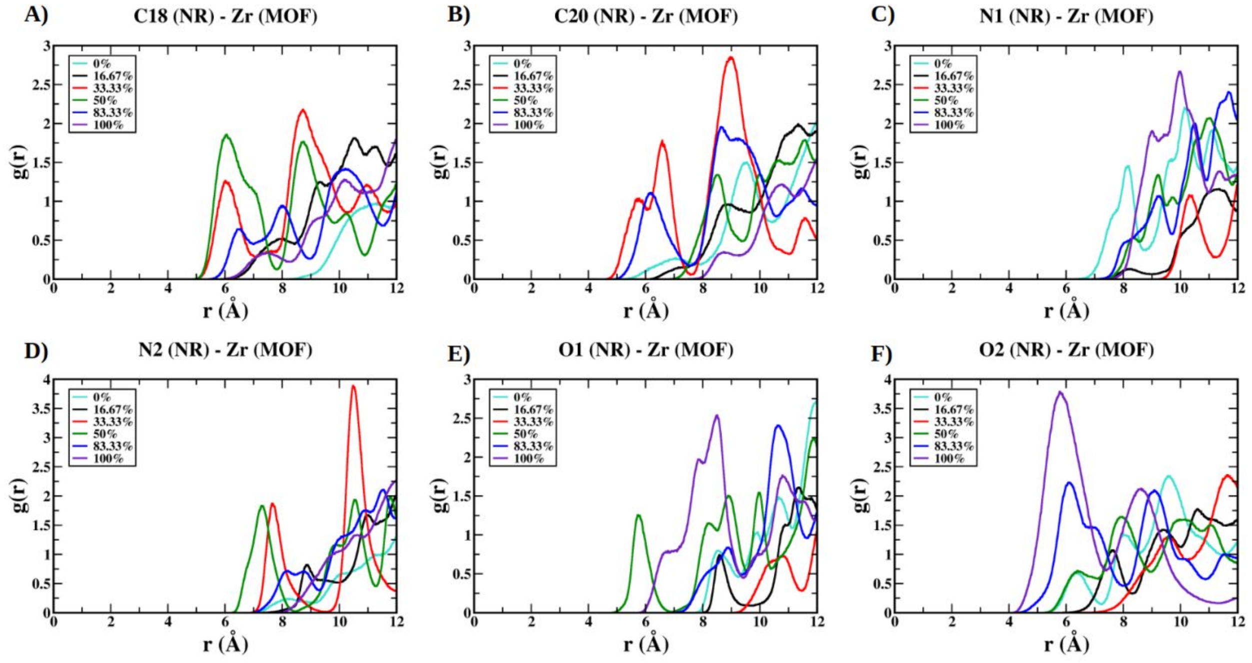

Figure S57. RDF of selected atoms of Nile-red with the metal site of all MTV MOFs.

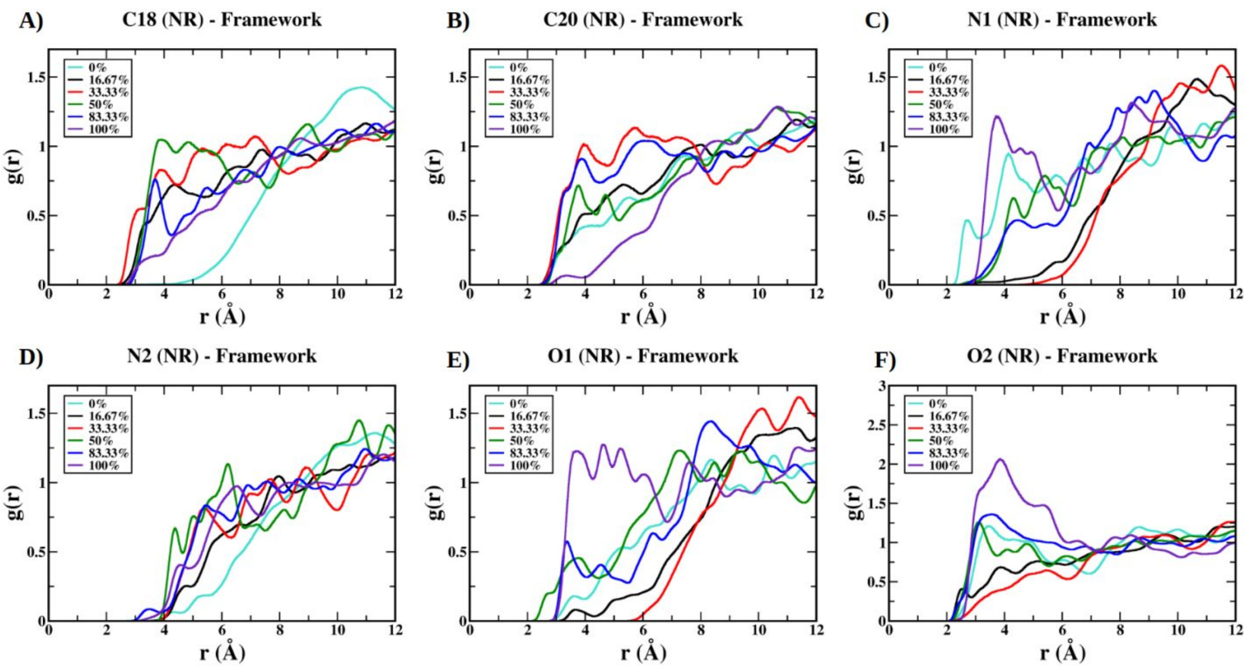

Figure S58. RDF of selected atoms of Nile-red with the whole Framework (for all MTV MOFs).

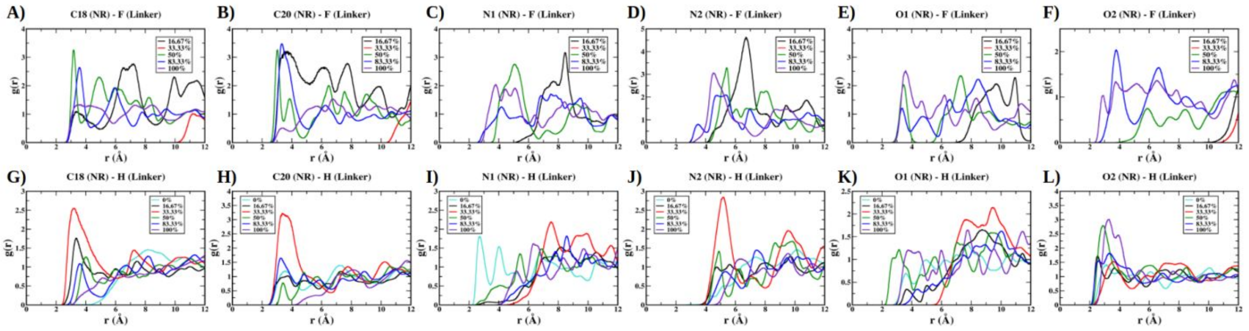

Figure S59. RDF of selected atoms of Nile-red with the fluorine and hydrogens of all MTV MOFs.

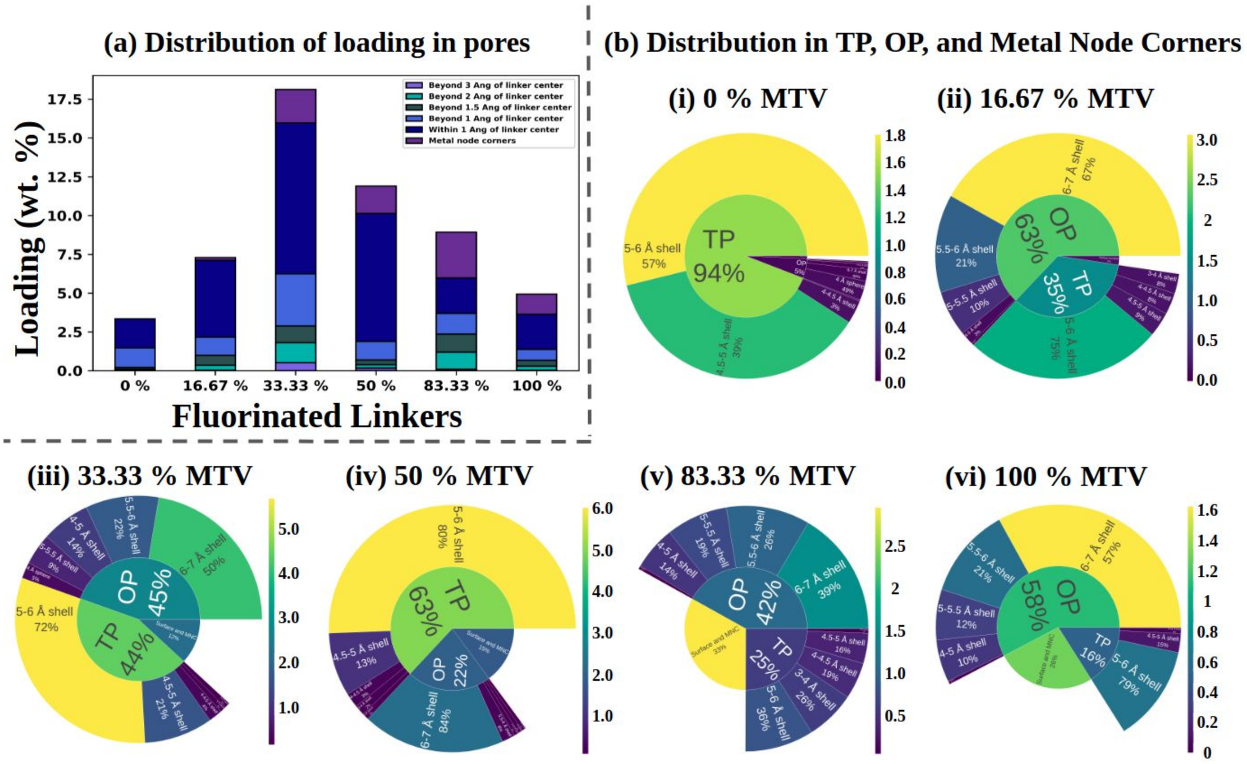

Figure S60. Distribution of 5-FU loading inside the pores of MTV MOF. (b) A nested pie chart to represent the distribution of 5-FU loading inside tetrahedral pores (inner circle) and octahedral pores (outer circle) in terms of percentages.

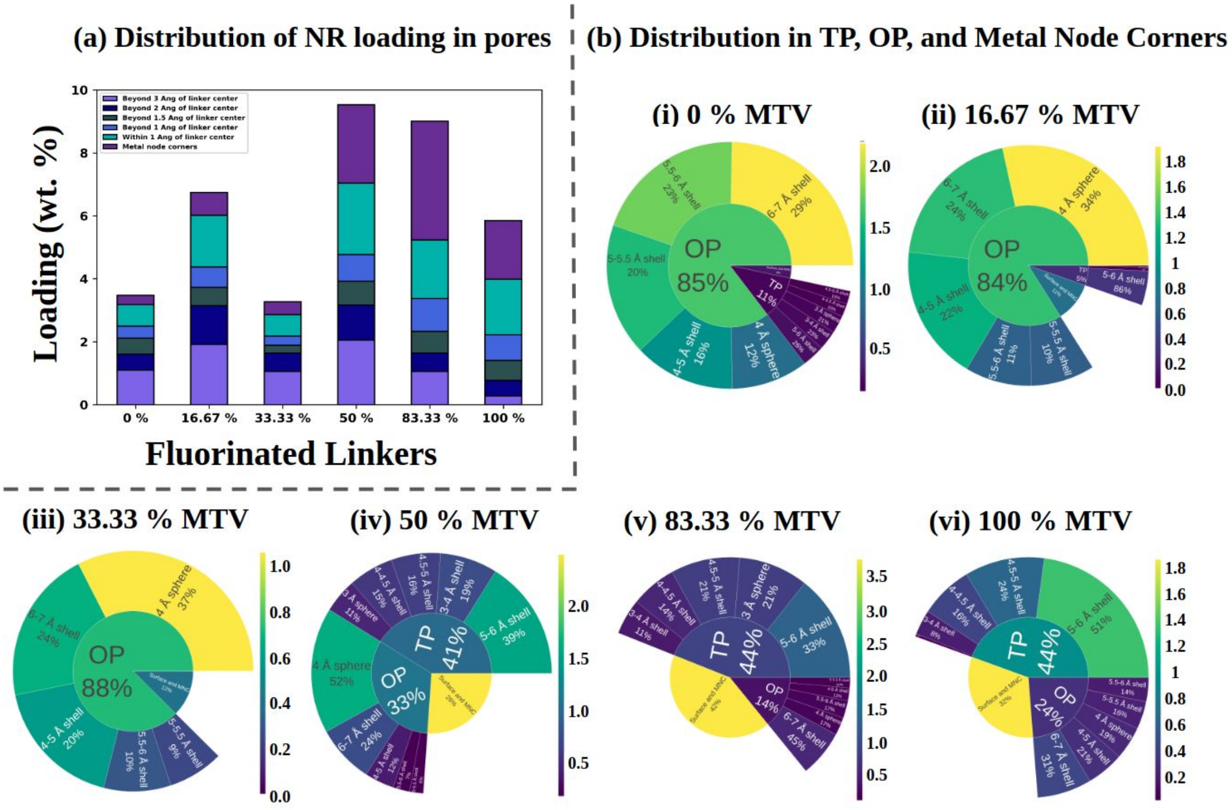

Figure S61. Distribution of NR loading inside the pores of MTV MOF. (b) A nested pie chart to represent the distribution of NR loading inside tetrahedral pores (inner circle) and octahedral pores (outer circle) in terms of percentages.

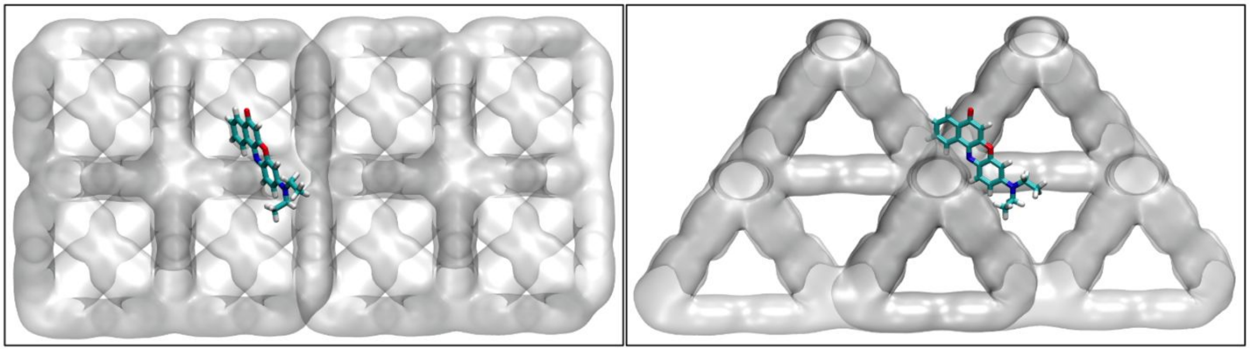

Figure S62. Snapshots of NR adsorption in 0 % MTV MOF

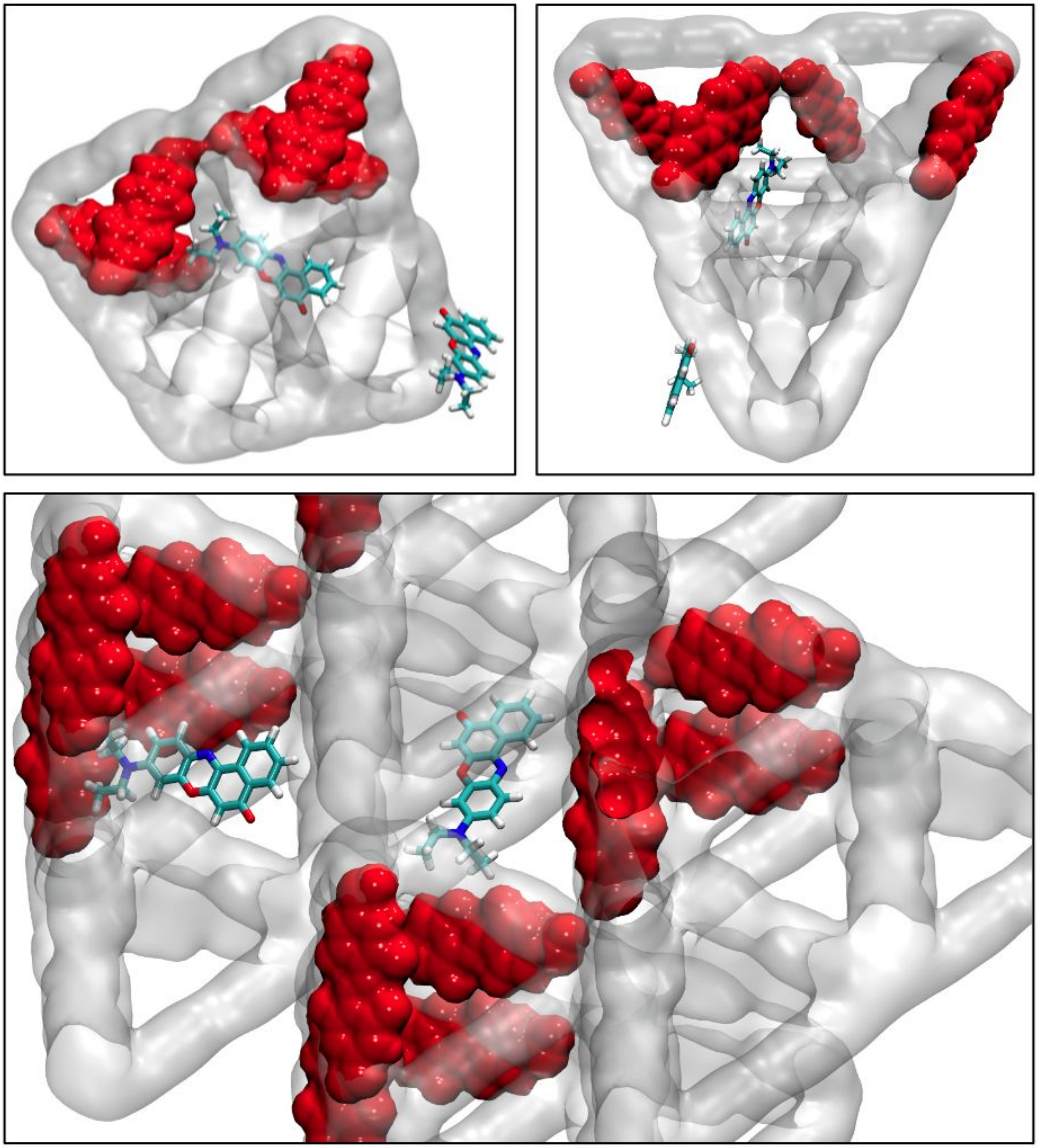

Figure S63. Snapshots of NR adsorption in 16.67 % MTV MOF

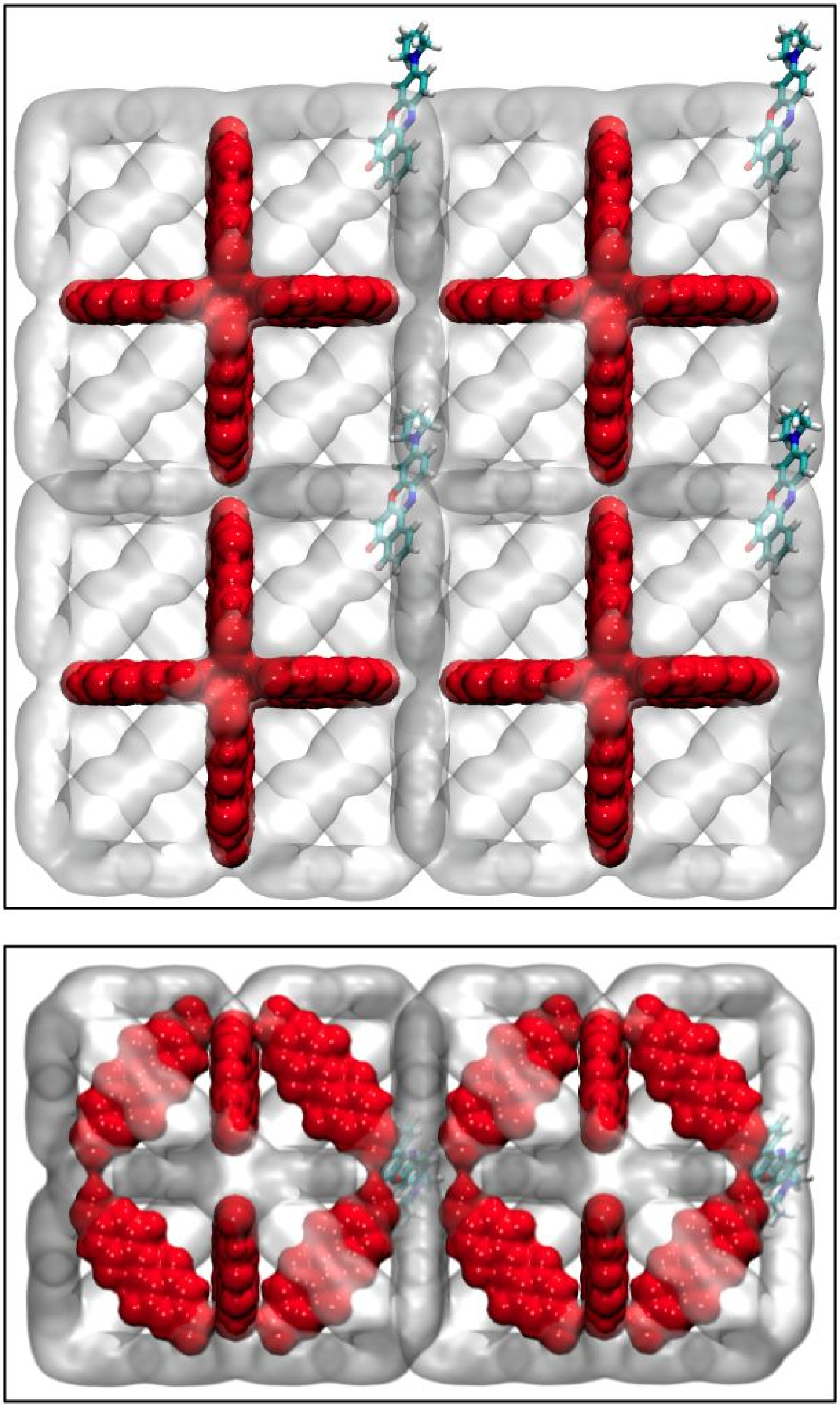

Figure S64. Snapshots of NR adsorption in 33.33 % MTV MOF

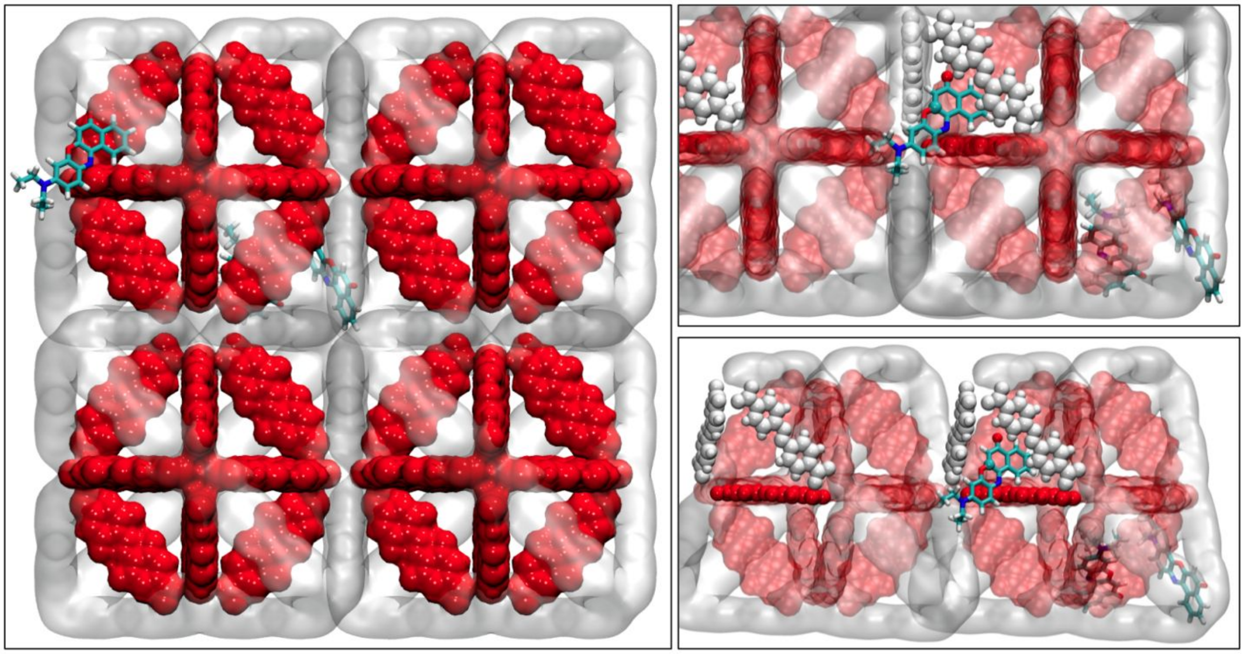

Figure S65. Snapshots of NR adsorption in 50 % MTV MOF

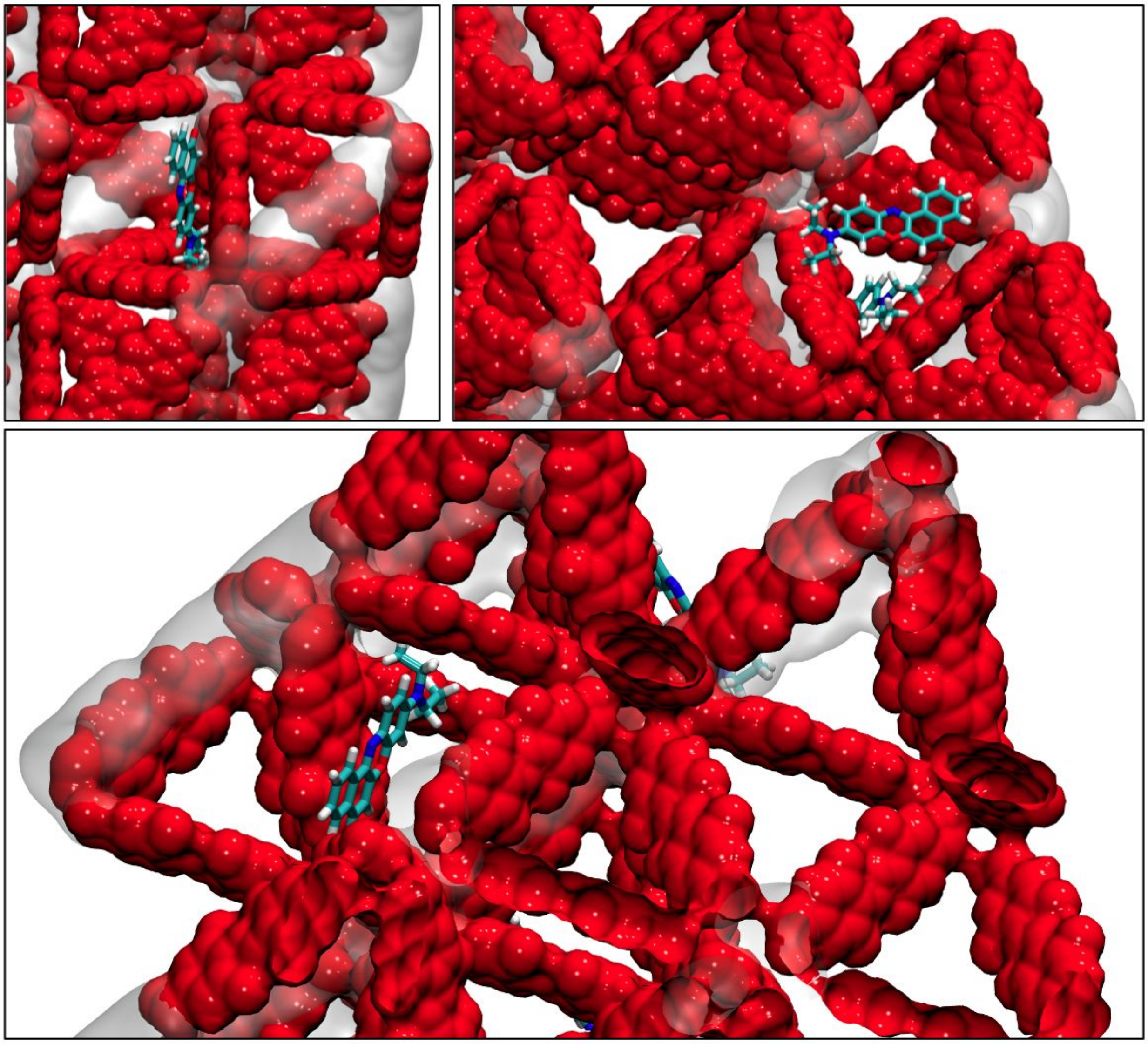

Figure S66. Snapshots of NR adsorption in 83.33 % MTV MOF

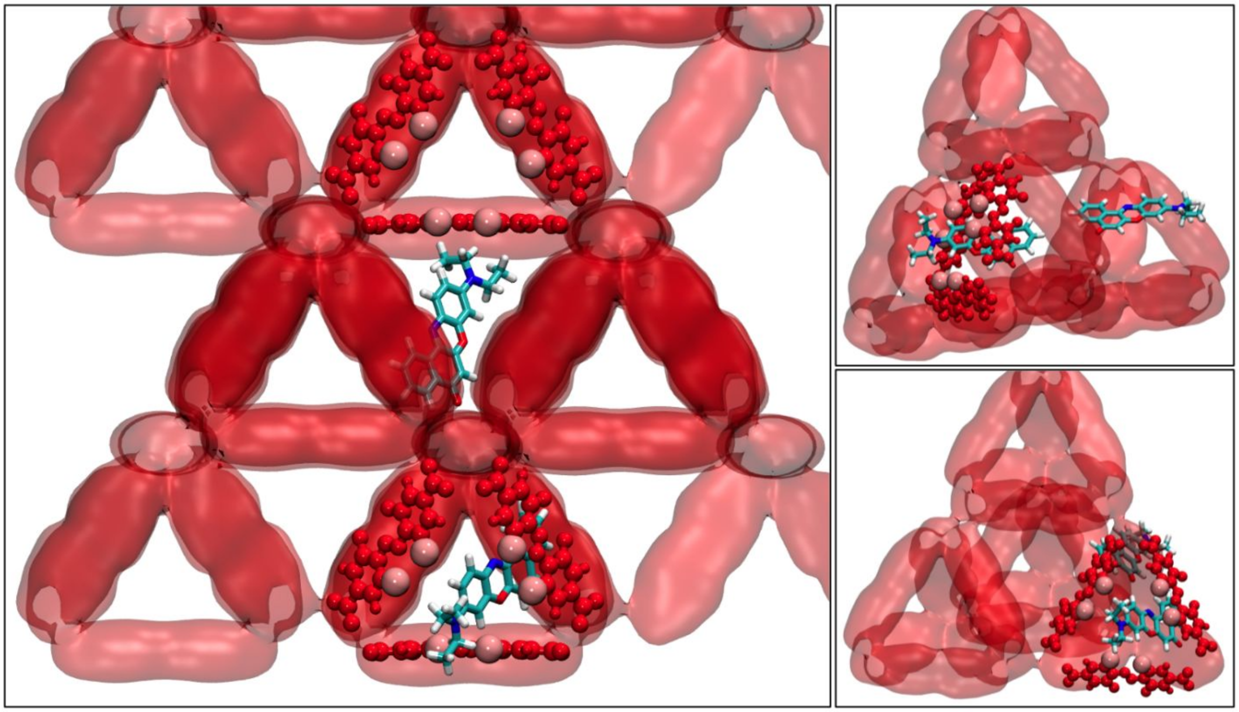

Figure S67. Snapshots of NR adsorption in 100 % MTV MOF

References:

(1) Okumura, S.; Lin, C.-H.; Takeda, Y.; Minakata, S. Oxidative Dimerization of (Hetero)Aromatic Amines Utilizing t-BuOI Leading to (Hetero)Aromatic Azo Compounds: Scope and Mechanistic Studies. *J. Org. Chem.* **2013**, *78* (23), 12090–12105. https://doi.org/10.1021/jo402120w.

(2) Heinrich, B.; Bouazoune, K.; Wojcik, M.; Bakowsky, U.; Vázquez, O. Ortho-Fluoroazobenzene Derivatives as DNA Intercalators for Photocontrol of DNA and Nucleosome Binding by Visible Light. *Org. Biomol. Chem.* **2019**, *17* (7), 1827–1833. https://doi.org/10.1039/C8OB02343C.

(3) Sose, A. T.; Cornell, H. D.; Gibbons, B. J.; Burris, A. A.; Morris, A. J.; Deshmukh, S. A. Modelling Drug Adsorption in Metal–Organic Frameworks: The Role of Solvent. *RSC Adv.* **2021**, *11* (28), 17064–17071. https://doi.org/10.1039/D1RA01746B.

(4) Epley, C. C.; Roth, K. L.; Lin, S.; Ahrenholtz, S. R.; Grove, T. Z.; Morris, A. J. Cargo Delivery on Demand from Photodegradable MOF Nano-Cages. *Dalton Trans.* **2017**, *46* (15), 4917–4922. https://doi.org/10.1039/C6DT04787D.

(5) Stefaniak, K. R.; Epley, C. C.; Novak, J. J.; McAndrew, M. L.; Cornell, H. D.; Zhu, J.; McDaniel, D. K.; Davis, J. L.; Allen, I. C.; Morris, A. J.; Grove, T. Z. Photo-Triggered Release of 5-Fluorouracil from a MOF Drug Delivery Vehicle. *Chem. Commun.* **2018**, *54* (55), 7617–7620. https://doi.org/10.1039/C8CC01601A.
